# Novel Genetic Loci Identified for the Pathophysiology of Childhood Obesity in the Hispanic Population

**DOI:** 10.1371/journal.pone.0051954

**Published:** 2012-12-14

**Authors:** Anthony G. Comuzzie, Shelley A. Cole, Sandra L. Laston, V. Saroja Voruganti, Karin Haack, Richard A. Gibbs, Nancy F. Butte

**Affiliations:** 1 Department of Genetics, Texas Biomedical Research Institute, San Antonio, Texas, United States of America; 2 Human Genome Sequencing Center, Department of Molecular & Human Genetics, Baylor College of Medicine, Houston, Texas, United States of America; 3 USDA/ARS Children’s Nutrition Research Center, Department of Pediatrics, Baylor College of Medicine, Houston, Texas, United States of America; Vanderbilt University, United States of America

## Abstract

Genetic variants responsible for susceptibility to obesity and its comorbidities among Hispanic children have not been identified. The VIVA LA FAMILIA Study was designed to genetically map childhood obesity and associated biological processes in the Hispanic population. A genome-wide association study (GWAS) entailed genotyping 1.1 million single nucleotide polymorphisms (SNPs) using the Illumina Infinium technology in 815 children. Measured genotype analysis was performed between genetic markers and obesity-related traits i.e., anthropometry, body composition, growth, metabolites, hormones, inflammation, diet, energy expenditure, substrate utilization and physical activity. Identified genome-wide significant loci: 1) corroborated genes implicated in other studies (*MTNR1B, ZNF259/APOA5, XPA/FOXE1 (TTF-2), DARC, CCR3, ABO*); 2) localized novel genes in plausible biological pathways (*PCSK2, ARHGAP11A, CHRNA3*); and 3) revealed novel genes with unknown function in obesity pathogenesis (*MATK*, *COL4A1*). Salient findings include a nonsynonymous SNP (rs1056513) in *INADL* (*p* = 1.2E-07) for weight; an intronic variant in *MTNR1B* associated with fasting glucose (*p* = 3.7E-08); variants in the *APOA5-ZNF259* region associated with triglycerides (*p* = 2.5-4.8E-08); an intronic variant in *PCSK2* associated with total antioxidants (*p* = 7.6E-08); a block of 23 SNPs in *XPA/FOXE1* (*TTF-2*) associated with serum TSH (*p* = 5.5E-08 to 1.0E-09); a nonsynonymous SNP (*p* = 1.3E-21), an intronic SNP (*p* = 3.6E-13) in *DARC* identified for MCP-1; an intronic variant in *ARHGAP11A* associated with sleep duration (*p* = 5.0E-08); and, after adjusting for body weight, variants in *MATK* for total energy expenditure (*p* = 2.7E-08) and in *CHRNA3* for sleeping energy expenditure (*p* = 6.0E-08). Unprecedented phenotyping and high-density SNP genotyping enabled localization of novel genetic loci associated with the pathophysiology of childhood obesity.

## Introduction

Obesity is a complex disease influenced by genetic and environmental factors and their interactions. The current surge in childhood obesity in the U.S. is attributable to an interaction between a genetic predisposition toward efficient energy storage and a permissive environment of readily available food and sedentary behaviors [1]. Genetic architecture of common polygenic childhood obesity remains largely unknown. In genetic studies, the phenotypic description of the obese child usually has been limited to body mass index (BMI). BMI represents a composite trait of fat free mass (FFM) and fat mass (FM) and thus loci influencing BMI may differ from more direct measures of adiposity. In addition, markers of biological processes underlying the development of obesity such as dietary intake, energy expenditure and nutrient partitioning may be more effectual in identifying causal genetic variants [2]. In epidemiology studies, childhood obesity has been shown to be genetically correlated with glucose intolerance, hypertension, dyslipidemia, insulin resistance, chronic inflammation, and risk for fatty liver disease [3], [4]. Identification of genes underlying these distinct patterns of association also may unravel important biological pathways involved in the pathophysiology of childhood obesity.

Genome-wide association studies (GWAS) have the potential to localize genetic loci contributing to obesity down to a few 100 kb [5]. In fact, a recent meta-analysis of the adult GIANT Consortium established 32 susceptibility BMI loci [6], several of which were confirmed in French and German children with extreme obesity [7] and European adults with early-onset obesity [8]. Two novel loci near *OLFM4* and within *HOXB5* were recently reported based on a meta-analysis of 14 pediatric studies of BMI [9]. These pediatric GWAS were confined to BMI and cohorts of European ancestry.

Here, we present findings from a GWAS designed to identify genetic variants influencing childhood obesity and its comorbidities in the Hispanic population. We have published evidence of heritability, pleiotropy amongst traits, and chromosomal regions implicated in obesity among Hispanic children in our VIVA LA FAMILIA Study [4], [10]–[15] In-depth phenotyping was performed to characterize the children, including anthropometry, body composition, growth, metabolites, hormones, inflammation, diet, energy expenditure and substrate utilization and physical activity. Our high-density SNP genotyping and phenotypes representing not only adiposity, but also biological processes associated with the development and consequences of childhood obesity enabled localization of novel genetic loci associated with the pathophysiology of childhood obesity.

## Materials and Methods

The VIVA LA FAMILIA Study was designed to identify genetic variants influencing pediatric obesity and its comorbidities. Family recruitment and phenotyping were conducted in 2000–2005 in Houston, TX. All enrolled children and parents gave written informed consent or assent. The protocol was approved by the Institutional Review Boards for Human Subject Research for Baylor College of Medicine and Affiliated Hospitals and for Texas Biomedical Research Institute.

The VIVA LA FAMILIA study design and methodology have been described in detail elsewhere^4^. GWAS was performed on 815 children from 263 Hispanic families. The number of families by sibships was: 8 (one child), 40 (two children), 155 (three children), 48 (four children), 6 (five children), 3 (six children), 2 (seven children) and 1 (eight children). Each family was ascertained on an obese proband, defined as a BMI>95^th^ percentile, between the ages 4–19 y. Once identified, the obese proband and all siblings, 4 to 19 y of age, and their parents were invited to the Children’s Nutrition Research Center for a tour and full explanation of the study prior to consenting. The cross-sectional, longitudinal study design consisted of baseline measurements, with a one-year follow-up to track children’s growth and body compositional changes. In-depth baseline phenotyping included vital signs, anthropometry and body composition, diet and physical fitness, 24-h calorimetry, eating behavior, physical activity, fasting blood sampling for DNA and other biochemistries.

Briefly, blood pressure, heart rate and temperature were taken using an automated monitor. Anthropometric measurements were performed using standardized techniques according to Lohman [16]. Body composition was determined by dual-energy x-ray absorptiometry. Repeated measures after one year were used to compute growth velocities and changes in FM, FFM and energy storage [17]. Methods used to measure fasting blood and 24-h urinary biochemistries are described elsewhere [14], [18], [19]. A multiple-pass 24-h dietary recall was recorded on two occasions using Nutrition Data System (NDS) [20]. Eating behavior was assessed with a dinner meal and eating in the absence of hunger [21]. Room respiration calorimetry was used to make 24-h measurements of energy expenditure and substrate oxidation [13]. Maximum oxygen consumption (VO_2_max) and heart rate maximum (HRmax) were measured on a treadmill [22]. Actiwatch accelerometers were used to measure frequency, duration and intensity of physical activity [23].

### Genotyping

The Illumina HumanOmni1-Quad v1.0 BeadChips were used to genotype 1.1 million single nucleotide polymorphisms (SNPs) in 815 children enrolled in the VIVA LA FAMILIA Study. Genotype calls were obtained after scanning on the Illumina BeadStation 500GX and analysis using the GenomeStudio software. Our genotyping error rate (based on duplicates) was 2 per 100,000 genotypes. Illumina has included sample-dependent and -independent controls on their BeadChips to test for accuracy of the procedure. The average call rate for all SNPs per individual sample was 97%.

SNP genotypes were checked for Mendelian consistency using the program SimWalk2 [24]. The estimates of the allele frequencies and their standard errors were obtained using SOLAR [25]. Specific SNPs were removed from analysis if they had call rates <95% (n = 6,596), were uncommon alleles in less than 5 participants (26,537), deviated from Hardy-Weinberg equilibrium (n = 0), or were monoallelic (n = 56,448). The number of SNPs that passed quality control and were included in the GWA analysis was 899,892.

### Genome-wide Association Analysis

Measured genotype analysis (MGA) was performed using the SOLAR program [25]. All phenotypes were transformed by inverse normalization to meet assumptions of normality. We obtained residuals using linear regression models adjusted for age, sex, their interaction and higher order terms. Because energy expenditure is strongly influenced by body weight across the age range of 4 to 19 years of age, total energy expenditure and sleeping energy expenditure were additionally adjusted for body weight. Also, observed energy intakes consumed in a snack and dinner were adjusted for total energy expenditure or estimated energy requirement, again to compensate for the wide range of ages in our cohort.

Each SNP genotype was converted in SOLAR to a covariate measure equal to 0, 1, or 2 copies of the minor allele (or, for missing genotypes, the weighted covariate based on imputation). These covariates were included in the variance-components mixed models for measured genotype analyses [26] versus null models that incorporated the random effect of kinship and fixed effects such as age, sex, their interaction and higher order terms. For the initial GWA screen, we tested each SNP covariate independently as a 1 degree of freedom likelihood ratio test. The p-value threshold for genome-wide significance (alpha = 0.05) was set at 1.01×10^−7^. The p-value threshold for genome-wide significance was computed for our family-based cohort that takes into account pedigree structure. The effective number of SNPs given linkage disequilibrium (LD) was calculated by the method of Moskvina and Schmidt [27] as implemented in SOLAR. LD was computed in SOLAR using all available information (all genotyped SNPs on all individuals). The average ratio of SNP effective number/actual number obtained from analysis of 1,989 non-overlapping bins of SNPs was used to calculate the genome-wide effective number of tests and thus the significance threshold for genome-wide association. We performed quantitative transmission disequilibrium test (implemented in SOLAR) to test for population stratification.

## Results

GWAS was performed on 815 children from 263 Hispanic families. Mean ± SD (range) was 25.2±7.5 (13.4 to 61.9) for the children’s BMI, 85.6±20.8 (4 to100) for BMI percentile and 1.52±1.01 (−3.0 to 4.5) for BMI z-score. Measured genotype analysis examined 129 obesity-related traits including BMI and adiposity as well as biological processes associated with the pathophysiology of childhood obesity; a description of the phenotypes is provided in **Table S1**. In our GWAS, population stratification was not significant and therefore did not confound our associations. A complete listing of all suggestive (*p*<1.0E-06) and genome-wide significant (p<1.0E-07) genetic variants and their associated traits are presented by chromosomal position in **Table S2**.

### Anthropometry and Body Composition

BMI status, body composition and the growth process were assessed from repeated measurements of body weight, height, FFM and FM at baseline and after one-year (**Table 1**). A nonsynonymous SNP (rs1056513; G1178S (NP_005790.2)) in *INADL* on chromosome 1 attained near genome-wide significance (*p* = 1.2E-07) for weight, and BMI, FFM, FM, trunk FM, and hip circumference (p = 8.3E-06 to 1.6E-07). SNP rs1056513 is common (MAF = 0.50) and accounted for 3% of the variance in body weight and body composition in this cohort. Weight z-score change was significantly associated with an intronic variant in *COL4A1* on chromosome 13 *(p = *4.7E-08) (Supporting Information **Figure S1**). Linear growth (height change) was associated with a variant in the 5′UTR region of *TSEN34* on chromosome 19 (*p* = 4.5E-08).

**Table 1:** Measured genotype analysis for anthropometric and body composition traits.

SNP	Chr	Coordinate(GB 36.2)	Location(GB 36.2)	SNP (GB36.2)	Trait	MGAp-value	EffectSize	Minor Allele	Minor Allele Frequency	Gene Symbol	Gene Name
rs1056513	1	62152886	coding-nonsyn	[A/G]	Weight (kg)	1.18E-07	0.031	A	0.495	*INADL*	InaD-like (Drosophila)
rs1056513	1	62152886	coding-nonsyn	[A/G]	BMI (kg/m^2^)	8.34E-06	0.021	A	0.495	*INADL*	InaD-like (Drosophila)
rs1056513	1	62152886	coding-nonsyn	[A/G]	Fat mass (kg)	1.59E-07	0.035	A	0.495	*INADL*	InaD-like (Drosophila)
rs1056513	1	62152886	coding-nonsyn	[A/G]	Trunk fat mass (kg)	2.36E-07	0.035	A	0.495	*INADL*	InaD-like (Drosophila)
rs1056513	1	62152886	coding-nonsyn	[A/G]	Fat free mass (kg)	2.80E-07	0.034	A	0.495	*INADL*	InaD-like (Drosophila)
rs1056513	1	62152886	coding-nonsyn	[A/G]	Hip circumference (cm)	2.47E-06	0.022	A	0.495	*INADL*	InaD-like (Drosophila)
rs494558	13	109727163	intron	[T/C]	Weight z-score change (SD/y)	4.66E-08*	0.045	G	0.078	*COL4A1*	collagen, type IV, alpha 1
rs40357	19	59385339	5UTR	[T/C]	Height change (cm/y)	4.49E-08*	0.045	G	0.391	*TSEN34*	tRNA splicing endonuclease 34homolog (S. cerevisiae)

Abbreviations: SNP, single nucleotide polymorphism; chr, chromosome; MGA, measured genotype analysis; nonsyn, nonsynonomous; BMI, body mass index; UTR, untranslated region.

* Significant according to the widely used significance threshold p-value of <5×10^−8^.

### Endometabolic Traits

Genome-wide significant variants were identified for several endometabolic traits (**Table 2**). An intronic variant in *MTNR1B* on chromosome 11 was strongly associated with fasting glucose (*p* = 3.7E-08). Intronic and 3′UTR variants in the *APOA5-ZNF259* region on chromosome 11 were associated with triglycerides (*p* = 2.5-4.8E-08). An intronic variant in *PCSK2* on chromosome 20 was associated with total antioxidants (*p* = 7.6E-08). Variants in the flanking 3′UTR regions of *RNASE1* on chromosome 14 and *ASS1P11* on chromosome 7 were associated with 24-h urinary nitrogen excretion and creatinine excretion, respectively (*p* = 8.2-8.4E-08). An intronic SNP in *GCH1* on chromosome 14 was associated with 24-h urinary dopamine: creatinine ratio (*p* = 6.3E-08). A nonsynonymous SNP (rs3733402; S143T (NP_000883.2)) in *KLKB1* on chromosome 4 was identified for serum free IGF-1. An intronic SNP in *MPRIP* on chromosome 17 was associated with serum IGFBP-3 (*p* = 7.2E-08). A block of twenty-three SNPs in the flanking 5′UTR region of *XPA/FOXE1 (TTF-2)* on chromosome 9 were highly associated with serum TSH (*p* = 5.5E-08 to 1.0E-09) and found to be in strong linkage disequilibrium with one another (*R* = 0.91−1.00).

**Table 2:** Measured genotype analysis for endometabolic traits.

SNP	Chr	Coordinate(GB 36.2)	Location(GB 36.2)	SNP (GB36.2)	Trait	MGA p-value	Effect Size	Minor Allele	Minor Allele Frequency	Gene Symbol	Gene Name
rs3733402	4	187395028	coding-nonsym	[A/G]	IGF-1 free (ng/mL)	9.01E-**08	0.037	G	0.337	*KLKB1*	kallikrein B, plasma (Fletcher factor) 1
rs11974269	7	21114203	flanking_3UTR	[T/G]	Urinary creatinine(mmol/d)	8.38E-08**	0.051	C	0.128	*ASS1P11*	argininosuccinate synthetase 1 pseudogene 11
rs7030241	9	99590196	flanking_5UTR	[T/A]	TSH (µIU/mL)	1.03E-09	0.038	T	0.253	*XPA/FOXE1*	xeroderma pigmentosum, complementation group A/forkhead box E1 (thyroid transcription factor 2)
rs10830963	11	92348358	intron	[G/C]	Glucose (mg/dL)	3.72E-08*	0.048	G	0.205	*MTNR1B*	melatonin receptor 1B
rs3741298	11	116162771	intron	[A/G]	Triglycerides(mg/dL)	2.47E-08*	0.043	G	0.480	*ZNF259*	zinc finger protein 259
rs2266788	11	116165896	3UTR	[A/G]	Triglycerides(mg/dL)	4.82E-08	0.042	G	0.163	*APOA5*	apolipoprotein A-V
rs10131141	14	20331573	flanking_3UTR	[A/G]	Urinary nitrogen (g/d)	8.19E-08**	0.040	G	0.280	*RNASE1*	ribonuclease, RNase A family, 1 (pancreatic)
rs3783637	14	54417868	intron	[T/C]	Urinary freedopamine:creatinine	6.29E-08**	0.051	A	0.121	*GCH1*	GTP cyclohydrolase 1
rs61744862	17	17008907	intron	[A/G]	IGFBP-3 (ng/mL)	7.24E-08**	0.037	A	0.041	*MPRIP*	myosin phosphatase Rho interacting protein
rs6044834	20	17384473	intron	[A/C]	Total antioxidants(mM)	7.60E-08	0.036	C	0.069	*PCSK2*	proprotein convertase subtilisin/kexin type 2

Abbreviations: SNP, single nucleotide polymorphism; chr, chromosome; MGA, measured genotype analysis; nonsyn, nonsynonomous.

IGF-1, insulin-like growth factor-1; TSH, thyroid stimulating hormone; UTR, untranslated region; IGFBP3, binding protein 3.

* Significant according to the widely used significance threshold p value of <5×10^−8^.

** Significant according to the our population-specific significance threshold p value of <1.01×10^−7^.

### Inflammation Markers

Genome-wide significant variants were identified for inflammation markers (**Table 3**). Highly significant associations for a nonsynonymous SNP (rs12075; G42D (NP_002027.2) (*p* = 1.3E-21) and an intronic SNP (*p* = 3.6E-13) in *DARC* on chromosome 1 were identified for MCP-1. Coding variants in *GREB1* on chromosome 2 (*p* = 6.5E-08) and *DFNB31* on chromosome 9 (*p* = 2.0E-08) were also identified for MCP-1. A variants in the 3′UTR for *CCR3* was highly associated with MCP1, as well as an intronic SNP in *RASGEF1A* (*p* = 4.6-9.6E-08). A variant in the intronic region of *ABO* was strongly associated with IL-6 (*p* = 2.0E-08).

**Table 3:** Measured genotype analysis for inflammation markers.

SNP	Chr	Coordinate (GB 36.2)	Location(GB 36.2)	SNP (GB 36.2)	Trait	MGAp-value	Effect Size	Minor Allele	Minor Allele Frequency	Gene Symbol	Gene Name
rs863002	1	157441544	intron	[A/G]	MCP-1 (pg/mL)	3.59E-13*	0.062	A	0.244	*DARC*	Duffy blood group, chemokine receptor
rs12075	1	157441978	coding-nonsyn	[A/G]	MCP-1 (pg/mL)	1.31E-21*	0.103	A	0.436	*DARC*	Duffy blood group, chemokine receptor
rs73175262	2	11675882	coding	[A/G]	MCP-1 (pg/mL)	6.46E-08**	0.049	A	0.053	*GREB1*	growth regulation by estrogen in breast cancer 1
rs7645716	3	46311785	flanking_3UTR	[A/G]	MCP-1 (pg/mL)	9.56E-08**	0.041	A	0.381	*CCR3*	chemokine (C-C motif) receptor 3
rs79509430	9	116280727	coding	[T/C]	MCP-1 (pg/mL)	2.03E-08*	0.056	A	0.068	*DFNB31*	deafness, autosomal recessive 31
rs657152	9	135129086	intron	[A/C]	IL-6 (pg/mL)	2.03E-08*	0.041	A	0.254	*ABO*	ABO blood group (transferase A, alpha 1-3-N-acetylgalactosaminyltransferase; transferase B, alpha 1-3-galactosyltransferase)
rs28461806	10	43075760	intron	[A/G]	MCP-1 (pg/mL)	4.58E-08*	0.053	G	0.049	*RASGEF1A*	RasGEF domain family, member 1A

Abbreviations: SNP, single nucleotide polymorphism; chr, chromosome; MGA, measured genotype analysis; nonsyn, nonsynonomous;

MCP-1, monocyte chemotactic protein-1; UTR, untranslated region; IL-6, interluekin-6.

* Significant according to the widely used significance threshold p value of <5×10^−8^.

** Significant according to the our population-specific significance threshold p value of <1.01×10^−7^.

### Diet, Energy Expenditure, Substrate Utilization and Physical Activity

Genome-wide significant variants were associated with energy intake, energy expenditure and substrate utilization, and physical activity (**Table 4**). An intronic variant in *TMEM229B* on chromosome 14 was associated with *ad libitum* energy intake at dinner (*p* = 5.1E-08). An intronic variant in *ARHGAP11A* on chromosome 15 was associated with sleep duration (*p* = 5.0E-08). A SNP in the intronic region of *C21orf34* was detected for respiratory quotient (RQ) during sleep (*p* = 5.3E-08). A variant was identified for accelerometer-measured light activity in the 5′UTR region of *RPL7P3* on chromosome 9 (*p* = 7.5E-08) and sedentary-light activity in 3′UTR of *CTCFL* on chromosome 20 (*p* = 3.6E-08). In addition, adjusting for body weight, highly significant variants were identified for total energy expenditure (*p* = 2.7E-08) for *MATK* on chromosome 19 and sleeping energy expenditure (*p* = 6.0E-08) for *CHRNA3* on chromosome 15.

**Table 4:** Measured genotype analysis for diet, energy expenditure, substrate utilization and physical activity.

SNP	Chr	Coordinate(GB 36.2)	Location(GB 36.2)	SNP (GB 36.2)	Trait	MGAp-value	Effect Size	Minor Allele	Minor Allele Frequency	Gene Symbol	Gene Name
rs16933006	9	15325914	flanking_5UTR	[A/C]	Light activity (min/d)	7.49E-08**	0.035	C	0.110	*RPL7P3*	ribosomal protein L7 pseudogene 33
rs17104363	14	67009236	intron	[T/C]	Dinner intake, adjEER (kcal)	5.13E-08**	0.044	G	0.050	*TMEM229B*	transmembrane protein 229B
rs8037818	15	30714768	intron	[A/G]	Sleep duration (min/d)	4.95E-08*	0.042	G	0.181	*ARHGAP11A*	Rho GTPase activating protein 11A
rs8040868	15	76698236	coding	[A/G]	Sleep energyexpenditure adjweight (kcal/d)	5.95E-08**	0.048	G	0.239	*CHRNA3*	cholinergic receptor, nicotinic, alpha 3 (neuronal)
rs12104221	19	3748100	intron	[A/G]	Total energyexpenditure adjweight (kcal/d)	2.65E-08*	0.054	A	0.381	*MATK*	megakaryocyte-associated tyrosine kinase
rs6025590	20	55503911	flanking_3UTR	[T/C]	Sedentary&lightactivity (min/d)	3.61E-08*	0.039	A	0.308	*CTCFL*	CCCTC-binding factor (zinc finger protein)-like
rs2823615	21	16405004	intron	[T/A]	Sleep RQ	5.28E-08**	0.044	A	0.178	*C21orf34*	long intergenic non-protein coding RNA 478 (LINC00478)

Abbreviations: SNP, single nucleotide polymorphism; chr, chromosome; MGA, measured genotype analysis;

EER, estimated energy expenditure; UTR, untranslated region; RQ, respiratory quotient.

* Significant according to the widely used significance threshold p value of <5×10^-8^.

** Significant according to the our population-specific significance threshold p value of <1.01×10^−7^.

## Discussion

Extensive phenotyping and high-density SNP genotyping enabled localization of novel genetic loci associated with the pathophysiology of obesity in Hispanic children. Our unprecedented phenotypes represent not only adiposity, but also biological processes underlying the development of childhood obesity. The number of genome-wide significant genetic variants detected substantiates our analytical strategy and statistical power to identify variants.

Genome-wide significant and suggestive genetic variants were associated with anthropometric indices, body composition and growth rate. The common nonsynonymous SNP rs1056513 in *INADL* accounted for 3% of the variance in body weight and body composition, and is highly conserved across mammalian species. *INADL* encodes a PDZ domain-containing protein thought to play a role in tight junctions and adipocyte differentiation [28]. Weight z-score change observed over one-year was associated with an intronic variant in *COL4A1,* which encodes a basement membrane collagen. Height change was associated with a 5′UTR variant in*TSEN34,* which is involved in tRNA splicing, a fundamental process required for cell growth and division [29].

Fasting serum glucose was associated with an intronic variant (MAF = 0.20; effect size 4.8%) in *MTNR1B* which encodes one of the melatonin receptors expressed in the retina and brain. Corroborating our findings, this variant (rs10830963) has been strongly associated with fasting glucose levels in adults [30], [31] and children [32]–[35]. Melatonin, the ligand to MTNR1B, has an inhibitory effect on insulin secretion resulting in elevated fasting glucose.

Fasting serum triglycerides were associated with variants in the intron and 3′UTRs within the *APOA5-ZNF259* region. APOA5 is an important determinant of circulating triglyceride levels [36]. SNPs detected in our GWAS have been associated with triglycerides in other populations [37]. In the Kosrae population, triglyceride levels were associated with seven SNPs near *APOC3/A5* [38]. Variants in the *APOA5-ZNF259* region (including rs3741298) were associated with HDL-C and ApoA-1 response to therapy with statins and fenofibric acid in patients with dyslipidemia [39].

Association with total antioxidants was shown for a variant in *PCSK2,* a proprotein convertase that is involved in proteolytic processing of neuropeptide and hormone precursors. *PCSK2* is highly expressed in the islets of Langerhans, where it plays a role in the conversion of proinsulin to insulin.

A LD block of 23 SNPs in the *XPA/FOXE1* (*TTF-2)* region was highly associated with fasting serum TSH (MAF = 0.25-0.29, effect size = 2.9-3.8%). The association is likely attributed to *FOXE1* rather than *XPA* which is involved in DNA excision repair [40] and the skin disease xeroderma pigmentosum [41]. *FOXE1* or *thyroid transcription factor 2* (*TTF-2*) belongs to the ‘forkhead’ gene family and is involved in promoting the migration process or in repressing differentiation of the thyroid follicular cells until migration has occurred [42] and has been associated with thyroid cancer [43]–[46]. In the Kosrae population, plasma TSH levels were strongly associated with 10 SNPs in a region encompassing *TTF-2* on chromosome 9 [38]. In a cohort of Caucasian adults, genetic variation in *FOXE1(TTF-2)* showed significant effects on free T4 levels and borderline effects on serum TSH [47]. Three of the SNPs (rs925488, rs1877431, rs1588635) detected in the VIVA cohort was also reported by Lowe et.al (38); there was no overlap with Medici et al (43). Also, three of the SNPs (rs2805809, rs2668804, rs2808693) reported by Lowe et al. (38) was in high linkage disequilibrium (1.00) with rs2805771, rs2808699, rs7875482 detected in the VIVA cohort. This is a region of high LD, located in the 5′UTR-region of the gene which has been shown to influence transcriptional regulation of *FOXE1(TTF-2*). The functional variant is likely to be situated at this locus, but its exact localization remains to be elucidated in future studies, involving in-depth resequencing.

A unifying role of inflammation in chronic diseases including cardiovascular disease, diabetes, hypertension and obesity is emerging. In the VIVA GWAS, variants in six genes were associated with proinflammatory markers. Our findings support a major role of Duffy antigen receptor for chemokines (*DARC*) in the regulation of the circulating levels of the cysteine-cysteine (CC) chemokine, MCP-1 (effect size = ∼10%). In circulation, MCP-1 is bound to erythrocyte DARC that acts as a chemokine receptor/reservoir of proinflammatory cytokines [48]. Our strongest association for MCP-1 was with a nonsynonymous, highly conservedSNP in *DARC* (rs12075; MAF = 0.44) which replicated results from the Framingham Heart Study GWAS in Caucasian adults [49]. MCP-1 was also associated with SNPs in *GREB1, DFNB31, RASGEF1A*, and *CCR3.* Huber et al. found increased expression of *CCR3* in subcutaneous and visceral adipose tissue in obese patients compared to lean controls [50]. Studies by Schnabel et al [49] and Naitza et al. [51] found suggestive associations of serum MCP-1 with *CCR2* which is also a MCP-1 receptor and is in the same region of chromosome 3 as *CCR3,* and referred to as the *CCR2/CCR3* cytokine receptor gene cluster.

Fasting serum IL-6 levels were associated with variants in the *ABO* gene that determines blood group. ABO blood group has been found to be associated with a number of biomarkers such as von Willebrand factor levels, Factor VIII levels, thrombomodulin, TNF-α and ICAM-1 [52], [53], and in our case IL-6. The mechanism by which the A and B alleles affect these biomarkers is uncertain. In Caucasians with and without type 1 diabetes, a variant rs579459 near the *ABO* blood group gene accounted for 19% of the variance in E-selectin levels [52]; in our GWAS, this same variant was associated with IL-6 levels (*p* = 1.7E-07; **Table S2**).

Total energy expenditure, adjusted for body weight, was significantly associated with rs12104221 in *MATK* which encodes a protein-tyrosine kinase involved in signal transduction pathways [54]. Sleeping energy expenditure, adjusted for body weight, was associated with rs8040868 in *CHRNA3* (cholinergic receptor, neuronal nicotinic, alpha polypeptide 3), a member of a superfamily of ligand-gated ion channels that mediate fast signal transmission at synapses. After binding to acetylcholine, the receptor responds by opening ion-conducting channels across the plasma membrane, suggesting a plausible role of the coding variant (rs8040868) in energy metabolism [55]. Acetylcholine receptors activate proopiomelanocortin neurons that in turn activate melanocortin-4 receptors that are involved in the regulation of energy intake and expenditure [56], [57].

Sleep duration was associated with an intronic SNP in *ARHGAP11A* (rho GTPase activating protein 11A) that encodes a 1,023-amino acid protein that has a rhoGAP domain and tyrosine phosphorylation site. Evidence is emerging that obesity affects sleep, and that sleep patterns and disorders may have an effect on weight [58]. Although the mechanism is unclear, sleep disturbances are characteristic of Prader Willi Syndrome, caused by a deletion in 15q11-q13 that encompasses *ARHGAP11A* [59].

Sedentary-light physical activity was associated with a variant in *CTCFL,* an 11-zinc-finger factor involved in gene regulation. CTCFL forms methylation-sensitive insulators that regulate X-chromosome inactivation which may play a role in epigenetic regulation [60].

Variants in the 11 genes known to cause extreme early-onset obesity also may contribute to milder forms of obesity [61], [62]. None of the genotyped variants in genes for monogenic obesity reached genome-wide significance in our GWAS, although several variants in *CRHR1, CRHR2, MCHR1, MC3R, MC4R* and *POMC* were nominally associated. Similarly, variants in or nearby the susceptibility genes for obesity did not attain genome-wide significance but several - *CHST8, KCTD15, MTCH2, SFRS10, SH2B1* and *TMEM18 -* were nominally associated with obesity-related traits, consistent with GWAS in children of European-American [63] or European ancestry [9]. The lack of genome-wide significant findings for monogenic causes of obesity or susceptibility genes may be a function of our sample size and statistical power, or the presence of rare variants in the Hispanic population not represented in the Illumina platform.

The VIVA LA FAMILIA Study is unique in its consideration of a pediatric Hispanic population. We believe that our extensive phenotyping and genotyping enabled localization of novel genetic loci associated with obesity in Hispanic children, despite our relatively small sample size. Our phenotypes represent not only adiposity, but also biological processes underlying the development and consequences of childhood obesity. We applied a standard and stringent approach to our measured genotype analysis, but do agree replication is desirable. We believe we have identified genes that warrant further investigation.

In conclusion, unprecedented in-depth phenotyping and high-density SNP genotyping enabled the localization of novel genetic loci associated with the pathophysiology of obesity in Hispanic children. Identified genome-wide significant loci: 1) corroborated genes implicated in other studies (*MTNR1B, ZNF259/APOA5, XPA/FOXE1 (TTF-2), DARC, CCR3, ABO*); 2) localized novel genes in plausible biological pathways (*PCSK2, ARHGAP11A, CHRNA3*); and 3) revealed novel genes with unknown function in obesity pathogenesis (*MATK*, *COL4A1*). As with other GWAS, the variants identified are likely not the actual causal variants but rather markers for genomic regions or loci in which the causal variants lie. Characterization of the underlying functional genetic variants contributing to this serious public health problem in Hispanic children will involve additional study.

## Supporting Information

Figure S1 **GWAS Manhattan plots are displayed for three phenotypes: total energy expenditure, adjusted for body weight, measured by 24-h room calorimetry; fasting serum thyroid stimulating hormone; and 1-y change in weight z-score.** The genomic coordinates are shown along the X-axis, and the negative logarithm of the association p-value for each SNP on the Y-axis.
(TIFF)

Table S1 **Description of the phenotypes used in the genome-wide association study.**
(DOCX)

Table S2 **Suggestive and genome-wide significant genetic variants identified by measured genotype analysis.**
(DOCX)
