# Supplementary figures and images for: Novel Genetic Loci Identified for the Pathophysiology of Childhood Obesity in the Hispanic Population
Source: PLoS One. 2012 Dec 14;7(12):e51954. doi: 10.1371/journal.pone.0051954 (PMC3522587; doi:10.1371/journal.pone.0051954)

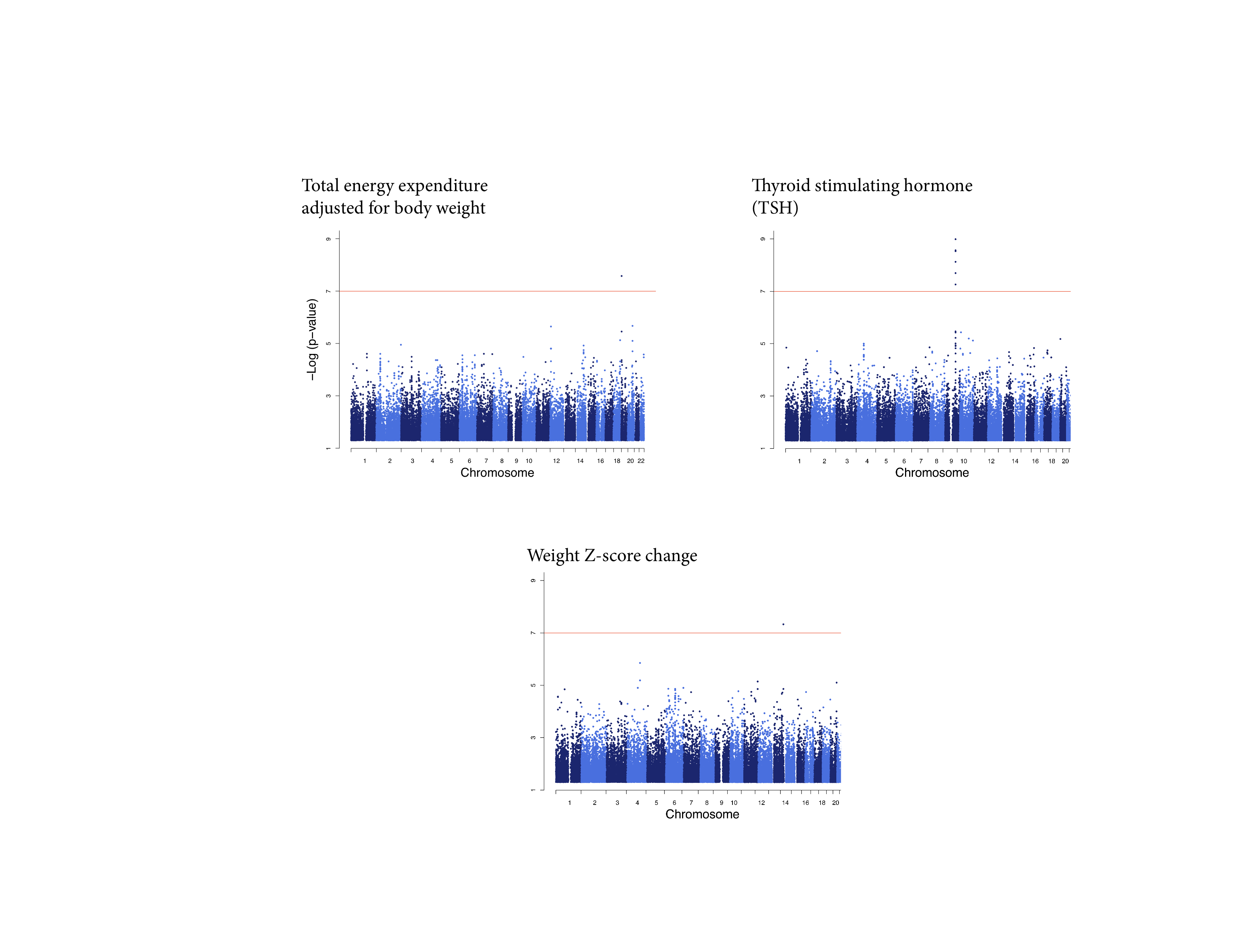

Supplement: Figure S1 — GWAS Manhattan plots are displayed for three phenotypes: total energy expenditure, adjusted for body weight, measured by 24-h room calorimetry; fasting serum thyroid stimulating hormone; and 1-y change in weight z-score. The genomic coordinates are shown along the X-axis, and the negative logarithm of the association p-value for each SNP on the Y-axis. (TIFF) [file pone.0051954.s001.tiff]
