# Supplementary material for: Novel Genetic Loci Identified for the Pathophysiology of Childhood Obesity in the Hispanic Population
Source: PLoS One. 2012 Dec 14;7(12):e51954. doi: 10.1371/journal.pone.0051954 (PMC3522587; doi:10.1371/journal.pone.0051954)
Supplement: Table S1 — Description of the phenotypes used in the genome-wide association study. (DOCX) [file pone.0051954.s002.docx]

| **Table S1.** Description of the phenotypes used in the genome-wide association study | |
| --- | --- |
| Trait | Description |
| Adiponectin (ng/mL) | fasting serum adiponectin |
| ALT (U/L) | fasting serum alanine aminotransferase |
| ALT/AST | fasting serum alanine aminotransferase: aspartate aminotransferase ratio |
| Amylin (pM) | fasting plasma amylin |
| Arm span (cm) | arm span |
| AST (U/L) | fasting serum aspartate aminotransferase |
| AST/ALT | fasting serum aspartate aminotransferase: alanine aminotransferase ratio |
| Bike energy expenditure (kcal/min) | energy expended during 15-min on stationary ergometer |
| Birth weight (kg) | birth weight from birth records |
| BMI (kg/m2) | body mass index |
| BMI z-score (SD) | body mass index z-score (CDC 2000) |
| BMI z-score change (SD/y) | 1-y change in body mass index z-score (CDC 2000) |
| BMR (kcal/d) | basal metabolic rate |
| BMR RQ | respiratory quotient during basal metabolic rate measurement |
| Bone mineral content (kg) | bone mineral content measured by dual energy x-ray absorptiometry |
| Bone mineral density (g/cm2) | bone mineral density measured by dual energy x-ray absorptiometry |
| Calorimeter activity (counts/d) | Physical activity measured during 24-h room calorimetry |
| Cortisol (ng/mL) | fasting serum cortisol |
| C-peptide (ng/mL) | fasting serum C-peptide |
| CRP (ng/mL) | fasting serum C-reactive protein |
| Cystathionine (µmol/L) | fasting plasma cystathionine |
| Diastolic blood pressure (mmHg) | diastolic blood pressure |
| Diet carbohydrate (%energy) | diet carbohydrate, as a percent of total daily energy intake |
| Diet carbohydrate (g/d) | diet carbohydrate, as total grams per day |
| Diet fat (%energy) | diet fat, as a percent of total daily energy intake |
| Diet fat (g/d) | diet fat, as total grams per day |
| Diet protein (%energy) | diet protein, as a percent of total daily energy intake |
| Diet protein (g/d) | diet protein, as total grams per day |
| Dinner intake (kcal) | observed dinner intake, as total calories |
| Dinner intake, adj EER (kcal) | observed dinner intake, as total calories, adjusted for estimated energy requirement |
| Dinner intake, adj TEE (kcal) | observed dinner intake, as total calories, adjusted for 24-h total energy expenditure |
| Energy balance (kcal/d) | observed energy balance, as calories per day, during 24-h room calorimetry |
| Energy intake (kcal/d) | energy intake, as calories per day, by 24-h diet recall |
| Energy storage (kcal/d) | energy storage, as calories day, estimated from body composition changes |
| Eotaxin (pg/mL) | fasting serum eotaxin |
| Estradiol (pg/mL) | fasting serum estradiol |
| Fat free mass (kg) | fat free mass, measured by dual energy x-ray absorptiometry |
| Fat free mass (%) | fat free mass, as a percent of body weight |
| Fat free mass change (kg/y) | 1-y change in fat free mass |
| Fat mass (kg) | fat mass, measured by dual energy x-ray absorptiometry |
| Fat mass (%) | fat mass, as a percent of body weight |
| Fat mass change (kg/y) | 1-y change in fat mass |
| Fat mass deposition (kcal/d) | fat mass deposition, as calories per day |
| Fat oxidation (%NPEE) | fat oxidation, as a percent of nonprotein energy expenditure, measured by 24-h calorimetry |
| Folate (nmol/L) | fasting plasma folate |
| Free T3 (pg/mL) | fasting serum free triiodothyronine |
| Free T4 (ng/dL) | fasting serum free thyroxine |
| Gestational age (wk) | gestational age from birth records |
| Ghrelin (pg/100 µL) | fasting plasma ghrelin |
| Glucose (mg/dL) | fasting serum glucose |
| HDL (mg/dL) | fasting serum high-density lipoprotein cholesterol |
| Head circumference (cm) | head circumference |
| Height (cm) | height |
| Height change (cm/y) | 1-y change in height |
| Height z-score (SD) | height z-score (CDC 2000) |
| Height z-score change (SD/y) | 1-y change in height z-score (CDC 2000) |
| Hip circumference (cm) | hip circumference |
| Hip circumference change (cm/y) | 1-y change in hip circumference |
| HOMA-IR | fasting serum homeostatic model assessment-insulin resistance |
| HRmax (bpm) | maximum heart rate during treadmill fitness test |
| IGF-1 bound (ng/mL) | fasting serum insulin-like growth factor-1 bound |
| IGF-1 free (ng/mL) | fasting serum insulin-like growth factor-1 free |
| IGFBP-1 (ng/mL) | fasting serum insulin-like growth factor binding protein-1 |
| IGFBP-3 (ng/mL) | fasting serum insulin-like growth factor binding protein-3 |
| IL-6 (pg/mL) | fasting serum interluekin-6 |
| Insulin (µU/mL) | fasting serum insulin |
| LDL (mg/dL) | fasting serum low-density lipoprotein cholesterol |
| Lean body mass (kg) | lean body mass |
| Leptin (ng/mL) | fasting serum leptin |
| Light activity (%awake time) | light activity, as a percent awake time, measured by accelerometry |
| Light activity (min/d) | light activity, as minutes per day, measured by accelerometry |
| MCP-1 (pg/mL) | fasting serum monocyte chemotactic protein-1 |
| Methionine (µmol/L) | fasting plasma methionine |
| Moderate activity (%awake time) | moderate activity, as a percent awake time, measured by accelerometry |
| Moderate activity (min/d) | moderate activity, as minutes per day, measured by accelerometry |
| Moderate & vigorous activity (min/d) | moderate & vigorous activity, as minutes per day, measured by accelerometry |
| NEFA (mmol/L) | fasting serum nonesterified fatty acids |
| Protein (kg/y) | 1-y change in body protein |
| Protein deposition (kcal/d) | protein deposition, as calories per day |
| QUICKl | fasting serum quantitative insulin sensitivity check index |
| RANTES (pg/mL) | fasting serum regulated upon activation, normal T-cell expressed and secreted |
| RQmax | maximum respiratory quotient during treadmill fitness test |
| Sedentary activity (%awake time) | sedentary activity, as a percent awake time, measured by accelerometry |
| Sedentary activity (min/d) | sedentary activity, as minutes per day, measured by accelerometry |
| Sedentary & light activity (min/d) | sedentary & light activity, as minutes per day, measured by accelerometry |
| sICAM-1 (pg/mL) | fasting serum soluble intercellular adhesion molecule-1 |
| Sitting height (cm) | sitting height |
| Sleep duration (min/d) | sleep duration |
| Sleep energy expenditure (kcal/d) | sleeping energy expenditure, measured by 24-h room calorimetry |
| Sleep energy expenditure, adj weight (kcal/d) | sleeping energy expenditure, adjusted for body weight |
| Sleep RQ | respiratory quotient during sleep |
| Snack intake (kcal) | observed snack intake, as total calories |
| Snack intake, adj TEE (kcal) | observed snack intake, as total calories, adjusted for 24-h total energy expenditure |
| Systolic blood pressure (mmHg) | systolic blood pressure |
| TBF-ß1 (pg/mL) | fasting serum transforming growth factor-beta 1 |
| Testosterone (ng/mL) | fasting serum testosterone |
| TG/HDLC (mmol/L) | fasting serum triglycerides/high-density lipoprotein cholesterol |
| TNF-α (pg/mL) | fasting serum tumor necrosis factor-alpha |
| Total antioxidants (mM) | fasting serum total antioxidants |
| Total cholesterol (mg/dL) | fasting serum total cholesterol |
| Total cysteine (µmol/L) | fasting plasma total cysteine |
| Total energy expenditure (kcal/d) | total energy expenditure, measured by 24-h room calorimetry |
| Total energy expenditure, adj for weight (kcal/d) | total energy expenditure, adjusted for body weight, measured by 24-h room calorimetry |
| Total energy expenditure RQ | respiratory quotient, measured by 24-h room calorimetry |
| Total glutathione (µmol/L) | fasting plasma glutathione |
| Total homocysteine (µmol/L) | fasting plasma total homocysteine |
| Total T3 (ng/dL) | fasting serum triiodothyronine |
| Total T4 (µg/dL) | fasting serum thyroxine |
| Triglycerides (mg/dL) | fasting serum triglycerides |
| Trunk fat mass (kg) | trunk fat mass, measured by dual energy x-ray absorptiometry |
| TSH (µIU/mL) | fasting serum thyroid stimulating hormone |
| Urinary creatinine (mmol/d) | urinary creatinine excretion per day |
| Urinary free dopamine (nmol/d) | urinary free dopamine excretion per day |
| Urinary free dopamine: creatinine | urinary free dopamine: creatinine ratio |
| Urinary free epinephrine (nmol/d) | urinary free epinephrine excretion per day |
| Urinary free norepinephrine (nmol/d) | urinary free norepinephrine excretion per day |
| Urinary free norepinephrine: creatinine | urinary free norepinephrine: creatinine ratio |
| Urinary nitrogen (g/d) | urinary nitrogen excretion per day |
| Vigorous activity (%awake time) | vigorous activity, as a percent awake time, measured by accelerometry |
| Vigorous activity (min/d) | vigorous activity, as minutes per day, measured by accelerometry |
| Vitamin B12 (pmol/L) | fasting plasma vitamin B12 |
| VO2max (mL/min) | maximum oxygen consumption during treadmill fitness test |
| Waist circumference (cm) | waist circumference |
| Waist circumference change (cm/y) | 1-y change in waist circumference |
| Waist: height | waist circumference: height ratio |
| Weight (kg) | weight |
| Weight change (kg/y) | 1-y change in weight |
| Weight z-score (SD) | weight z-score (CDC 2000) |
| Weight z-score change (SD/y) | 1-y change in weight z-score (CDC 2000) |
