# Supplementary material for: Novel Genetic Loci Identified for the Pathophysiology of Childhood Obesity in the Hispanic Population
Source: PLoS One. 2012 Dec 14;7(12):e51954. doi: 10.1371/journal.pone.0051954 (PMC3522587; doi:10.1371/journal.pone.0051954)
Supplement: Table S2 — Suggestive and genome-wide significant genetic variants identified by measured genotype analysis. (DOCX) [file pone.0051954.s003.docx]

| **Table S2. Suggestive and genome-wide significant genetic variants identified by measured genotype analysis** | | | | | | | | | | | | | |
| --- | --- | --- | --- | --- | --- | --- | --- | --- | --- | --- | --- | --- | --- |
| **SNP** | **Chr** | **Coordinate (GB 36.2)** | **Location (GB 36.2)** | **Coding Status (GB 36.2)** | **AA Change (GB 36.2)** | **SNP (GB 36.2)** | **Trait** | **MGA p-value** | **Effect Size** | **Minor Allele** | **Minor Allele Frequency** | **Gene Symbol** | **Gene Name** |
| rs12073504 | 1 | 2442839 | intron |  |  | [G/C] | IGFBP-3 (ng/mL) | 4.91E-06 | 0.027 | G | 0.033 | *PANK4* | pantothenate kinase 4 |
| rs76597070 | 1 | 3659216 | flanking_5UTR |  |  | [A/G] | Calorimeter activity (counts/d) | 2.52E-06 | 0.034 | A | 0.005 | *CCDC27* | coiled-coil domain containing 27 |
| rs75411 | 1 | 4325274 | flanking_5UTR |  |  | [T/C] | Urinary free dopamine: creatinine | 3.63E-06 | 0.027 | A | 0.096 | *LOC644357* | eukaryotic translation elongation factor 1 delta (guanine nucleotide exchange protein) pseudogene |
| rs589870 | 1 | 4340609 | flanking_5UTR |  |  | [T/C] | Urinary free dopamine: creatinine | 6.09E-06 | 0.025 | A | 0.094 | *LOC644357* | eukaryotic translation elongation factor 1 delta (guanine nucleotide exchange protein) pseudogene |
| rs239339 | 1 | 4344399 | flanking_5UTR |  |  | [T/G] | Urinary free dopamine: creatinine | 2.81E-06 | 0.027 | A | 0.095 | *LOC644357* | eukaryotic translation elongation factor 1 delta (guanine nucleotide exchange protein) pseudogene |
| rs2489260 | 1 | 12663827 | flanking_3UTR |  |  | [A/G] | Sitting height (cm) | 8.21E-06 | 0.039 | A | 0.145 | *AADACL4* | arylacetamide deacetylase-like 4 |
| rs59905655 | 1 | 15943597 | coding |  |  | [T/C] | IGFBP-3 (ng/mL) | 1.58E-06 | 0.030 | A | 0.043 | *TMEM82* | transmembrane protein 82 |
| rs6693036 | 1 | 17792404 | intron |  |  | [A/G] | Triglycerides (mg/dL) | 2.58E-06 | 0.038 | G | 0.048 | *ARHGEF10L* | Rho guanine nucleotide exchange factor (GEF) 10-like |
| rs1339934 | 1 | 18109046 | flanking_3UTR |  |  | [A/G] | Ghrelin (pg/100 µL) | 6.78E-06 | 0.029 | A | 0.494 | *ACTL8* | actin-like 8 |
| rs6686929 | 1 | 18117294 | flanking_3UTR |  |  | [T/C] | Ghrelin (pg/100 µL) | 3.33E-06 | 0.031 | G | 0.486 | *ACTL8* | actin-like 8 |
| rs623784 | 1 | 20325021 | flanking_5UTR |  |  | [T/C] | BMI z-score change (SD/y) | 8.67E-06 | 0.031 | G | 0.275 | *PLA2G2D* | phospholipase A2, group IID |
| rs12722987 | 1 | 20327807 | flanking_5UTR |  |  | [A/C] | BMI z-score change (SD/y) | 6.57E-06 | 0.031 | A | 0.178 | *PLA2G2D* | phospholipase A2, group IID |
| rs213032 | 1 | 21527322 | flanking_5UTR |  |  | [T/C] | Sleep duration (min/d) | 5.65E-06 | 0.028 | G | 0.281 | *ECE1* | endothelin converting enzyme 1 |
| rs17360053 | 1 | 22372117 | flanking_5UTR |  |  | [A/G] | ALT/AST | 8.84E-06 | 0.028 | G | 0.190 | *WNT4* | wingless-type MMTV integration site family, member |
| rs17338135 | 1 | 24159484 | intron |  |  | [A/G] | Calorimeter activity (counts/d) | 9.49E-06 | 0.033 | G | 0.375 | *PNRC2* | proline-rich nuclear receptor coactivator 2 |
| rs6667686 | 1 | 24173436 | intron |  |  | [T/C] | Calorimeter activity (counts/d) | 2.43E-06 | 0.038 | G | 0.272 | *SRSF10* | serine/arginine-rich splicing factor 10 |
| rs10902723 | 1 | 26441129 | intron |  |  | [A/G] | TBF-ß1 (pg/mL) | 8.45E-06 | 0.007 | G | 0.411 | *CEP85/CCDC21* | centrosomal protein 85kDa/coiled-coil domain containing 21 |
| rs6598955 | 1 | 26492236 | intron |  |  | [A/G] | HOMA-IR | 3.48E-06 | 0.032 | A | 0.256 | *UBXN11* | UBX domain protein 11 |
| rs6598955 | 1 | 26492236 | intron |  |  | [A/G] | Insulin (µU/mL) | 7.42E-06 | 0.030 | A | 0.256 | *UBXN11* | UBX domain protein 11 |
| rs11247915 | 1 | 26523137 | flanking_3UTR |  |  | [T/G] | Calorimeter activity (counts/d) | 5.30E-06 | 0.034 | A | 0.233 | *AIM1L* | absent in melanoma 1-like |
| rs11587947 | 1 | 26591851 | flanking_5UTR |  |  | [A/G] | Adiponectin (ng/mL) | 9.69E-06 | 0.025 | A | 0.108 | *LIN28* | lin-28 homolog A (C. elegans) |
| rs685001 | 1 | 47797650 | flanking_3UTR |  |  | [T/G] | LDL (mg/dL) | 9.25E-06 | 0.029 | C | 0.362 | *FOXD2* | forkhead box D2 |
| rs6588505 | 1 | 48027369 | intron |  |  | [T/C] | Eotaxin (pg/mL) | 9.36E-06 | 0.028 | A | 0.322 | *LOC388630* | UPF0632 protein A |
| rs12092053 | 1 | 50379616 | intron |  |  | [T/G] | IGFBP-1 (ng/mL) | 6.09E-06 | 0.023 | C | 0.004 | *ELAVL4* | ELAV (embryonic lethal, abnormal vision, Drosophila)-like 4 (Hu antigen D) |
| rs6662617 | 1 | 57042109 | intron |  |  | [T/C] | Diet protein (%energy) | 8.49E-06 | 0.024 | G | 0.271 | *C1orf168* | chromosome 1 open reading frame 168 |
| rs835367 | 1 | 59535056 | flanking_5UTR |  |  | [T/C] | HOMA-IR | 7.37E-06 | 0.028 | G | 0.371 | *FGGY* | FGGY carbohydrate kinase domain-containing protein |
| rs835367 | 1 | 59535056 | flanking_5UTR |  |  | [T/C] | Insulin (µU/mL) | 2.17E-06 | 0.031 | G | 0.371 | *FGGY* | FGGY carbohydrate kinase domain-containing protein |
| rs9787234 | 1 | 59569396 | intron |  |  | [A/G] | Insulin (µU/mL) | 7.79E-06 | 0.029 | A | 0.248 | *FGGY* | FGGY carbohydrate kinase domain-containing protein |
| rs7539697 | 1 | 59605350 | intron |  |  | [A/G] | Insulin (µU/mL) | 6.40E-06 | 0.027 | A | 0.342 | *FGGY* | FGGY carbohydrate kinase domain-containing protein |
| rs835435 | 1 | 59617059 | coding | SYNON | P60P(NP_060761.2) | [T/C] | Insulin (µU/mL) | 6.88E-06 | 0.028 | A | 0.393 | *FGGY* | FGGY carbohydrate kinase domain-containing protein |
| rs7534016 | 1 | 59625907 | intron |  |  | [T/C] | HOMA-IR | 5.26E-06 | 0.029 | G | 0.255 | *FGGY* | FGGY carbohydrate kinase domain-containing protein |
| rs7534016 | 1 | 59625907 | intron |  |  | [T/C] | Insulin (µU/mL) | 6.32E-06 | 0.029 | G | 0.255 | *FGGY* | FGGY carbohydrate kinase domain-containing protein |
| rs1056513 | 1 | 62152886 | coding | NONSYN | G1178S(NP_005790.2) | [A/G] | BMI (kg/m^2^) | 8.34E-06 | 0.021 | A | 0.495 | *INADL* | InaD-like (Drosophila) |
| rs1056513 | 1 | 62152886 | coding | NONSYN | G1178S(NP_005790.2) | [A/G] | Bone mineral content (kg) | 7.09E-06 | 0.023 | A | 0.495 | *INADL* | InaD-like (Drosophila) |
| rs1056513 | 1 | 62152886 | coding | NONSYN | G1178S(NP_005790.2) | [A/G] | Fat free mass (kg) | 2.80E-07 | 0.034 | A | 0.495 | *INADL* | InaD-like (Drosophila) |
| rs1056513 | 1 | 62152886 | coding | NONSYN | G1178S(NP_005790.2) | [A/G] | Fat mass (kg) | 1.59E-07 | 0.035 | A | 0.495 | *INADL* | InaD-like (Drosophila) |
| rs1056513 | 1 | 62152886 | coding | NONSYN | G1178S(NP_005790.2) | [A/G] | Fat mass (kg) | 7.49E-07 | 0.027 | A | 0.495 | *INADL* | InaD-like (Drosophila) |
| rs1056513 | 1 | 62152886 | coding | NONSYN | G1178S(NP_005790.2) | [A/G] | Hip circumference (cm) | 2.47E-06 | 0.022 | A | 0.495 | *INADL* | InaD-like (Drosophila) |
| rs1056513 | 1 | 62152886 | coding | NONSYN | G1178S(NP_005790.2) | [A/G] | Lean body mass (kg) | 3.19E-07 | 0.034 | A | 0.495 | *INADL* | InaD-like (Drosophila) |
| rs1056513 | 1 | 62152886 | coding | NONSYN | G1178S(NP_005790.2) | [A/G] | Leptin (ng/mL) | 7.21E-06 | 0.020 | A | 0.495 | *INADL* | InaD-like (Drosophila) |
| rs1056513 | 1 | 62152886 | coding | NONSYN | G1178S(NP_005790.2) | [A/G] | Sleep energy expenditure (kcal/d) | 6.52E-06 | 0.028 | A | 0.495 | *INADL* | InaD-like (Drosophila) |
| rs1056513 | 1 | 62152886 | coding | NONSYN | G1178S(NP_005790.2) | [A/G] | Total energy expenditure (kcal/d) | 5.54E-06 | 0.027 | A | 0.495 | *INADL* | InaD-like (Drosophila) |
| rs1056513 | 1 | 62152886 | coding | NONSYN | G1178S(NP_005790.2) | [A/G] | Trunk fat mass (kg) | 2.36E-07 | 0.035 | A | 0.495 | *INADL* | InaD-like (Drosophila) |
| rs1056513 | 1 | 62152886 | coding | NONSYN | G1178S(NP_005790.2) | [A/G] | Trunk fat mass (kg) | 4.78E-06 | 0.024 | A | 0.495 | *INADL* | InaD-like (Drosophila) |
| rs1056513 | 1 | 62152886 | coding | NONSYN | G1178S(NP_005790.2) | [A/G] | Weight (kg) | 1.18E-07 | 0.031 | A | 0.495 | *INADL* | InaD-like (Drosophila) |
| rs1023008 | 1 | 70177128 | intron |  |  | [T/A] | Light activity (%awake time) | 5.58E-06 | 0.028 | T | 0.481 | *LRRC7* | leucine rich repeat containing 7 |
| rs3000140 | 1 | 79773236 | flanking_3UTR |  |  | [T/G] | ALT/AST | 2.08E-06 | 0.029 | A | 0.027 | *ADH5P2* | alcohol dehydrogenase 5 (class III), chi polypeptide, pseudogene 2 |
| rs12085038 | 1 | 80232756 | flanking_5UTR |  |  | [A/G] | Sleep duration (min/d) | 6.33E-06 | 0.037 | A | 0.305 | *HMGB1P18* | high mobility group box 1 pseudogene 18 |
| rs10493631 | 1 | 80250468 | flanking_5UTR |  |  | [T/C] | Sleep duration (min/d) | 5.35E-06 | 0.036 | G | 0.306 | *HMGB1P18* | high mobility group box 1 pseudogene 18 |
| rs17436254 | 1 | 82534621 | flanking_3UTR |  |  | [T/C] | TBF-ß1 (pg/mL) | 8.68E-06 | 0.033 | G | 0.042 | *LPHN2* | latrophilin 2 |
| rs785584 | 1 | 82535288 | flanking_3UTR |  |  | [T/C] | TBF-ß1 (pg/mL) | 7.43E-06 | 0.031 | G | 0.071 | *LPHN2* | latrophilin 2 |
| rs1770678 | 1 | 82537640 | flanking_3UTR |  |  | [T/C] | TBF-ß1 (pg/mL) | 7.43E-06 | 0.031 | G | 0.070 | *LPHN2* | latrophilin 2 |
| rs17129289 | 1 | 84005336 | flanking_3UTR |  |  | [T/C] | Sleep duration (min/d) | 7.13E-06 | 0.024 | G | 0.011 | *TTLL7* | tubulin tyrosine ligase-like family, member 7 |
| rs10465746 | 1 | 84160220 | intron |  |  | [T/G] | AST (U/L) | 8.40E-06 | 0.026 | A | 0.447 | *TTLL7* | tubulin tyrosine ligase-like family, member 7 |
| rs316662 | 1 | 84559900 | intron |  |  | [A/C] | Hip circumference change (cm/y) | 4.46E-07 | 0.054 | A | 0.160 | *SAMD13* | sterile alpha motif domain containing 13 |
| rs315553 | 1 | 84565561 | intron |  |  | [A/C] | Hip circumference change (cm/y) | 4.09E-06 | 0.048 | C | 0.173 | *SAMD13* | sterile alpha motif domain containing 13 |
| rs315540 | 1 | 84589175 | flanking_3UTR |  |  | [A/C] | Hip circumference change (cm/y) | 6.96E-06 | 0.047 | A | 0.179 | *SAMD13* | sterile alpha motif domain containing 13 |
| rs315535 | 1 | 84592997 | flanking_3UTR |  |  | [A/G] | Hip circumference change (cm/y) | 7.38E-06 | 0.045 | G | 0.201 | *SAMD13* | sterile alpha motif domain containing 13 |
| rs17130643 | 1 | 89113996 | intron |  |  | [T/C] | sICAM-1 (pg/mL) | 3.48E-06 | 0.017 | A | 0.006 | *GTF2B* | General transcription factor IIB |
| rs34856868 | 1 | 92326871 | coding |  |  | [T/C] | Bone mineral density (g/cm^2^) | 2.21E-06 | 0.036 | A | 0.006 | *BTBD8* | BTB (POZ) domain containing 8 |
| rs10443196 | 1 | 99225875 | intron |  |  | [T/C] | LDL (mg/dL) | 8.66E-06 | 0.036 | G | 0.273 | *LPPR5* | lipid phosphate phosphatase-related protein type 5 |
| rs897603 | 1 | 99290933 | flanking_5UTR |  |  | [A/G] | Light activity (%awake time) | 5.16E-06 | 0.032 | A | 0.054 | *PAP2D* | lipid phosphate phosphatase-related protein type 5 |
| rs10747502 | 1 | 99296635 | flanking_5UTR |  |  | [T/C] | Light activity (%awake time) | 4.67E-07 | 0.041 | A | 0.061 | *PAP2D* | lipid phosphate phosphatase-related protein type 5 |
| rs7524694 | 1 | 103792219 | intron |  |  | [A/G] | Diet carbohydrate (g/d) | 6.49E-06 | 0.030 | G | 0.131 |  |  |
| rs3934285 | 1 | 104400464 | flanking_3UTR |  |  | [A/G] | Fat mass (%) | 1.59E-06 | 0.031 | A | 0.214 | *FTLP17* | ferritin, light polypeptide pseudogene 17 |
| rs3934285 | 1 | 104400464 | flanking_3UTR |  |  | [A/G] | Fat mass (kg) | 1.63E-06 | 0.029 | A | 0.214 | *FTLP17* | ferritin, light polypeptide pseudogene 17 |
| rs3934285 | 1 | 104400464 | flanking_3UTR |  |  | [A/G] | Trunk fat mass (kg) | 1.80E-06 | 0.030 | A | 0.214 | *FTLP17* | ferritin, light polypeptide pseudogene 17 |
| rs1043274 | 1 | 109843967 | flanking_3UTR |  |  | [T/C] | Total cholesterol (mg/dL) | 8.64E-06 | 0.030 | A | 0.026 | *CYB561D1* | cytochrome b-561 domain containing 1 |
| rs333960 | 1 | 110241003 | flanking_5UTR |  |  | [T/C] | QUICKl | 2.21E-07 | 0.036 | G | 0.144 | *CSF1* | colony stimulating factor 1 (macrophage) |
| rs333949 | 1 | 110252566 | flanking_5UTR |  |  | [T/C] | QUICKl | 7.63E-06 | 0.028 | G | 0.149 | *CSF1* | colony stimulating factor 1 (macrophage) |
| rs12567355 | 1 | 111265147 | flanking_3UTR |  |  | [T/C] | Bone mineral density (g/cm^2^) | 8.65E-06 | 0.042 | A | 0.026 | *CD53* | CD53 molecule |
| rs10776733 | 1 | 111909192 | flanking_3UTR |  |  | [T/C] | Energy storage (kcal/d) | 6.90E-06 | 0.034 | A | 0.360 | *ADORA3* | adenosine A3 receptor |
| rs10776733 | 1 | 111909192 | flanking_3UTR |  |  | [T/C] | Fat mass change (kg/y) | 7.01E-06 | 0.033 | A | 0.360 | *ADORA3* | adenosine A3 receptor |
| rs10776733 | 1 | 111909192 | flanking_3UTR |  |  | [T/C] | Fat mass deposition (kcal/d) | 6.93E-06 | 0.033 | A | 0.360 | *ADORA3* | adenosine A3 receptor |
| rs79925084 | 1 | 113949534 | intron |  |  | [T/C] | Total cysteine (µmol/L) | 3.04E-06 | 0.026 | G | 0.006 | *MAGI3* | membrane associated guanylate kinase, WW and PDZ domain containing 3 and VANGL2 |
| rs78563144 | 1 | 113957740 | intron |  |  | [A/G] | Total cysteine (µmol/L) | 3.04E-06 | 0.026 | A | 0.006 | *MAGI3* | membrane associated guanylate kinase, WW and PDZ domain containing 3 and VANGL2 |
| rs77911012 | 1 | 113963330 | intron |  |  | [T/C] | Total cysteine (µmol/L) | 3.04E-06 | 0.026 | G | 0.006 | *MAGI3* | membrane associated guanylate kinase, WW and PDZ domain containing 3 and VANGL2 |
| rs79247801 | 1 | 114005494 | intron |  |  | [T/C] | Total cysteine (µmol/L) | 3.04E-06 | 0.026 | G | 0.006 | *MAGI3* | membrane associated guanylate kinase, WW and PDZ domain containing 3 and VANGL2 |
| rs76231474 | 1 | 114007559 | intron |  |  | [T/C] | Total cysteine (µmol/L) | 3.04E-06 | 0.026 | A | 0.006 | *MAGI3* | membrane associated guanylate kinase, WW and PDZ domain containing 3 and VANGL2 |
| rs77600616 | 1 | 114015458 | intron |  |  | [C/G] | Total cysteine (µmol/L) | 3.04E-06 | 0.026 | C | 0.006 | *MAGI3* | membrane associated guanylate kinase, WW and PDZ domain containing 3 and VANGL2 |
| rs74901666 | 1 | 114049787 | intron |  |  | [T/C] | Total cysteine (µmol/L) | 3.04E-06 | 0.026 | A | 0.006 | *PHTF1* | putative homeodomain transcription factor 1 |
| rs78089789 | 1 | 114083893 | intron |  |  | [C/G] | Total cysteine (µmol/L) | 3.04E-06 | 0.026 | G | 0.006 | *PHTF1* | putative homeodomain transcription factor 1 |
| rs78399433 | 1 | 114147660 | intron |  |  | [A/G] | Total cysteine (µmol/L) | 3.04E-06 | 0.026 | G | 0.006 | *RSBN1* | round spermatid basic protein 1 |
| rs75453168 | 1 | 114149727 | intron |  |  | [T/C] | Total cysteine (µmol/L) | 3.04E-06 | 0.026 | G | 0.006 | *RSBN1* | round spermatid basic protein 1 |
| rs79241487 | 1 | 114203305 | intron |  |  | [A/G] | Total cysteine (µmol/L) | 3.04E-06 | 0.026 | A | 0.006 | *PTPN22* | protein tyrosine phosphatase, non-receptor type 22 (lymphoid) |
| rs12079716 | 1 | 114246117 | intron |  |  | [A/C] | Total cysteine (µmol/L) | 1.50E-06 | 0.027 | C | 0.003 | *AP4B1* | adaptor-related protein complex 4, beta 1 subunit |
| rs77730918 | 1 | 114349614 | flanking_3UTR |  |  | [T/C] | Total cysteine (µmol/L) | 2.47E-06 | 0.024 | A | 0.004 | *OLFML3* | olfactomedin-like 3 |
| rs12092195 | 1 | 114384995 | flanking_3UTR |  |  | [A/G] | Total cysteine (µmol/L) | 1.74E-06 | 0.027 | G | 0.010 | *SYT6* | synaptotagmin VI |
| rs78972990 | 1 | 114388719 | flanking_3UTR |  |  | [A/G] | Total cysteine (µmol/L) | 6.96E-06 | 0.022 | G | 0.003 | *SYT6* | synaptotagmin VI |
| rs79079833 | 1 | 114408887 | flanking_3UTR |  |  | [A/G] | Total cysteine (µmol/L) | 3.60E-06 | 0.026 | G | 0.011 | *SYT6* | synaptotagmin VI |
| rs79079833 | 1 | 114408887 | flanking_3UTR |  |  | [A/G] | Total homocysteine (µmol/L) | 1.64E-06 | 0.032 | G | 0.011 | *SYT6* | synaptotagmin VI |
| rs58491015 | 1 | 114423981 | flanking_3UTR |  |  | [A/G] | Total homocysteine (µmol/L) | 4.56E-06 | 0.030 | G | 0.014 | *SYT6* | synaptotagmin VI |
| rs7527203 | 1 | 118618252 | flanking_5UTR |  |  | [T/C] | Total T3 (ng/dL) | 2.14E-06 | 0.030 | A | 0.013 | *SPAG17* | sperm associated antigen 17 |
| rs1061955 | 1 | 148180981 | flanking_3UTR |  |  | [A/G] | ALT/AST | 3.60E-06 | 0.029 | A | 0.014 | *OTUD7B* | OTU domain containing 7B |
| rs73004856 | 1 | 150754541 | coding |  |  | [A/G] | NEFA (mmol/L) | 2.79E-06 | 0.022 | A | 0.024 | *CRCT1* | cysteine-rich C-terminal 1 |
| rs6691316 | 1 | 153020818 | intron |  |  | [T/C] | ALT/AST | 8.70E-06 | 0.025 | G | 0.318 | *KCNN3* | potassium intermediate/small conductance calcium-activated channel |
| rs6691316 | 1 | 153020818 | intron |  |  | [T/C] | AST/ALT | 6.90E-06 | 0.026 | G | 0.318 | *KCNN3* | potassium intermediate/small conductance calcium-activated channel |
| rs11264330 | 1 | 153365529 | flanking_5UTR |  |  | [A/G] | MCP-1 (pg/mL) | 4.91E-06 | 0.040 | A | 0.060 | *EFNA1* | ephrin-A1 |
| rs6427419 | 1 | 156324733 | intron |  |  | [A/C] | Diet fat (%energy) | 8.25E-06 | 0.026 | C | 0.353 | *KIRREL* | kin of IRRE like (Drosophila) |
| rs13376489 | 1 | 157052271 | flanking_5UTR |  |  | [A/C] | Diet carbohydrate (g/d) | 1.34E-06 | 0.037 | A | 0.376 | *MNDA* | myeloid cell nuclear differentiation antigen |
| rs2106091 | 1 | 157087824 | flanking_3UTR |  |  | [A/G] | Diet carbohydrate (g/d) | 5.24E-06 | 0.036 | G | 0.327 | *MNDA* | myeloid cell nuclear differentiation antigen |
| rs1101993 | 1 | 157263519 | intron |  |  | [T/C] | Diet carbohydrate (g/d) | 2.53E-06 | 0.035 | G | 0.137 | *IFI16* | interferon, gamma-inducible protein 16 |
| rs12094741 | 1 | 157265289 | intron |  |  | [A/G] | Diet carbohydrate (g/d) | 2.80E-06 | 0.035 | A | 0.117 | *IFI16* | interferon, gamma-inducible protein 16 |
| rs861318 | 1 | 157268846 | intron |  |  | [A/G] | Diet carbohydrate (g/d) | 1.05E-06 | 0.042 | A | 0.120 | *IFI16* | interferon, gamma-inducible protein 16 |
| rs1057027 | 1 | 157269001 | coding | NONSYN | R409S(NP_005522.2) | [A/C] | Diet carbohydrate (g/d) | 1.05E-06 | 0.042 | C | 0.120 | *IFI16* | interferon, gamma-inducible protein 16 |
| rs2793845 | 1 | 157298879 | flanking_3UTR |  |  | [A/C] | Diet carbohydrate (g/d) | 6.82E-06 | 0.039 | C | 0.069 | *AIM2* | absent in melanoma 2 |
| rs3027012 | 1 | 157440747 | flanking_5UTR |  |  | [T/C] | MCP-1 (pg/mL) | 1.74E-07 | 0.037 | A | 0.172 | *DARC* | Duffy blood group, chemokine receptor |
| rs863002 | 1 | 157441544 | intron |  |  | [A/G] | MCP-1 (pg/mL) | 3.59E-13 | 0.062 | A | 0.244 | *DARC* | Duffy blood group, chemokine receptor |
| rs3027016 | 1 | 157441817 | intron |  |  | [T/C] | MCP-1 (pg/mL) | 3.06E-06 | 0.028 | G | 0.110 | *DARC* | Duffy blood group, chemokine receptor |
| rs12075 | 1 | 157441978 | coding | NONSYN | G42D(NP_002027.2) | [A/G] | MCP-1 (pg/mL) | 1.31E-21 | 0.103 | A | 0.436 | *DARC* | Duffy blood group, chemokine receptor |
| rs863006 | 1 | 157444372 | flanking_3UTR |  |  | [A/G] | MCP-1 (pg/mL) | 1.03E-16 | 0.084 | A | 0.423 | *DARC* | Duffy blood group, chemokine receptor |
| rs2427837 | 1 | 157525169 | flanking_5UTR |  |  | [T/C] | MCP-1 (pg/mL) | 1.28E-07 | 0.032 | A | 0.121 | *FCER1A* | Fc fragment of IgE, high affinity I, receptor for; alpha polypeptide |
| rs4656236 | 1 | 157677599 | flanking_3UTR |  |  | [A/G] | MCP-1 (pg/mL) | 8.74E-06 | 0.026 | A | 0.121 | *OR10J1* | olfactory receptor, family 10, subfamily J, member 1 |
| rs4325129 | 1 | 157729005 | flanking_3UTR |  |  | [A/G] | MCP-1 (pg/mL) | 5.48E-06 | 0.025 | G | 0.148 | *OR10J5* | olfactory receptor, family 10, subfamily J, member 5 |
| rs76662883 | 1 | 160355942 | flanking_5UTR |  |  | [T/C] | Hip circumference (cm) | 8.62E-06 | 0.028 | A | 0.006 | *NOS1AP* | nitric oxide synthase 1 (neuronal) adaptor protein |
| rs1891931 | 1 | 163427202 | flanking_3UTR |  |  | [A/C] | MCP-1 (pg/mL) | 9.70E-06 | 0.018 | C | 0.183 | *LMX1A* | LIM homeobox transcription factor 1, alpha |
| rs12741275 | 1 | 164860378 | 5UTR |  |  | [T/C] | Calorimeter activity (counts/d) | 9.85E-06 | 0.035 | G | 0.064 | *FMO9P* | flavin containing monooxygenase 9 pseudogene |
| rs12760731 | 1 | 176737845 | flanking_5UTR |  |  | [T/C] | Weight change (kg/y) | 4.64E-06 | 0.031 | A | 0.062 | *RASAL2* | RAS protein activator like 2 |
| rs12023718 | 1 | 176758502 | flanking_3UTR |  |  | [A/G] | Hip circumference change (cm/y) | 6.38E-06 | 0.034 | G | 0.072 | *C1orf49* | chromosome 1 open reading frame 49 |
| rs12033565 | 1 | 176758589 | flanking_3UTR |  |  | [T/C] | Hip circumference change (cm/y) | 7.64E-06 | 0.033 | A | 0.068 | *C1orf49* | chromosome 1 open reading frame 49 |
| rs4652795 | 1 | 181532805 | intron |  |  | [A/G] | IL-6 (pg/mL) | 5.35E-06 | 0.030 | G | 0.471 | *NMNAT2* | nicotinamide nucleotide adenylyltransferase 2 |
| rs2078087 | 1 | 181625028 | intron |  |  | [A/G] | AST/ALT | 1.02E-06 | 0.032 | A | 0.139 | *NMNAT2* | nicotinamide nucleotide adenylyltransferase 2 |
| rs10494563 | 1 | 181643603 | intron |  |  | [A/C] | AST/ALT | 4.99E-06 | 0.027 | A | 0.123 | *NMNAT2* | nicotinamide nucleotide adenylyltransferase 2 |
| rs6663178 | 1 | 182928265 | 3UTR |  |  | [T/C] | Fat mass (%) | 6.88E-06 | 0.030 | A | 0.470 | *EDEM3* | ER degradation enhancer, mannosidase alpha-like 3 |
| rs3736757 | 1 | 182944087 | coding | SYNON | I577I(NP_079467.2) | [A/G] | Fat mass (%) | 5.45E-06 | 0.031 | A | 0.473 | *EDEM3* | ER degradation enhancer, mannosidase alpha-like 3 |
| rs1040666 | 1 | 182961206 | intron |  |  | [A/C] | Fat mass (%) | 9.05E-06 | 0.029 | A | 0.472 | *EDEM3* | ER degradation enhancer, mannosidase alpha-like 3" |
| rs861601 | 1 | 183011887 | flanking_3UTR |  |  | [T/C] | Head circumference (cm) | 4.44E-06 | 0.025 | A | 0.431 | *FAM129A* | family with sequence similarity 129, member A |
| rs570441 | 1 | 183026624 | flanking_3UTR |  |  | [A/G] | Head circumference (cm) | 7.23E-06 | 0.023 | G | 0.437 | *FAM129A* | family with sequence similarity 129, member A |
| rs7360 | 1 | 183027056 | 3UTR |  |  | [T/C] | Head circumference (cm) | 4.27E-06 | 0.024 | A | 0.434 | *FAM129A* | family with sequence similarity 129, member A |
| rs682331 | 1 | 183027988 | 3UTR |  |  | [T/C] | Head circumference (cm) | 4.07E-06 | 0.024 | G | 0.437 | *FAM129A* | family with sequence similarity 129, member A |
| rs10797988 | 1 | 183050322 | intron |  |  | [T/C] | Head circumference (cm) | 8.43E-06 | 0.026 | G | 0.366 | *FAM129A* | family with sequence similarity 129, member A |
| rs3753573 | 1 | 183280625 | flanking_5UTR |  |  | [T/C] | Total antioxidants (mM) | 2.22E-06 | 0.028 | A | 0.002 | *RNF2* | ring finger protein 2 |
| rs12142669 | 1 | 186649939 | flanking_3UTR |  |  | [A/G] | Diastolic blood pressure (mmHg) | 2.65E-06 | 0.033 | A | 0.104 |  |  |
| rs10798210 | 1 | 186651087 | flanking_3UTR |  |  | [T/C] | Diastolic blood pressure (mmHg) | 2.65E-06 | 0.033 | A | 0.104 |  |  |
| rs3001167 | 1 | 192797537 | flanking_3UTR |  |  | [T/C] | Adiponectin (ng/mL) | 2.79E-06 | 0.027 | G | 0.316 | *EEF1A1P14* | eukaryotic translation elongation factor 1 alpha 1 pseudogene 14 |
| rs586688 | 1 | 199897578 | intron |  |  | [A/G] | Estradiol (pg/mL) | 7.69E-06 | 0.026 | A | 0.092 | *NAV1* | neuron navigator 1 |
| rs12739262 | 1 | 200658366 | intron |  |  | [A/G] | Estradiol (pg/mL) | 1.23E-06 | 0.030 | A | 0.262 | *PPP1R12B* | protein phosphatase 1, regulatory (inhibitor) subunit 12B |
| rs1539143 | 1 | 201639108 | flanking_5UTR |  |  | [A/G] | Trunk fat mass (kg) | 1.25E-06 | 0.036 | G | 0.173 | *FMOD* | fibromodulin |
| rs76872152 | 1 | 203447033 | coding |  |  | [T/C] | MCP-1 (pg/mL) | 4.67E-06 | 0.041 | A | 0.062 | *DSTYK* | dual serine/threonine and tyrosine protein kinase |
| rs7545126 | 1 | 206004859 | intron |  |  | [A/G] | Triglycerides (mg/dL) | 8.83E-06 | 0.026 | A | 0.065 | *CD46* | CD46 molecule, complement regulatory protein |
| rs7542375 | 1 | 219178403 | flanking_3UTR |  |  | [A/C] | Total cysteine (µmol/L) | 2.10E-06 | 0.021 | C | 0.431 | *HLX* | H2.0-like homeobox |
| rs10915864 | 1 | 223967629 | flanking_5UTR |  |  | [T/C] | IL-6 (pg/mL) | 1.76E-06 | 0.028 | G | 0.264 | *ENAH* | enabled homolog (Drosophila) |
| rs12023396 | 1 | 229337259 | flanking_5UTR |  |  | [T/C] | IL-6 (pg/mL) | 4.35E-07 | 0.034 | G | 0.181 | *TRIM67* | tripartite motif containing 67 |
| rs11577354 | 1 | 233333750 | flanking_3UTR |  |  | [A/G] | RQmax | 7.32E-06 | 0.041 | G | 0.445 | *TOMM20* | translocase of outer mitochondrial membrane 20 homolog (yeast) |
| rs1106159 | 1 | 235415050 | intron |  |  | [T/C] | Cystathionine (µmol/L) | 5.21E-06 | 0.030 | G | 0.358 | *RYR2* | ryanodine receptor 2 (cardiac) |
| rs6668069 | 1 | 235416280 | intron |  |  | [T/C] | Cystathionine (µmol/L) | 4.76E-06 | 0.030 | A | 0.341 | *RYR2* | ryanodine receptor 2 (cardiac) |
| rs6683225 | 1 | 235416361 | intron |  |  | [A/G] | Cystathionine (µmol/L) | 2.11E-06 | 0.032 | G | 0.433 | *RYR2* | ryanodine receptor 2 (cardiac) |
| rs268786 | 1 | 235422608 | intron |  |  | [T/G] | Free T3 (pg/mL) | 8.24E-06 | 0.023 | C | 0.014 | *RYR2* | ryanodine receptor 2 (cardiac) |
| rs187910 | 1 | 235426611 | intron |  |  | [A/G] | Cystathionine (µmol/L) | 3.10E-06 | 0.030 | A | 0.384 | *RYR2* | ryanodine receptor 2 (cardiac) |
| rs13373941 | 1 | 237710794 | flanking_3UTR |  |  | [A/C] | TNF-α (pg/mL) | 7.99E-06 | 0.025 | C | 0.006 | *LOC100505872* | uncharacterized LOC100505872 |
| rs7544372 | 1 | 239054377 | intron |  |  | [T/C] | Vitamin B12 (pmol/L) | 9.42E-06 | 0.030 | G | 0.211 | *RGS7* | regulator of G-protein signaling 7 |
| rs1053221 | 1 | 239822190 | 3UTR |  |  | [T/C] | Urinary creatinine (mmol/d) | 1.13E-06 | 0.041 | G | 0.111 | *KMO* | kynurenine 3-monooxygenase |
| rs3193688 | 1 | 239825189 | 3UTR |  |  | [G/C] | Urinary creatinine (mmol/d) | 1.13E-06 | 0.041 | G | 0.111 | *KMO* | kynurenine 3-monooxygenase |
| rs12752277 | 1 | 241022060 | flanking_3UTR |  |  | [T/C] | Estradiol (pg/mL) | 1.23E-06 | 0.030 | A | 0.262 | *RSL24D1P4* | ribosomal L24 domain containing 1 pseudogene 4 |
| rs12751297 | 1 | 241022186 | flanking_5UTR |  |  | [T/C] | Estradiol (pg/mL) | 1.11E-06 | 0.030 | A | 0.262 | *PLD5* | phospholipase D family, member 5 |
| rs2809867 | 1 | 241022853 | flanking_3UTR |  |  | [A/G] | Estradiol (pg/mL) | 1.34E-06 | 0.030 | A | 0.262 | *RSL24D1P4* | ribosomal L24 domain containing 1 pseudogene 4 |
| rs2306084 | 1 | 242639653 | intron |  |  | [T/G] | IGFBP-1 (ng/mL) | 8.51E-06 | 0.023 | A | 0.247 | *ADSS* | adenylosuccinate synthase |
| rs10927232 | 1 | 242643130 | intron |  |  | [A/G] | IGFBP-1 (ng/mL) | 6.50E-06 | 0.025 | G | 0.265 | *ADSS* | adenylosuccinate synthase |
| rs3102460 | 1 | 242643340 | intron |  |  | [T/C] | IGFBP-1 (ng/mL) | 3.66E-06 | 0.025 | G | 0.358 | *ADSS* | adenylosuccinate synthase |
| rs12087973 | 1 | 242645085 | intron |  |  | [A/G] | IGFBP-1 (ng/mL) | 5.92E-06 | 0.025 | A | 0.262 | *ADSS* | adenylosuccinate synthase |
| rs4132572 | 1 | 242659058 | intron |  |  | [A/G] | IGFBP-1 (ng/mL) | 6.13E-06 | 0.025 | G | 0.265 | *ADSS* | adenylosuccinate synthase |
| rs11800820 | 1 | 244727859 | flanking_3UTR |  |  | [A/G] | Fat oxidation (%NPEE) | 4.08E-06 | 0.032 | A | 0.164 | *SMYD3* | SET and MYND domain containing 3 |
| rs11800820 | 1 | 244727859 | flanking_3UTR |  |  | [A/G] | Total energy expenditure RQ | 4.24E-06 | 0.032 | A | 0.164 | *SMYD3* | SET and MYND domain containing 3 |
| rs1320333 | 2 | 669179 | flanking_5UTR |  |  | [T/C] | Urinary creatinine (mmol/d) | 8.08E-06 | 0.032 | A | 0.057 | *TMEM18* | transmembrane protein 18 |
| rs10865541 | 2 | 3371082 | coding | SYNON | F227F(NP_057114.5) | [T/C] | Testosterone (ng/mL) | 5.58E-06 | 0.027 | A | 0.488 | *TTC15/TRAPPC12* | tetratricopeptide repeat domain 15/ trafficking protein particle complex 12 |
| rs6730148 | 2 | 3726846 | intron |  |  | [A/G] | Cortisol (ng/mL) | 1.54E-06 | 0.022 | G | 0.074 | *ALLC, DCDC2C* | allantoicase; doublecortin domain containing 2C |
| rs13004938 | 2 | 5354994 | flanking_5UTR |  |  | [T/C] | Bike energy expenditure (kcal/min) | 8.93E-06 | 0.035 | G | 0.094 | *SOX11* | SRY (sex determining region Y)-box 11 |
| rs2193071 | 2 | 7953711 | flanking_3UTR |  |  | [T/C] | Vigorous activity (%awake time) | 3.66E-06 | 0.027 | A | 0.007 | *LINC00299* | long intergenic non-protein coding RNA 299 |
| rs16857178 | 2 | 11174956 | intron |  |  | [A/G] | Urinary creatinine (mmol/d) | 9.46E-06 | 0.036 | A | 0.043 | *FLJ33534* | uncharacterized LOC285150 (FLJ33534), non-coding RNA |
| rs73175262 | 2 | 11675882 | coding |  |  | [A/G] | IGFBP-3 (ng/mL) | 9.38E-06 | 0.026 | A | 0.053 | *GREB1* | growth regulation by estrogen in breast cancer 1 |
| rs73175262 | 2 | 11675882 | coding |  |  | [A/G] | MCP-1 (pg/mL) | 6.46E-08 | 0.049 | A | 0.053 | *GREB1* | growth regulation by estrogen in breast cancer 1 |
| rs16858228 | 2 | 12129896 | flanking_3UTR |  |  | [T/C] | Urinary creatinine (mmol/d) | 1.35E-06 | 0.040 | G | 0.146 | *ST13P1* | suppression of tumorigenicity 13 (colon carcinoma) (Hsp70 interacting protein) pseudogene 1 |
| rs10189050 | 2 | 22978063 | flanking_5UTR |  |  | [A/C] | Vigorous activity (min/d) | 4.42E-06 | 0.033 | C | 0.053 | *KLHL29* | kelch-like 29 (Drosophila) |
| rs3795942 | 2 | 23772880 | coding | SYNON | H856H(XP_001134449.1) | [A/G] | Total T4 (µg/dL) | 4.79E-06 | 0.026 | G | 0.268 | *KLHL29* | kelch-like 29 (Drosophila) |
| rs3795948 | 2 | 23780163 | coding | NONSYN | L947P(XP_001134449.1) | [A/G] | Total T4 (µg/dL) | 9.68E-06 | 0.027 | A | 0.303 | *KLHL29* | kelch-like 29 (Drosophila) |
| rs12478744 | 2 | 23801093 | flanking_3UTR |  |  | [T/C] | Total T4 (µg/dL) | 1.10E-06 | 0.031 | A | 0.229 | *KLHL29* | kelch-like 29 (Drosophila) |
| rs7608623 | 2 | 23802457 | flanking_3UTR |  |  | [T/G] | Total T4 (µg/dL) | 6.08E-07 | 0.032 | C | 0.229 | *KLHL29* | kelch-like 29 (Drosophila) |
| rs11674248 | 2 | 24007696 | flanking_5UTR |  |  | [A/C] | Total T4 (µg/dL) | 3.33E-06 | 0.027 | A | 0.254 | *ATAD2B* | ATPase family, AAA domain containing 2B |
| rs7572949 | 2 | 24015522 | flanking_5UTR |  |  | [T/C] | Total T4 (µg/dL) | 4.92E-06 | 0.026 | A | 0.253 | *UBXN2A* | UBX domain protein 2A |
| rs11887277 | 2 | 26936773 | intron |  |  | [T/C] | Urinary creatinine (mmol/d) | 4.85E-06 | 0.040 | A | 0.458 | *DPYSL5* | dihydropyrimidinase-like 5 |
| rs6756245 | 2 | 26936993 | intron |  |  | [A/G] | Urinary creatinine (mmol/d) | 5.76E-06 | 0.039 | A | 0.466 | *DPYSL5* | dihydropyrimidinase-like 5 |
| rs2295475 | 2 | 31443351 | coding | SYNON | I737I(NP_000370.2) | [T/C] | Urinary free dopamine (nmol/d) | 5.22E-06 | 0.036 | A | 0.363 | *XDH* | xanthine dehydrogenase |
| rs761926 | 2 | 31444289 | intron |  |  | [C/G] | Urinary free dopamine (nmol/d) | 4.29E-06 | 0.037 | G | 0.370 | *XDH* | xanthine dehydrogenase |
| rs12479213 | 2 | 38954713 | intron |  |  | [A/C] | Estradiol (pg/mL) | 9.10E-06 | 0.026 | A | 0.281 | *DHX57* | DEAH (Asp-Glu-Ala-Asp/His) box polypeptide 57 |
| rs2716697 | 2 | 39695006 | intron |  |  | [A/G] | Total T3 (ng/dL) | 7.06E-06 | 0.025 | G | 0.011 | *LOC728730* | uncharacterized LOC728730 |
| rs17024218 | 2 | 39793040 | intron |  |  | [A/G] | Total T3 (ng/dL) | 1.98E-06 | 0.026 | G | 0.014 | *TMEM178* | transmembrane protein 178 |
| rs10490332 | 2 | 39805413 | flanking_3UTR |  |  | [T/C] | Total T3 (ng/dL) | 3.15E-06 | 0.026 | A | 0.010 | *TMEM178* | transmembrane protein 178 |
| rs7579930 | 2 | 40503420 | intron |  |  | [T/G] | Free T3 (pg/mL) | 6.93E-06 | 0.026 | C | 0.350 | *SLC8A1* | solute carrier family 8 (sodium/calcium exchanger), member 1 |
| rs4629203 | 2 | 40516050 | flanking_5UTR |  |  | [A/G] | Free T3 (pg/mL) | 2.92E-06 | 0.030 | G | 0.429 | *SLC8A1* | solute carrier family 8 (sodium/calcium exchanger), member 1 |
| rs996712 | 2 | 53356279 | flanking_3UTR |  |  | [T/C] | Free T3 (pg/mL) | 7.47E-06 | 0.023 | G | 0.340 | *ASB3* | ankyrin repeat and SOCS box containing 3 |
| rs7589998 | 2 | 60777524 | flanking_5UTR |  |  | [T/C] | Diet fat (g/d) | 5.19E-06 | 0.026 | G | 0.006 | *LOC442017* | interferon induced transmembrane protein 3 pseudogene |
| rs2138798 | 2 | 63364939 | intron |  |  | [T/G] | Urinary free norepinephrine: creatinine | 8.35E-07 | 0.034 | A | 0.217 | *WDPCP* | WD repeat-containing& planar cell polarity effector |
| rs6759808 | 2 | 68504501 | flanking_3UTR |  |  | [A/G] | ALT/AST | 5.25E-06 | 0.027 | G | 0.194 | *LOC391383* | WD repeat domain 4 pseudogene |
| rs6759808 | 2 | 68504501 | flanking_3UTR |  |  | [A/G] | AST/ALT | 3.49E-06 | 0.028 | G | 0.194 | *LOC391383* | WD repeat domain 4 pseudogene |
| rs11126185 | 2 | 68506084 | flanking_3UTR |  |  | [A/G] | ALT/AST | 4.84E-06 | 0.027 | G | 0.188 | *LOC391383* | WD repeat domain 4 pseudogene |
| rs11126185 | 2 | 68506084 | flanking_3UTR |  |  | [A/G] | AST/ALT | 3.58E-06 | 0.028 | G | 0.188 | *LOC391383* | WD repeat domain 4 pseudogene |
| rs7583236 | 2 | 70036309 | flanking_3UTR |  |  | [A/G] | Sedentary&light activity (min/d) | 3.98E-06 | 0.038 | G | 0.247 | *SASP* | aspartic peptidase, retroviral-like 1 (ASPRV1) |
| rs6708331 | 2 | 70222427 | flanking_3UTR |  |  | [A/G] | Waist circumference change (cm/y) | 5.85E-06 | 0.035 | A | 0.094 | *C2orf42* | chromosome 2 open reading frame 42 |
| rs3771514 | 2 | 70551175 | intron |  |  | [T/C] | Height z-score change (SD/y) | 7.87E-06 | 0.031 | A | 0.386 | *TGFA* | transforming growth factor, alpha |
| rs17011478 | 2 | 75645651 | intron |  |  | [A/G] | Head circumference (cm) | 6.23E-06 | 0.036 | A | 0.176 | *FAM176A* | family with sequence similarity 176, member A |
| rs11890236 | 2 | 104175144 | flanking_3UTR |  |  | [A/G] | Diet fat (%energy) | 9.24E-06 | 0.015 | G | 0.035 |  |  |
| rs1030877 | 2 | 105276945 | intron |  |  | [T/C] | IGFBP-1 (ng/mL) | 6.00E-06 | 0.027 | G | 0.350 | *TGFBRAP1* | transforming growth factor, beta receptor associated protein 1 |
| rs1448190 | 2 | 112092434 | flanking_3UTR |  |  | [T/G] | IL-6 (pg/mL) | 7.68E-06 | 0.026 | A | 0.381 | *ANAPC1* | anaphase promoting complex subunit 1 |
| rs4252023 | 2 | 113606844 | coding | SYNON | D135D(NP_000568.1) | [A/G] | IGFBP-3 (ng/mL) | 2.00E-06 | 0.030 | A | 0.058 | *IL1RN* | interleukin 1 receptor antagonist |
| rs6732028 | 2 | 115303409 | flanking_3UTR |  |  | [A/G] | Birth weight (kg) | 3.26E-06 | 0.059 | G | 0.247 | *DPP10* | dipeptidyl-peptidase 10 (non-functional) |
| rs1374313 | 2 | 119862125 | flanking_3UTR |  |  | [A/G] | Diet fat (g/d) | 9.20E-07 | 0.030 | G | 0.343 | *DBI* | diazepam binding inhibitor (GABA receptor modulator, acyl-CoA binding protein) |
| rs10198552 | 2 | 135316346 | intron |  |  | [T/C] | AST (U/L) | 6.97E-07 | 0.037 | G | 0.025 | *ACMSD* | aminocarboxymuconate semialdehyde decarboxylase |
| rs1011397 | 2 | 144406587 | flanking_3UTR |  |  | [A/G] | Energy balance (kcal/d) | 7.69E-06 | 0.038 | G | 0.197 | *GTDC1* | glycosyltransferase-like domain containing 1 |
| rs7355746 | 2 | 145068291 | flanking_5UTR |  |  | [A/G] | Urinary nitrogen (g/d) | 4.57E-07 | 0.037 | A | 0.014 | *ZEB2* | zinc finger E-box binding homeobox 2 |
| rs10445672 | 2 | 145821724 | flanking_3UTR |  |  | [T/C] | Vigorous activity (%awake time) | 7.55E-06 | 0.030 | G | 0.093 |  |  |
| rs16836124 | 2 | 154711093 | intron |  |  | [A/G] | ALT/AST | 5.90E-06 | 0.028 | G | 0.054 | *GALNT13* | UDP-N-acetyl-alpha-D-galactosamine:polypeptide N-acetylgalactosaminyltransferase 13 |
| rs13007462 | 2 | 159299308 | flanking_3UTR |  |  | [T/C] | TBF-ß1 (pg/mL) | 6.77E-06 | 0.028 | G | 0.417 | *PKP4* | plakophilin 4 |
| rs4664262 | 2 | 159301640 | flanking_3UTR |  |  | [A/G] | TBF-ß1 (pg/mL) | 2.68E-06 | 0.030 | A | 0.418 | *PKP4* | plakophilin 4 |
| rs6705792 | 2 | 159310432 | flanking_5UTR |  |  | [T/C] | TBF-ß1 (pg/mL) | 9.23E-06 | 0.026 | G | 0.386 | *DAPL1* | death associated protein-like 1 |
| rs6437198 | 2 | 159321442 | flanking_5UTR |  |  | [T/C] | TBF-ß1 (pg/mL) | 4.04E-06 | 0.030 | A | 0.396 | *DAPL1* | death associated protein-like 1 |
| rs1515926 | 2 | 159330454 | flanking_5UTR |  |  | [A/G] | TBF-ß1 (pg/mL) | 2.39E-06 | 0.031 | A | 0.414 | *DAPL1* | death associated protein-like 1 |
| rs2356507 | 2 | 159347591 | flanking_5UTR |  |  | [A/G] | TBF-ß1 (pg/mL) | 5.81E-06 | 0.029 | G | 0.417 | *DAPL1* | death associated protein-like 1 |
| rs6716178 | 2 | 159375574 | intron |  |  | [A/G] | TBF-ß1 (pg/mL) | 7.81E-06 | 0.028 | G | 0.411 | *DAPL1* | death associated protein-like 1 |
| rs4665011 | 2 | 159377004 | intron |  |  | [T/G] | TBF-ß1 (pg/mL) | 4.19E-06 | 0.031 | A | 0.249 | *DAPL1* | death associated protein-like 1 |
| rs16843372 | 2 | 159378695 | intron |  |  | [A/G] | TBF-ß1 (pg/mL) | 1.19E-06 | 0.036 | G | 0.359 | *DAPL1* | death associated protein-like 1 |
| rs10208407 | 2 | 161465375 | flanking_5UTR |  |  | [T/C] | Vigorous activity (%awake time) | 2.01E-06 | 0.029 | A | 0.008 | *TANK* | TRAF family member-associated NFKB activator |
| rs13408808 | 2 | 168273939 | flanking_3UTR |  |  | [A/G] | Diet fat (%energy) | 7.97E-06 | 0.039 | G | 0.083 | *CTAGE14P* | CTAGE family, member 14, pseudogene |
| rs11691258 | 2 | 168348084 | flanking_5UTR |  |  | [T/C] | Amylin (pM) | 8.78E-06 | 0.028 | G | 0.068 | *B3GALT1* | UDP-Gal:betaGlcNAc beta 1,3-galactosyltransferase, polypeptide 1 |
| rs1004368 | 2 | 168350216 | flanking_5UTR |  |  | [A/G] | Amylin (pM) | 1.94E-06 | 0.029 | G | 0.103 | *B3GALT1* | UDP-Gal:betaGlcNAc beta 1,3-galactosyltransferase, polypeptide 1 |
| rs2114646 | 2 | 170332467 | flanking_3UTR |  |  | [A/G] | Amylin (pM) | 5.64E-06 | 0.034 | G | 0.494 | *KLHL23* | kelch-like 23 (Drosophila) |
| rs7561268 | 2 | 170964843 | intron |  |  | [A/C] | Amylin (pM) | 2.38E-06 | 0.031 | C | 0.297 | *MYO3B* | myosin IIIB |
| rs13425835 | 2 | 171158407 | intron |  |  | [T/G] | Total antioxidants (mM) | 6.45E-06 | 0.026 | C | 0.249 | *MYO3B* | myosin IIIB |
| rs6749331 | 2 | 171169378 | intron |  |  | [T/C] | Total antioxidants (mM) | 3.37E-06 | 0.027 | G | 0.121 | *MYO3B* | myosin IIIB |
| rs4972489 | 2 | 176442844 | flanking_3UTR |  |  | [A/C] | Diet protein (g/d) | 8.06E-06 | 0.028 | C | 0.366 | *KIAA1715* | KIAA1715 |
| rs4972489 | 2 | 176442844 | flanking_3UTR |  |  | [A/C] | Energy intake (kcal/d) | 7.88E-06 | 0.025 | C | 0.366 | *KIAA1715* | KIAA1715 |
| rs1020410 | 2 | 176492384 | flanking_3UTR |  |  | [A/G] | Calorimeter activity (counts/d) | 6.05E-07 | 0.040 | G | 0.268 | *KIAA1715* | KIAA1715 |
| rs4369827 | 2 | 176517851 | intron |  |  | [A/G] | Calorimeter activity (counts/d) | 8.48E-07 | 0.036 | G | 0.260 | *KIAA1715* | KIAA1715 |
| rs17430279 | 2 | 185144507 | flanking_5UTR |  |  | [T/C] | Height z-score change (SD/y) | 3.83E-06 | 0.031 | G | 0.152 | *ZNF804A* | zinc finger protein 804A |
| rs2675399 | 2 | 189427207 | flanking_5UTR |  |  | [T/C] | Adiponectin (ng/mL) | 6.38E-06 | 0.026 | G | 0.196 | *DIRC1* | disrupted in renal carcinoma 1 |
| rs12053254 | 2 | 190315398 | intron |  |  | [A/G] | Diet carbohydrate (g/d) | 5.96E-06 | 0.028 | G | 0.125 | *ANKAR* | ankyrin and armadillo repeat containing |
| rs5742967 | 2 | 190361995 | intron |  |  | [A/G] | Diet carbohydrate (g/d) | 5.96E-06 | 0.028 | A | 0.125 | *PMS1* | PMS1 postmeiotic segregation increased 1 (S. cerevisiae) |
| rs1823913 | 2 | 191826292 | intron |  |  | [T/C] | Diet carbohydrate (g/d) | 9.20E-06 | 0.025 | A | 0.380 | *MYO1B* | myosin IB |
| rs6733725 | 2 | 203759315 | intron |  |  | [A/G] | Energy storage (kcal/d) | 5.70E-06 | 0.035 | A | 0.010 | *NBEAL1* | neurobeachin-like 1 |
| rs6733725 | 2 | 203759315 | intron |  |  | [A/G] | Fat mass deposition (kcal/d) | 6.05E-06 | 0.035 | A | 0.010 | *NBEAL1* | neurobeachin-like 1 |
| rs16839626 | 2 | 203797975 | flanking_3UTR |  |  | [T/C] | Energy storage (kcal/d) | 1.73E-07 | 0.045 | A | 0.006 | *NBEAL1* | neurobeachin-like 1 |
| rs16839626 | 2 | 203797975 | flanking_3UTR |  |  | [T/C] | Fat mass change (kg/y) | 3.54E-07 | 0.043 | A | 0.006 | *NBEAL1* | neurobeachin-like 1 |
| rs16839626 | 2 | 203797975 | flanking_3UTR |  |  | [T/C] | Fat mass deposition (kcal/d) | 2.02E-07 | 0.045 | A | 0.006 | *NBEAL1* | neurobeachin-like 1 |
| rs16839626 | 2 | 203797975 | flanking_3UTR |  |  | [T/C] | Weight change (kg/y) | 3.01E-06 | 0.035 | A | 0.006 | *NBEAL1* | neurobeachin-like 1 |
| rs16840760 | 2 | 204691799 | flanking_3UTR |  |  | [A/G] | AST/ALT | 1.24E-06 | 0.032 | G | 0.006 | *ICOS* | inducible T-cell co-stimulator |
| rs9789347 | 2 | 214546758 | intron |  |  | [T/G] | Total glutathione (µmol/L) | 7.54E-06 | 0.028 | A | 0.326 | *SPAG16* | sperm associated antigen 16 |
| rs16851771 | 2 | 214896864 | intron |  |  | [T/C] | Urinary free dopamine (nmol/d) | 2.41E-06 | 0.036 | G | 0.061 | *SPAG16* | sperm associated antigen 16 |
| rs13403276 | 2 | 218789542 | flanking_5UTR |  |  | [A/G] | Diet carbohydrate (g/d) | 6.84E-06 | 0.033 | G | 0.027 | *ARPC2* | actin related protein 2/3 complex, subunit 2, 34kD |
| rs13403276 | 2 | 218789542 | flanking_5UTR |  |  | [A/G] | Energy intake (kcal/d) | 2.95E-06 | 0.032 | G | 0.027 | *ARPC2* | actin related protein 2/3 complex, subunit 2, 34kD |
| rs6733051 | 2 | 218792778 | intron |  |  | [T/C] | BMR RQ | 1.87E-06 | 0.041 | A | 0.010 | *ARPC2* | actin related protein 2/3 complex, subunit 2, 34kDa |
| rs359980 | 2 | 219537450 | flanking_3UTR |  |  | [A/G] | TBF-ß1 (pg/mL) | 7.19E-06 | 0.027 | G | 0.054 | *CDK5R2* | cyclin-dependent kinase 5, regulatory subunit 2 (p39) |
| rs7601486 | 2 | 224053077 | flanking_3UTR |  |  | [T/C] | IL-6 (pg/mL) | 9.54E-06 | 0.032 | A | 0.181 | *SCG2* | secretogranin II |
| rs11691652 | 2 | 228293142 | flanking_5UTR |  |  | [T/G] | Cortisol (ng/mL) | 7.76E-06 | 0.031 | C | 0.189 | *SLC19A3* | solute carrier family 19, member 3 |
| rs9784019 | 2 | 230743819 | intron |  |  | [A/G] | BMR RQ | 9.72E-06 | 0.037 | G | 0.088 | *SP110* | SP110 nuclear body protein |
| rs13010639 | 2 | 230743989 | intron |  |  | [T/C] | BMR RQ | 5.95E-06 | 0.039 | A | 0.085 | *SP110* | SP110 nuclear body protein |
| rs13018234 | 2 | 230745110 | coding | SYNON | C577C(NP_004500.2) | [A/G] | BMR RQ | 9.72E-06 | 0.037 | A | 0.087 | *SP110* | SP110 nuclear body protein |
| rs13025520 | 2 | 230746273 | intron |  |  | [T/C] | BMR RQ | 9.72E-06 | 0.037 | A | 0.088 | *SP110* | SP110 nuclear body protein |
| rs1991705 | 2 | 235793690 | flanking_5UTR |  |  | [T/C] | Total glutathione (µmol/L) | 6.85E-06 | 0.026 | A | 0.175 | *LOC642692* | centrosomal protein 19kDa pseudogene |
| rs2292873 | 2 | 238164183 | UTR |  |  | [A/G] | Triglycerides (mg/dL) | 7.92E-06 | 0.034 | A | 0.061 | *RAB17* | RAB17, member RAS oncogene family |
| rs10185142 | 2 | 238487070 | flanking_3UTR |  |  | [T/C] | Diastolic blood pressure (mmHg) | 8.30E-06 | 0.029 | A | 0.216 | *RAMP1* | receptor (G protein-coupled) activity modifying protein 1 |
| rs4852124 | 2 | 240344959 | flanking_5UTR |  |  | [A/G] | Fat free mass change (kg/y) | 7.41E-06 | 0.034 | G | 0.453 | *LOC150935* | uncharacterized LOC150935 |
| rs10207060 | 2 | 240352226 | intron |  |  | [T/G] | Fat free mass change (kg/y) | 5.51E-06 | 0.036 | A | 0.450 | *LOC150935* | uncharacterized LOC150935 |
| rs10207060 | 2 | 240352226 | intron |  |  | [T/G] | Protein deposition (kcal/d) | 9.86E-06 | 0.033 | A | 0.450 | *LOC150935* | uncharacterized LOC150935 |
| rs10207060 | 2 | 240352226 | intron |  |  | [T/G] | Protein (kg/y) | 9.86E-06 | 0.033 | A | 0.450 | *LOC150935* | uncharacterized LOC150935 |
| rs4852140 | 2 | 240410655 | flanking_3UTR |  |  | [T/C] | Dinner intake (kcal) | 8.34E-06 | 0.041 | A | 0.328 | *LOC150935* | uncharacterized LOC150935 |
| rs12478296 | 2 | 242697433 | intron |  |  | [A/G] | Vitamin B12 (pmol/L) | 7.87E-06 | 0.031 | A | 0.446 | *LOC728323* | uncharacterized LOC728323 |
| rs892295 | 3 | 372553 | intron |  |  | [T/C] | ALT (U/L) | 3.75E-06 | 0.030 | A | 0.033 | *CHL1* | cell adhesion molecule with homology to L1CAM |
| rs892295 | 3 | 372553 | intron |  |  | [T/C] | ALT/AST | 1.40E-06 | 0.030 | A | 0.033 | *CHL1* | cell adhesion molecule with homology to L1CAM |
| rs2306870 | 3 | 4674776 | intron |  |  | [T/C] | IL-6 (pg/mL) | 5.92E-06 | 0.031 | G | 0.123 | *ITPR1* | inositol 1,4,5-trisphosphate receptor, type 1 |
| rs2306868 | 3 | 4674967 | intron |  |  | [C/G] | IL-6 (pg/mL) | 5.92E-06 | 0.031 | C | 0.123 | *ITPR1* | inositol 1,4,5-trisphosphate receptor, type 1 |
| rs7632000 | 3 | 4678937 | intron |  |  | [T/C] | IL-6 (pg/mL) | 6.70E-07 | 0.034 | A | 0.105 | *ITPR1* | inositol 1,4,5-trisphosphate receptor, type 1 |
| rs339664 | 3 | 42783497 | flanking_5UTR |  |  | [T/C] | Eotaxin (pg/mL) | 8.73E-06 | 0.025 | A | 0.297 | *CCDC13* | coiled-coil domain containing 13 |
| rs11717346 | 3 | 42803550 | intron |  |  | [T/C] | Eotaxin (pg/mL) | 9.38E-06 | 0.025 | A | 0.294 | *HIGD1A* | HIG1 hypoxia inducible domain family, member 1A |
| rs11129979 | 3 | 42865735 | intron |  |  | [T/C] | Eotaxin (pg/mL) | 4.07E-06 | 0.023 | A | 0.335 | *CCBP2* | Chemokine-binding protein 2 |
| rs9815043 | 3 | 42871643 | intron |  |  | [T/C] | Eotaxin (pg/mL) | 7.54E-07 | 0.027 | A | 0.348 | *CCBP2* | Chemokine-binding protein 2 |
| rs2228467 | 3 | 42881120 | coding | NONSYN | V41A(NP_001287.2) | [A/G] | Eotaxin (pg/mL) | 1.89E-06 | 0.033 | G | 0.038 | *CCBP2* | Chemokine-binding protein 2 |
| rs2228468 | 3 | 42882116 | coding | NONSYN | Y373S(NP_001287.2) | [T/G] | Eotaxin (pg/mL) | 2.44E-07 | 0.029 | A | 0.351 | *CCBP2* | Chemokine-binding protein 2 |
| rs1366046 | 3 | 42883779 | 3UTR |  |  | [T/G] | Eotaxin (pg/mL) | 2.44E-07 | 0.029 | A | 0.351 | *CCBP2* | Chemokine-binding protein 2 |
| rs3919627 | 3 | 42884164 | flanking_3UTR |  |  | [A/G] | Eotaxin (pg/mL) | 1.57E-07 | 0.031 | G | 0.353 | *CCBP2* | Chemokine-binding protein 2 |
| rs11715464 | 3 | 42884691 | flanking_3UTR |  |  | [T/C] | Eotaxin (pg/mL) | 9.14E-07 | 0.029 | G | 0.182 | *CCBP2* | chemokine binding protein 2 |
| rs7612912 | 3 | 42885625 | flanking_3UTR |  |  | [A/G] | Eotaxin (pg/mL) | 3.73E-07 | 0.029 | A | 0.348 | *CCBP2* | chemokine binding protein 2 |
| rs11919400 | 3 | 42980983 | flanking_5UTR |  |  | [T/C] | Eotaxin (pg/mL) | 8.83E-06 | 0.027 | G | 0.140 | *FAM198A* | family with sequence similarity 198, member A |
| rs6767019 | 3 | 43272693 | flanking_5UTR |  |  | [A/C] | Eotaxin (pg/mL) | 9.03E-06 | 0.026 | C | 0.085 | *SNRK* | SNF related kinase |
| rs17473118 | 3 | 43418538 | intron |  |  | [T/C] | Eotaxin (pg/mL) | 2.33E-07 | 0.034 | A | 0.069 | *ANO10* | Anoctamin 10 |
| rs7650267 | 3 | 43442899 | intron |  |  | [T/C] | Eotaxin (pg/mL) | 1.54E-07 | 0.037 | G | 0.093 | *ANO10* | Anoctamin 10 |
| rs12636651 | 3 | 46257395 | flanking_5UTR |  |  | [T/C] | MCP-1 (pg/mL) | 6.60E-09 | 0.050 | G | 0.403 | *CCR3* | chemokine (C-C motif) receptor 3 |
| rs7652290 | 3 | 46274769 | intron |  |  | [T/C] | MCP-1 (pg/mL) | 3.25E-07 | 0.038 | G | 0.437 | *CCR3* | chemokine (C-C motif) receptor 3 |
| rs4413345 | 3 | 46285925 | flanking_3UTR |  |  | [A/G] | MCP-1 (pg/mL) | 1.86E-06 | 0.033 | G | 0.405 | *CCR3* | chemokine (C-C motif) receptor 3 |
| rs7645716 | 3 | 46311785 | flanking_3UTR |  |  | [A/G] | MCP-1 (pg/mL) | 9.56E-08 | 0.041 | A | 0.381 | *CCR3* | chemokine (C-C motif) receptor 3 |
| rs10510751 | 3 | 46321029 | flanking_3UTR |  |  | [T/G] | MCP-1 (pg/mL) | 3.74E-06 | 0.032 | C | 0.239 | *CCR3* | chemokine (C-C motif) receptor 3 |
| rs1799864 | 3 | 46374212 | coding |  |  | [T/C] | MCP-1 (pg/mL) | 2.82E-06 | 0.033 | A | 0.244 | *CCR2* | chemokine (C-C motif) receptor 2 |
| rs3135940 | 3 | 48482579 | UTR |  |  | [A/G] | IGFBP-3 (ng/mL) | 5.80E-06 | 0.027 | A | 0.045 | *TREX1* | three prime repair exonuclease 1 |
| rs13100723 | 3 | 56773535 | intron |  |  | [A/G] | Glucose (mg/dL) | 1.35E-06 | 0.031 | A | 0.119 | *ARHGEF3* | Rho guanine nucleotide exchange factor (GEF) 3 |
| rs6768930 | 3 | 57754876 | intron |  |  | [A/G] | Gestational age (wk) | 5.34E-06 | 0.049 | G | 0.396 | *SLMAP* | sarcolemma associated protein |
| rs4522784 | 3 | 84284893 | flanking_5UTR |  |  | [T/G] | IGF-1 free (ng/mL) | 6.83E-06 | 0.036 | C | 0.010 |  |  |
| rs17023661 | 3 | 87051199 | flanking_3UTR |  |  | [T/C] | IL-6 (pg/mL) | 1.49E-06 | 0.040 | A | 0.221 | *VGLL3* | vestigial like 3 (Drosophila) |
| rs6796026 | 3 | 97811517 | flanking_5UTR |  |  | [A/G] | IGF-1 free (ng/mL) | 7.94E-06 | 0.033 | A | 0.008 | *RPL18AP8* | ribosomal protein L18a pseudogene 8 |
| rs7650621 | 3 | 98164952 | intron |  |  | [T/G] | IGF-1 free (ng/mL) | 1.79E-06 | 0.037 | C | 0.006 | *EPHA6* | EPH receptor A6 |
| rs9870146 | 3 | 118638945 | flanking_3UTR |  |  | [T/C] | Eotaxin (pg/mL) | 8.94E-06 | 0.030 | G | 0.497 | *PTMAP8* | prothymosin, alpha pseudogene 8 |
| rs10511378 | 3 | 119645313 | flanking_3UTR |  |  | [T/C] | IGFBP-1 (ng/mL) | 1.32E-06 | 0.035 | G | 0.112 | *IGSF11* | immunoglobulin superfamily, member 11 |
| rs9289146 | 3 | 121719019 | flanking_5UTR |  |  | [A/G] | RQmax | 7.02E-06 | 0.038 | A | 0.090 | *FSTL1* | follistatin-like 1 |
| rs13077101 | 3 | 121908021 | intron |  |  | [A/G] | ALT/AST | 1.08E-06 | 0.030 | G | 0.177 | *RABL3* | member of RAS oncogene family-like 3 |
| rs13077101 | 3 | 121908021 | intron |  |  | [A/G] | AST/ALT | 2.02E-07 | 0.034 | G | 0.177 | *RABL3* | RAB, member of RAS oncogene family-like 3 |
| rs373188 | 3 | 121994180 | flanking_3UTR |  |  | [T/G] | ALT/AST | 4.92E-06 | 0.026 | C | 0.365 | *GTF2E1* | general transcription factor IIE, polypeptide 1, alpha 56kDa |
| rs373188 | 3 | 121994180 | flanking_3UTR |  |  | [T/G] | AST/ALT | 6.22E-06 | 0.025 | C | 0.365 | *GTF2E1* | general transcription factor IIE, polypeptide 1, alpha 56kDa |
| rs13317079 | 3 | 124485988 | flanking_3UTR |  |  | [T/G] | MCP-1 (pg/mL) | 2.20E-06 | 0.037 | A | 0.042 | *ADCY5* | adenylate cyclase 5 |
| rs1044826 | 3 | 140555701 | intron |  |  | [A/G] | HOMA-IR | 8.15E-06 | 0.032 | G | 0.412 | *MRPS22* | mitochondrial ribosomal protein S22 |
| rs1044826 | 3 | 140555701 | intron |  |  | [A/G] | Insulin (µU/mL) | 8.93E-06 | 0.031 | G | 0.412 | *MRPS22* | mitochondrial ribosomal protein S22 |
| rs12632001 | 3 | 147332598 | intron |  |  | [T/C] | NEFA (mmol/L) | 4.75E-06 | 0.028 | G | 0.311 | *PLOD2* | procollagen-lysine, 2-oxoglutarate 5-dioxygenase 2 |
| rs1913185 | 3 | 147371147 | flanking_5UTR |  |  | [A/C] | NEFA (mmol/L) | 2.33E-07 | 0.041 | C | 0.249 | *PLOD2* | procollagen-lysine, 2-oxoglutarate 5-dioxygenase 2 |
| rs11712263 | 3 | 173868387 | intron |  |  | [A/G] | HDL (mg/dL) | 7.84E-06 | 0.030 | A | 0.135 | *NCEH1* | neutral cholesterol ester hydrolase 1 |
| rs7646507 | 3 | 173959113 | intron |  |  | [T/C] | TG/HDLC (mmol/L) | 7.73E-06 | 0.029 | G | 0.084 | *ECT2* | epithelial cell transforming sequence 2 oncogene |
| rs10936797 | 3 | 175676937 | intron |  |  | [T/G] | Total T4 (µg/dL) | 1.67E-06 | 0.030 | C | 0.296 |  |  |
| rs6806377 | 3 | 185325829 | flanking_3UTR |  |  | [A/G] | MCP-1 (pg/mL) | 1.08E-06 | 0.035 | A | 0.487 | *HSP90AA5P* | heat shock protein 90kDa alpha (cytosolic), class A member 5, pseudogene |
| rs10937355 | 3 | 189772391 | intron |  |  | [T/C] | Eotaxin (pg/mL) | 8.56E-06 | 0.027 | A | 0.367 | *LPP* | LIM domain containing preferred translocation partner in lipoma |
| rs6774852 | 3 | 197562824 | flanking_3UTR |  |  | [A/G] | Weight z-score (SD) | 7.42E-06 | 0.028 | G | 0.195 | *UBXD7* | UBX domain protein 7 |
| rs62296075 | 4 | 1029215 | flanking_3UTR |  |  | [A/G] | Sedentary activity (min/d) | 4.11E-06 | 0.029 | G | 0.023 | *FGFRL1* | fibroblast growth factor receptor-like 1 |
| rs62294688 | 4 | 1033718 | flanking_3UTR |  |  | [T/G] | Sedentary activity (min/d) | 6.98E-06 | 0.026 | C | 0.020 | *FGFRL1* | fibroblast growth factor receptor-like 1 |
| rs11247983 | 4 | 1271951 | flanking_5UTR |  |  | [C/G] | HRmax (bpm) | 5.41E-06 | 0.043 | C | 0.350 | *MAEA* | macrophage erythroblast attacher |
| rs11727167 | 4 | 1275521 | intron |  |  | [T/C] | HRmax (bpm) | 5.41E-06 | 0.043 | G | 0.350 | *MAEA* | macrophage erythroblast attacher |
| rs13108904 | 4 | 1281113 | intron |  |  | [A/C] | HRmax (bpm) | 2.41E-06 | 0.044 | A | 0.357 | *MAEA* | macrophage erythroblast attacher |
| rs2293635 | 4 | 1281791 | intron |  |  | [A/G] | HRmax (bpm) | 5.41E-06 | 0.043 | G | 0.350 | *MAEA* | macrophage erythroblast attacher |
| rs11933855 | 4 | 1305183 | intron |  |  | [A/G] | HRmax (bpm) | 7.20E-06 | 0.041 | A | 0.434 | *MAEA* | macrophage erythroblast attacher |
| rs11247998 | 4 | 1353602 | intron |  |  | [T/C] | HRmax (bpm) | 9.05E-06 | 0.040 | G | 0.353 | *KIAA1530* | KIAA1530 |
| rs4974559 | 4 | 1370848 | 3UTR |  |  | [A/G] | HRmax (bpm) | 8.53E-06 | 0.045 | G | 0.461 | *KIAA1530* | KIAA1530 |
| rs3822262 | 4 | 6748667 | intron |  |  | [T/G] | Total energy expenditure RQ | 3.06E-06 | 0.033 | C | 0.363 | *S100P* | S100P S100 calcium binding protein P |
| rs7694661 | 4 | 7474652 | flanking_3UTR |  |  | [A/C] | Fat mass change (kg/y) | 2.66E-06 | 0.038 | A | 0.406 | *SORCS2* | sortilin-related VPS10 domain containing receptor 2 |
| rs7694661 | 4 | 7474652 | flanking_3UTR |  |  | [A/C] | Fat mass deposition (kcal/d) | 8.73E-06 | 0.033 | A | 0.406 | *SORCS2* | sortilin-related VPS10 domain containing receptor 2 |
| rs7671189 | 4 | 11283759 | flanking_5UTR |  |  | [A/G] | Folate (nmol/L) | 3.28E-06 | 0.029 | A | 0.375 |  |  |
| rs7665957 | 4 | 12107985 | flanking_3UTR |  |  | [A/G] | Sleep duration (min/d) | 4.55E-07 | 0.013 | A | 0.006 |  |  |
| rs62295889 | 4 | 12109319 | flanking_3UTR |  |  | [A/G] | Urinary free epinephrine (nmol/d) | 5.50E-06 | 0.031 | G | 0.026 | *RAB28* | RAB28, member RAS oncogene family |
| rs11935262 | 4 | 12163809 | flanking_5UTR |  |  | [T/G] | Sleep duration (min/d) | 5.31E-06 | 0.013 | C | 0.007 | *HSP90AB2P* | heat shock protein 90kDa alpha (cytosolic), class B member 2, pseudogene |
| rs6844339 | 4 | 13753306 | flanking_3UTR |  |  | [T/C] | RANTES (pg/mL) | 6.30E-06 | 0.026 | G | 0.328 | *LOC152742* | uncharacterized LOC152742 |
| rs9918079 | 4 | 15154508 | intron |  |  | [T/C] | LDL (mg/dL) | 5.65E-06 | 0.035 | A | 0.115 | *CC2D2A* | coiled-coil and C2 domain containing 2A |
| rs16871289 | 4 | 21120481 | intron |  |  | [T/C] | CRP (ng/mL) | 9.21E-06 | 0.027 | A | 0.017 | *KCNIP4* | Kv channel interacting protein 4 |
| rs1604805 | 4 | 21180185 | flanking_5UTR |  |  | [A/G] | NEFA (mmol/L) | 8.46E-06 | 0.021 | G | 0.058 | *KCNIP4* | Kv channel interacting protein 4 |
| rs16872248 | 4 | 21630598 | flanking_5UTR |  |  | [T/C] | Fat mass (%) | 6.26E-06 | 0.027 | A | 0.047 | *UM9(5) -- KCNIP4-IT1* | KCNIP4 intronic transcript 1 (non-protein coding) |
| rs7654585 | 4 | 25551849 | flanking_3UTR |  |  | [T/C] | Waist circumference change (cm/y) | 2.24E-07 | 0.039 | A | 0.374 | *C4orf52* | chromosome 4 open reading frame 52 |
| rs907499 | 4 | 25746117 | flanking_3UTR |  |  | [A/G] | Snack intake (kcal) | 4.32E-06 | 0.045 | G | 0.016 | *LOC645481* | zinc finger protein 248 pseudogene |
| rs907499 | 4 | 25746117 | flanking_3UTR |  |  | [A/G] | Snack intake (kcal) | 5.99E-06 | 0.041 | G | 0.016 | *LOC645481* | zinc finger protein 248 pseudogene |
| rs6822297 | 4 | 26610093 | intron |  |  | [A/G] | Diet carbohydrate (%energy) | 8.35E-06 | 0.023 | G | 0.405 | *STIM2* | stromal interaction molecule 2 |
| rs12499826 | 4 | 29672487 | flanking_5UTR |  |  | [A/G] | Energy balance (kcal/d) | 8.26E-06 | 0.035 | A | 0.129 | *PCDH7* | protocadherin 7 |
| rs6818288 | 4 | 29677311 | flanking_5UTR |  |  | [A/C] | Energy balance (kcal/d) | 5.61E-06 | 0.036 | C | 0.124 | *PCDH7* | protocadherin 7 |
| rs6834483 | 4 | 35659506 | flanking_3UTR |  |  | [T/C] | Light activity (%awake time) | 5.60E-06 | 0.025 | G | 0.017 | *CENTD1* | ArfGAP with RhoGAP domain, ankyrin repeat and PH domain 2 |
| rs6834483 | 4 | 35659506 | flanking_3UTR |  |  | [T/C] | Sedentary activity (%awake time) | 5.89E-07 | 0.032 | G | 0.017 | *CENTD1* | ArfGAP with RhoGAP domain, ankyrin repeat and PH domain 2 |
| rs6834483 | 4 | 35659506 | flanking_3UTR |  |  | [T/C] | Sedentary activity (min/d) | 2.73E-06 | 0.029 | G | 0.017 | *CENTD1* | ArfGAP with RhoGAP domain, ankyrin repeat and PH domain 2 |
| rs4615179 | 4 | 38141866 | flanking_3UTR |  |  | [A/G] | Urinary free epinephrine (nmol/d) | 7.53E-06 | 0.021 | A | 0.098 | *FLJ13197* | uncharacterized FLJ13197 |
| rs9997524 | 4 | 42746943 | flanking_3UTR |  |  | [T/G] | Head circumference (cm) | 6.97E-06 | 0.023 | C | 0.005 | *GRXCR1* | glutaredoxin, cysteine rich 1 |
| rs6289 | 4 | 47103466 | coding | SYNON | T282T(NP_000803.1) | [A/G] | Weight change (kg/y) | 5.39E-06 | 0.029 | G | 0.169 | *GABRB1* | gamma-aminobutyric acid (GABA) A receptor, beta 1 |
| rs17083650 | 4 | 63898361 | flanking_5UTR |  |  | [A/G] | TSH (µIU/mL) | 9.93E-06 | 0.032 | A | 0.201 | *LOC644578* | hypothetical protein LOC644578 |
| rs2009314 | 4 | 66983397 | flanking_3UTR |  |  | [T/C] | Dinner intake (kcal) | 5.31E-06 | 0.033 | G | 0.296 | *LOC728048* | interferon induced transmembrane protein pseudogene |
| rs939207 | 4 | 68850266 | flanking_3UTR |  |  | [T/C] | Hip circumference change (cm/y) | 3.42E-06 | 0.032 | A | 0.142 | *YTHDC1* | YTH domain containing 1 |
| rs4356975 | 4 | 70007052 | intron |  |  | [T/C] | Gestational age (wk) | 1.86E-07 | 0.067 | A | 0.295 | *UGT2B7* | UDP glucuronosyltransferase 2 family, polypeptide B7 |
| rs17004924 | 4 | 81664331 | intron |  |  | [T/C] | Vitamin B12 (pmol/L) | 7.36E-06 | 0.022 | A | 0.201 | *C4orf22* | chromosome 4 open reading frame 22 |
| rs1385890 | 4 | 81754417 | intron |  |  | [T/C] | Vitamin B12 (pmol/L) | 4.32E-06 | 0.022 | G | 0.193 | *C4orf22* | chromosome 4 open reading frame 22 |
| rs13136169 | 4 | 81771902 | intron |  |  | [A/G] | Vitamin B12 (pmol/L) | 5.98E-06 | 0.022 | G | 0.200 | *C4orf22* | chromosome 4 open reading frame 22 |
| rs4693646 | 4 | 84940838 | flanking_3UTR |  |  | [A/G] | IGF-1 free (ng/mL) | 8.79E-06 | 0.027 | A | 0.407 | *MAG1 -- AGPAT9* | 1-acylglycerol-3-phosphate O-acyltransferase 9 |
| rs10516757 | 4 | 86923821 | intron |  |  | [A/G] | Amylin (pM) | 8.47E-06 | 0.026 | G | 0.185 | *ARHGAP24* | Rho GTPase activating protein 24 |
| rs7687906 | 4 | 86934336 | intron |  |  | [A/C] | Amylin (pM) | 5.02E-06 | 0.029 | C | 0.217 | *ARHGAP24* | Rho GTPase activating protein 24 |
| rs7666831 | 4 | 93540050 | intron |  |  | [A/G] | Free T3 (pg/mL) | 7.74E-06 | 0.016 | A | 0.004 | *GRID2* | glutamate receptor, ionotropic, delta 2 |
| rs11097470 | 4 | 96686526 | intron |  |  | [A/G] | Energy balance (kcal/d) | 5.14E-06 | 0.037 | A | 0.072 | *UNC5C* | unc-5 homolog C (C. elegans) |
| rs13132688 | 4 | 100230194 | flanking_5UTR |  |  | [A/G] | Diet carbohydrate (%energy) | 9.06E-06 | 0.024 | A | 0.006 | *ADH5* | alcohol dehydrogenase 5 (class III), chi polypeptide |
| rs10012953 | 4 | 111183811 | flanking_3UTR |  |  | [T/C] | Adiponectin (ng/mL) | 3.15E-06 | 0.028 | G | 0.164 | *ELOVL6* | ELOVL fatty acid elongase 6 |
| rs10018902 | 4 | 122081951 | flanking_5UTR |  |  | [T/C] | Weight z-score change (SD/y) | 1.41E-06 | 0.038 | A | 0.008 | *PRDM5* | PR domain containing 5 |
| rs6855125 | 4 | 122108503 | flanking_5UTR |  |  | [A/G] | Weight z-score change (SD/y) | 6.54E-06 | 0.031 | A | 0.038 | *PRDM5* | PR domain containing 5 |
| rs17010396 | 4 | 127026779 | flanking_5UTR |  |  | [A/C] | Diet protein (%energy) | 9.69E-06 | 0.027 | C | 0.133 | *LOC645841* | chromosome 7 open reading frame 42 pseudogene |
| rs1443170 | 4 | 128540358 | flanking_5UTR |  |  | [T/C] | Estradiol (pg/mL) | 9.02E-06 | 0.025 | G | 0.003 | *LOC729424* | uncharacterized LOC729424 |
| rs724950 | 4 | 128561570 | flanking_5UTR |  |  | [T/C] | RQmax | 7.52E-06 | 0.039 | A | 0.365 | *LOC729424* | uncharacterized LOC729424 |
| rs17049735 | 4 | 138795453 | flanking_5UTR |  |  | [A/G] | Snack intake (kcal) | 1.23E-06 | 0.047 | A | 0.149 | *PCDH18* | protocadherin 18 |
| rs17049735 | 4 | 138795453 | flanking_5UTR |  |  | [A/G] | Snack intake (kcal) | 2.70E-06 | 0.045 | A | 0.149 | *PCDH18* | protocadherin 18 |
| rs17049741 | 4 | 138802161 | flanking_5UTR |  |  | [A/G] | Snack intake (kcal) | 8.07E-07 | 0.048 | A | 0.150 | *PCDH18* | protocadherin 18 |
| rs17049741 | 4 | 138802161 | flanking_5UTR |  |  | [A/G] | Snack intake (kcal) | 1.71E-06 | 0.046 | A | 0.150 | *PCDH18* | protocadherin 18 |
| rs1429107 | 4 | 148541837 | flanking_5UTR |  |  | [A/G] | Total homocysteine (µmol/L) | 7.54E-06 | 0.022 | A | 0.086 | *EDNRA* | endothelin receptor type A |
| rs11731298 | 4 | 148831938 | flanking_5UTR |  |  | [T/C] | Estradiol (pg/mL) | 8.92E-06 | 0.027 | A | 0.017 | *PRMT10* | protein arginine methyltransferase 10 (putative) |
| rs77708442 | 4 | 154115610 | coding |  |  | [T/C] | IGFBP-3 (ng/mL) | 7.55E-06 | 0.026 | A | 0.032 | *FHDC1* | FH2 domain containing 1 |
| rs2404916 | 4 | 155214913 | flanking_3UTR |  |  | [T/C] | Total homocysteine (µmol/L) | 8.90E-06 | 0.023 | G | 0.334 | *DCHS2* | dachsous 2 (Drosophila) |
| rs3733418 | 4 | 166097785 | coding | NONSYN | H54R(NP_694572.1) | [T/C] | MCP-1 (pg/mL) | 9.29E-06 | 0.021 | G | 0.197 | *C4orf39* | chromosome 4 open reading frame 39 |
| rs17635075 | 4 | 167563389 | flanking_3UTR |  |  | [A/G] | Gestational age (wk) | 7.54E-06 | 0.052 | G | 0.076 | *TLL1* | tolloid-like 1 |
| rs9312517 | 4 | 168375955 | intron |  |  | [A/G] | LDL (mg/dL) | 2.77E-06 | 0.026 | G | 0.228 | *SPOCK3* | sparc/osteonectin, cwcv & kazal-like domains proteoglycan (testican)3 |
| rs9312517 | 4 | 168375955 | intron |  |  | [A/G] | Total cholesterol (mg/dL) | 7.95E-07 | 0.027 | G | 0.228 | *SPOCK3* | sparc/osteonectin, cwcv & kazal-like domains proteoglycan (testican)3 |
| rs1963569 | 4 | 169575095 | intron |  |  | [T/G] | Diet fat (%energy) | 8.12E-06 | 0.032 | A | 0.478 | *DDX60L* | DEAD (Asp-Glu-Ala-Asp) box polypeptide 60-like |
| rs11727767 | 4 | 174838623 | flanking_5UTR |  |  | [A/G] | Ghrelin (pg/100 µL) | 6.61E-06 | 0.030 | A | 0.447 | *MORF4* | mortality factor 4 |
| rs2333163 | 4 | 176507879 | flanking_5UTR |  |  | [A/G] | TBF-ß1 (pg/mL) | 5.16E-06 | 0.033 | G | 0.034 | *LOC391718* | tRNA splicing endonuclease 2 homolog pseudogene |
| rs11495908 | 4 | 180941912 | flanking_3UTR |  |  | [A/G] | ALT/AST | 4.15E-06 | 0.029 | G | 0.086 |  |  |
| rs11495908 | 4 | 180941912 | flanking_3UTR |  |  | [A/G] | AST/ALT | 1.91E-06 | 0.031 | G | 0.086 |  |  |
| rs2043893 | 4 | 182714380 | flanking_3UTR |  |  | [T/C] | Light activity (%awake time) | 2.87E-06 | 0.041 | G | 0.426 |  |  |
| rs1439283 | 4 | 182721763 | flanking_3UTR |  |  | [T/C] | Light activity (%awake time) | 1.25E-06 | 0.043 | A | 0.480 |  |  |
| rs6855088 | 4 | 182941545 | flanking_3UTR |  |  | [A/G] | AST (U/L) | 8.96E-07 | 0.032 | G | 0.221 |  |  |
| rs6552523 | 4 | 182957285 | flanking_3UTR |  |  | [A/G] | AST (U/L) | 1.90E-06 | 0.028 | G | 0.365 |  |  |
| rs13114435 | 4 | 184058656 | intron |  |  | [T/C] | Diet fat (%energy) | 2.19E-06 | 0.029 | G | 0.127 | *DCTD* | dCMP deaminase |
| rs10018215 | 4 | 186539400 | flanking_5UTR |  |  | [T/G] | Sleep duration (min/d) | 9.78E-06 | 0.027 | A | 0.294 | *LRP2BP* | LRP2 binding protein |
| rs4253238 | 4 | 187385381 | flanking_5UTR |  |  | [A/G] | IGF-1 free (ng/mL) | 1.29E-07 | 0.038 | G | 0.334 | *KLKB1* | kallikrein B, plasma (Fletcher factor) 1 |
| rs1912826 | 4 | 187386534 | intron |  |  | [T/C] | IGF-1 free (ng/mL) | 4.47E-07 | 0.034 | G | 0.342 | *KLKB1* | kallikrein B, plasma (Fletcher factor) 1 |
| rs3733402 | 4 | 187395028 | coding | NONSYN | S143T(NP_000883.2) | [A/G] | IGF-1 free (ng/mL) | 9.01E-08 | 0.037 | G | 0.337 | *KLKB1* | kallikrein B, plasma (Fletcher factor) 1 |
| rs34899763 | 4 | 187402427 | intron |  |  | [T/A] | IGF-1 free (ng/mL) | 5.44E-06 | 0.035 | T | 0.261 | *KLKB1* | kallikrein B, plasma (Fletcher factor) 1 |
| rs925453 | 4 | 187416204 | coding | SYNON | N587N(NP_000883.2) | [T/C] | IGF-1 free (ng/mL) | 4.35E-06 | 0.034 | A | 0.261 | *KLKB1* | kallikrein B, plasma (Fletcher factor) 1 |
| rs7686384 | 4 | 189821347 | flanking_5UTR |  |  | [A/G] | Cortisol (ng/mL) | 6.39E-06 | 0.013 | A | 0.129 | *RPL7AP27* | ribosomal protein L7a pseudogene 27 |
| rs11739663 | 5 | 647083 | flanking_5UTR |  |  | [A/G] | HDL (mg/dL) | 3.25E-06 | 0.034 | G | 0.209 | *CEP72* | centrosomal protein 72kDa |
| rs4956954 | 5 | 660388 | flanking_5UTR |  |  | [A/G] | HDL (mg/dL) | 7.90E-06 | 0.022 | G | 0.434 | *CEP72* | centrosomal protein 72kDa |
| rs1697952 | 5 | 667963 | intron |  |  | [A/G] | HDL (mg/dL) | 7.95E-06 | 0.021 | G | 0.428 | *CEP72* | centrosomal protein 72kDa |
| rs7726839 | 5 | 671586 | intron |  |  | [T/C] | HDL (mg/dL) | 1.37E-06 | 0.031 | G | 0.282 | *CEP72* | centrosomal protein 72kDa |
| rs2173226 | 5 | 2777362 | flanking_3UTR |  |  | [T/C] | Hip circumference change (cm/y) | 8.18E-06 | 0.033 | G | 0.342 |  |  |
| rs275437 | 5 | 6932978 | flanking_3UTR |  |  | [T/C] | Total cholesterol (mg/dL) | 2.64E-06 | 0.035 | A | 0.494 |  |  |
| rs4702435 | 5 | 7208612 | flanking_3UTR |  |  | [A/G] | Dinner intake, adj EER (kcal) | 4.78E-06 | 0.041 | G | 0.118 | *LOC442132* | golgin A6 family-like 1 pseudogene |
| rs11134338 | 5 | 9036721 | flanking_3UTR |  |  | [A/G] | ALT/AST | 8.30E-06 | 0.027 | A | 0.131 | *SEMA5A* | sema domain, seven thrombospondin repeats (type 1 and type 1-like) |
| rs433755 | 5 | 9527946 | intron |  |  | [T/G] | Total cysteine (µmol/L) | 5.43E-07 | 0.033 | C | 0.451 | *SEMA5A* | sema domain, seven thrombospondin repeats (type 1 and type 1-like) |
| rs4702718 | 5 | 10724040 | flanking_5UTR |  |  | [A/G] | Diet carbohydrate (%energy) | 2.84E-06 | 0.040 | G | 0.320 | *LOC645763* | intersectin 1 (SH3 domain protein) pseudogene |
| rs1447276 | 5 | 15417484 | flanking_5UTR |  |  | [A/G] | Fat mass (%) | 2.17E-06 | 0.028 | A | 0.116 | *LOC391741* | actin, beta-like 2 pseudogene |
| rs12659622 | 5 | 15671181 | intron |  |  | [A/G] | Total T3 (ng/dL) | 8.90E-07 | 0.032 | A | 0.063 | *FBXL7* | F-box and leucine-rich repeat protein 7 |
| rs17614462 | 5 | 16966855 | flanking_5UTR |  |  | [T/C] | IGFBP-1 (ng/mL) | 2.11E-06 | 0.032 | A | 0.045 | *MYO10* | myosin X |
| rs396045 | 5 | 25877370 | flanking_3UTR |  |  | [T/C] | Diet carbohydrate (%energy) | 6.87E-06 | 0.034 | A | 0.247 |  |  |
| rs7719829 | 5 | 31963074 | flanking_5UTR |  |  | [A/G] | Leptin (ng/mL) | 4.40E-06 | 0.031 | A | 0.395 | *PDZD2* | PDZ domain containing 2 |
| rs293748 | 5 | 36936600 | flanking_3UTR |  |  | [T/C] | Vigorous activity (%awake time) | 6.47E-06 | 0.033 | A | 0.158 | *NIPBL* | Nipped-B homolog (Drosophila) |
| rs1239344 | 5 | 38969506 | 3UTR |  |  | [T/C] | Urinary free epinephrine (nmol/d) | 1.16E-06 | 0.038 | A | 0.425 | *OSMR* | oncostatin M receptor |
| rs2043112 | 5 | 38991553 | coding | NONSYN | S837F(NP_689969.2) | [A/G] | Urinary free epinephrine (nmol/d) | 7.65E-07 | 0.041 | A | 0.389 | *RICTOR* | RPTOR independent companion of MTOR, complex 2 |
| rs13160161 | 5 | 39094113 | intron |  |  | [T/C] | Urinary free epinephrine (nmol/d) | 2.26E-06 | 0.036 | A | 0.382 | *RICTOR* | RPTOR independent companion of MTOR, complex 2 |
| rs6869095 | 5 | 39110453 | flanking_5UTR |  |  | [A/G] | Urinary free epinephrine (nmol/d) | 3.30E-06 | 0.033 | A | 0.415 | *RICTOR* | RPTOR independent companion of MTOR, complex 2 |
| rs6895953 | 5 | 39120228 | flanking_5UTR |  |  | [T/C] | Urinary free epinephrine (nmol/d) | 2.30E-06 | 0.036 | A | 0.389 | *RICTOR* | RPTOR independent companion of MTOR, complex 2 |
| rs13165383 | 5 | 39125099 | flanking_5UTR |  |  | [T/C] | Urinary free epinephrine (nmol/d) | 8.97E-06 | 0.031 | A | 0.428 | *RICTOR* | RPTOR independent companion of MTOR, complex 2 |
| rs36019094 | 5 | 40273131 | flanking_5UTR |  |  | [T/G] | Estradiol (pg/mL) | 1.25E-06 | 0.030 | A | 0.262 |  |  |
| rs2963826 | 5 | 59132680 | flanking_3UTR |  |  | [T/C] | IGF-1 bound (ng/mL) | 2.34E-06 | 0.029 | A | 0.325 | *PDE4D* | phosphodiesterase 4D, cAMP-specific |
| rs266590 | 5 | 64383377 | flanking_3UTR |  |  | [A/G] | Gestational age (wk) | 9.27E-06 | 0.046 | G | 0.485 | *SDCCAG10 -- CWC27* | CWC27 spliceosome-associated protein homolog (S. cerevisiae) |
| rs35131626 | 5 | 70870648 | coding | SYNON | I1972I(NP_060899.2) | [T/G] | Dinner intake, adj EER (kcal) | 8.52E-06 | 0.039 | A | 0.029 | *BDP1* | B double prime 1, subunit of RNA polymerase III transcription initiation factor IIIB |
| rs283610 | 5 | 73284268 | flanking_3UTR |  |  | [A/G] | CRP (ng/mL) | 7.23E-06 | 0.027 | G | 0.456 | *RGNEF* | 190 kDa guanine nucleotide exchange factor |
| rs1583686 | 5 | 85253625 | flanking_5UTR |  |  | [A/G] | ALT (U/L) | 1.33E-06 | 0.038 | A | 0.124 | *NBPF22P* | neuroblastoma breakpoint family, member 22, pseudogene |
| rs10514310 | 5 | 89057334 | flanking_5UTR |  |  | [T/C] | Waist:height | 6.68E-06 | 0.031 | G | 0.208 |  |  |
| rs10074525 | 5 | 90245580 | intron |  |  | [A/G] | Weight z-score (SD) | 9.09E-06 | 0.033 | G | 0.130 | *GPR98* | G protein-coupled receptor 98 |
| rs27540 | 5 | 92160029 | flanking_3UTR |  |  | [A/G] | Weight z-score (SD) | 3.18E-06 | 0.026 | A | 0.462 |  |  |
| rs17668565 | 5 | 92180335 | flanking_3UTR |  |  | [T/C] | BMI z-score (SD) | 9.31E-06 | 0.025 | G | 0.403 |  |  |
| rs17668565 | 5 | 92180335 | flanking_3UTR |  |  | [T/C] | Fat mass (%) | 1.98E-06 | 0.025 | G | 0.403 |  |  |
| rs17668565 | 5 | 92180335 | flanking_3UTR |  |  | [T/C] | Hip circumference (cm) | 6.46E-06 | 0.024 | G | 0.403 |  |  |
| rs17668565 | 5 | 92180335 | flanking_3UTR |  |  | [T/C] | Weight z-score (SD) | 4.65E-07 | 0.032 | G | 0.403 |  |  |
| rs13173682 | 5 | 92513299 | flanking_5UTR |  |  | [A/G] | Moderate activity (min/d) | 5.78E-06 | 0.026 | A | 0.231 | *POLD2P1* | polymerase (DNA directed), delta 2, regulatory subunit 50kDa pseudogene 1 |
| rs13173682 | 5 | 92513299 | flanking_5UTR |  |  | [A/G] | Moderate&vigorous activity (min/d) | 8.97E-06 | 0.024 | A | 0.231 | *POLD2P1* | polymerase (DNA directed), delta 2, regulatory subunit 50kDa pseudogene 1 |
| rs13189969 | 5 | 105325428 | flanking_3UTR |  |  | [A/C] | Fat mass (kg) | 8.42E-06 | 0.030 | A | 0.265 | *LOC345571* | RUN and FYVE domain containing 3 pseudogene |
| rs13189969 | 5 | 105325428 | flanking_3UTR |  |  | [A/C] | Trunk fat mass (kg) | 9.77E-06 | 0.030 | A | 0.265 | *LOC345571* | RUN and FYVE domain containing 3 pseudogene |
| rs6894797 | 5 | 105447412 | flanking_3UTR |  |  | [A/G] | Hip circumference change (cm/y) | 8.79E-06 | 0.029 | G | 0.047 | *LOC345571* | RUN and FYVE domain containing 3 pseudogene |
| rs11241130 | 5 | 110986681 | flanking_3UTR |  |  | [T/C] | Sleep duration (min/d) | 8.39E-06 | 0.035 | G | 0.129 | *NREP* | neuronal regeneration related protein homolog (rat) |
| rs400028 | 5 | 113877193 | flanking_3UTR |  |  | [A/G] | Adiponectin (ng/mL) | 4.69E-06 | 0.026 | G | 0.138 | *KCNN2* | potassium intermediate/small conductance calcium-activated channel, subfamily N, member 2 |
| rs17468244 | 5 | 124876805 | flanking_3UTR |  |  | [T/C] | Sedentary activity (min/d) | 8.30E-06 | 0.033 | G | 0.114 | *LOC644659* | high mobility group box 1 pseudogene |
| rs10077875 | 5 | 128750595 | flanking_5UTR |  |  | [A/G] | Energy intake (kcal/d) | 8.73E-06 | 0.029 | G | 0.448 | *ADAMTS19* | ADAM metallopeptidase with thrombospondin type 1 motif, 19 |
| rs606854 | 5 | 134600128 | flanking_3UTR |  |  | [T/C] | Total glutathione (µmol/L) | 5.64E-06 | 0.028 | A | 0.377 | *H2AFY* | H2A histone family, member Y |
| rs7734448 | 5 | 135242576 | intron |  |  | [A/G] | IL-6 (pg/mL) | 2.71E-06 | 0.030 | A | 0.005 | *SLC25A48* | solute carrier family 25, member 48 |
| rs3749779 | 5 | 140663075 | coding | NONSYN | V181G(NP_114153.1) | [A/C] | Vitamin B12 (pmol/L) | 7.00E-06 | 0.021 | C | 0.165 | *SLC25A2* | solute carrier family 25 (mitochondrial carrier; ornithine transporter) member 2 |
| rs6880903 | 5 | 144632606 | flanking_3UTR |  |  | [A/G] | Waist circumference change (cm/y) | 9.63E-06 | 0.026 | G | 0.006 | *PRELID2* | PRELI domain containing 2 |
| rs891992 | 5 | 147205707 | flanking_5UTR |  |  | [T/C] | AST (U/L) | 5.59E-06 | 0.026 | G | 0.234 | *SPINK1* | serine peptidase inhibitor, Kazal type 1 |
| rs10044242 | 5 | 148686492 | intron |  |  | [C/G] | Diet protein (g/d) | 9.02E-06 | 0.023 | G | 0.005 | *AFAP1L1* | actin filament associated protein 1-like 1 |
| rs32576 | 5 | 149193649 | intron |  |  | [A/G] | Estradiol (pg/mL) | 5.25E-06 | 0.027 | G | 0.391 | *PPARGC1B* | peroxisome proliferator-activated receptor gamma, coactivator 1 beta |
| rs4958456 | 5 | 149603558 | intron |  |  | [T/C] | Urinary free dopamine (nmol/d) | 4.61E-07 | 0.040 | A | 0.092 | *CAMK2A* | calcium/calmodulin-dependent protein kinase II alpha |
| rs979455 | 5 | 150574995 | intron |  |  | [A/G] | Dinner intake, adj EER (kcal) | 9.01E-06 | 0.038 | G | 0.201 | *CCDC69* | coiled-coil domain containing 69 |
| rs10065813 | 5 | 152920598 | intron |  |  | [A/G] | NEFA (mmol/L) | 8.10E-06 | 0.026 | A | 0.078 | *GRIA1* | glutamate receptor, ionotropic, AMPA 1 |
| rs1432723 | 5 | 155235160 | flanking_5UTR |  |  | [A/G] | Amylin (pM) | 8.74E-06 | 0.031 | G | 0.447 | *SGCD* | sarcoglycan, delta (35kDa dystrophin-associated glycoprotein) |
| rs13170526 | 5 | 158108247 | intron |  |  | [A/C] | Eotaxin (pg/mL) | 3.59E-06 | 0.029 | C | 0.039 | *EBF1* | early B-cell factor 1 |
| rs1106693 | 5 | 173285369 | intron |  |  | [A/G] | Height z-score (SD) | 2.11E-06 | 0.015 | G | 0.039 | *CPEB4* | cytoplasmic polyadenylation element binding protein 4 |
| rs251925 | 5 | 174714725 | flanking_3UTR |  |  | [T/C] | IGF-1 free (ng/mL) | 4.71E-06 | 0.028 | A | 0.285 | *DRD1* | dopamine receptor D1 |
| rs10039217 | 5 | 175111481 | flanking_5UTR |  |  | [T/C] | Sleep duration (min/d) | 2.98E-07 | 0.038 | G | 0.013 | *CPLX2* | complexin 2 |
| rs2913737 | 5 | 175782595 | flanking_5UTR |  |  | [T/C] | Triglycerides (mg/dL) | 8.31E-06 | 0.028 | A | 0.169 | *CLTB* | clathrin, light chain B |
| rs72813183 | 5 | 176667254 | flanking_3UTR |  |  | [T/C] | Free T3 (pg/mL) | 2.34E-06 | 0.045 | A | 0.062 | *MXD3* | MAX dimerization protein 3 |
| rs72813183 | 5 | 176667254 | flanking_3UTR |  |  | [T/C] | MCP-1 (pg/mL) | 1.52E-06 | 0.045 | A | 0.062 | *MXD3* | MAX dimerization protein 3 |
| rs57960711 | 5 | 176951868 | flanking_5UTR |  |  | [G/C] | Height change (cm/y) | 8.35E-06 | 0.031 | G | 0.033 | *TMED9* | transmembrane emp24 protein transport domain containing 9 |
| rs10070303 | 5 | 177772988 | intron |  |  | [A/G] | Fat mass change (kg/y) | 5.85E-06 | 0.034 | A | 0.249 | *COL23A1* | collagen, type XXIII, alpha 1 |
| rs11249616 | 5 | 178550301 | intron |  |  | [T/C] | Waist circumference change (cm/y) | 9.66E-06 | 0.029 | A | 0.146 | *ADAMTS2* | ADAM metallopeptidase with thrombospondin type 1 motif, 2 |
| rs6937692 | 6 | 1396454 | flanking_3UTR |  |  | [T/C] | Fat free mass (kg) | 9.50E-06 | 0.025 | G | 0.232 | *FOXF2* | forkhead box F2 |
| rs6937692 | 6 | 1396454 | flanking_3UTR |  |  | [T/C] | Lean body mass (kg) | 9.54E-06 | 0.025 | G | 0.232 | *FOXF2* | forkhead box F2 |
| rs3800131 | 6 | 1863261 | intron |  |  | [A/C] | Testosterone (ng/mL) | 9.35E-06 | 0.027 | A | 0.356 | *GMDS* | GDP-mannose 4,6-dehydratase |
| rs4959190 | 6 | 2506351 | flanking_3UTR |  |  | [T/C] | Urinary nitrogen (g/d) | 1.05E-06 | 0.043 | A | 0.436 | *C6orf195* | chromosome 6 open reading frame 195 |
| rs12195826 | 6 | 2510751 | flanking_3UTR |  |  | [A/G] | Urinary nitrogen (g/d) | 3.59E-07 | 0.045 | A | 0.407 | *C6orf195* | chromosome 6 open reading frame 195 |
| rs9378357 | 6 | 3239823 | intron |  |  | [T/G] | sICAM-1 (pg/mL) | 1.02E-06 | 0.031 | A | 0.185 | *SLC22A23* | solute carrier family 22, member 23 |
| rs3935358 | 6 | 3240189 | intron |  |  | [T/C] | sICAM-1 (pg/mL) | 6.83E-06 | 0.026 | G | 0.194 | *SLC22A23* | solute carrier family 22, member 23 |
| rs3935359 | 6 | 3240250 | intron |  |  | [A/C] | sICAM-1 (pg/mL) | 1.94E-06 | 0.034 | A | 0.161 | *SLC22A23* | solute carrier family 22, member 23 |
| rs17138098 | 6 | 4309140 | flanking_5UTR |  |  | [T/C] | Birth weight (kg) | 3.38E-06 | 0.030 | G | 0.197 | *PECI -- ECI2* | enoyl-CoA delta isomerase 2 |
| rs17138114 | 6 | 4324510 | flanking_5UTR |  |  | [A/G] | Birth weight (kg) | 1.27E-06 | 0.041 | G | 0.222 | *KU-MEL-3* | KU-MEL-3 |
| rs7739678 | 6 | 7597716 | flanking_3UTR |  |  | [A/G] | Fat mass change (kg/y) | 9.22E-06 | 0.030 | G | 0.089 | *SNRNP48* | small nuclear ribonucleoprotein 48kDa (U11/U12) |
| rs4959447 | 6 | 7599693 | flanking_3UTR |  |  | [T/C] | Fat mass change (kg/y) | 9.22E-06 | 0.030 | A | 0.089 | *SNRNP48* | small nuclear ribonucleoprotein 48kDa (U11/U12) |
| rs12198798 | 6 | 12618113 | flanking_5UTR |  |  | [A/G] | BMI z-score change (SD/y) | 1.43E-06 | 0.037 | G | 0.114 | *RPL15P3* | ribosomal protein L15 pseudogene 3 |
| rs7758616 | 6 | 14308126 | flanking_3UTR |  |  | [A/G] | NEFA (mmol/L) | 4.04E-06 | 0.025 | A | 0.018 | *CD83* | CD83 molecule |
| rs2274136 | 6 | 17741040 | coding | NONSYN | A827T(NP_005115.2) | [T/C] | LDL (mg/dL) | 3.08E-06 | 0.031 | G | 0.228 | *NUP153* | nucleoporin 153kDa |
| rs2274136 | 6 | 17741040 | coding | NONSYN | A827T(NP_005115.2) | [T/C] | Total cholesterol (mg/dL) | 5.21E-06 | 0.033 | G | 0.228 | *NUP153* | nucleoporin 153kDa |
| rs2457335 | 6 | 20217571 | intron |  |  | [T/C] | Head circumference (cm) | 1.64E-06 | 0.041 | G | 0.191 | *MBOAT1* | membrane bound O-acyltransferase domain containing 1 |
| rs1570155 | 6 | 20490579 | flanking_5UTR |  |  | [A/G] | Calorimeter activity (counts/d) | 4.09E-06 | 0.043 | A | 0.391 | *E2F3* | E2F transcription factor 3 |
| rs9465729 | 6 | 20508517 | flanking_5UTR |  |  | [A/G] | Calorimeter activity (counts/d) | 7.82E-06 | 0.041 | A | 0.390 | *E2F3* | E2F transcription factor 3 |
| rs9460521 | 6 | 20675520 | intron |  |  | [A/G] | Weight z-score (SD) | 7.78E-06 | 0.025 | A | 0.010 | *CDKAL1* | CDK5 regulatory subunit associated protein 1-like 1 |
| rs7356884 | 6 | 22861026 | flanking_5UTR |  |  | [A/G] | BMI z-score change (SD/y) | 8.32E-06 | 0.030 | G | 0.073 |  |  |
| rs13215567 | 6 | 32271726 | coding | SYNON | E1826E(NP_004548.3) | [A/G] | IGFBP-3 (ng/mL) | 9.99E-07 | 0.031 | A | 0.049 | *NOTCH4, GPSM3* | notch 4; G-protein signaling modulator 3 |
| rs9368714 | 6 | 32405319 | intron |  |  | [T/C] | Diet carbohydrate (g/d) | 2.76E-06 | 0.030 | A | 0.434 | *C6orf10* | chromosome 6 open reading frame 10 |
| rs9268301 | 6 | 32427615 | intron |  |  | [A/G] | Diet carbohydrate (g/d) | 1.95E-06 | 0.031 | A | 0.438 | *C6orf10* | chromosome 6 open reading frame 10 |
| rs9469457 | 6 | 33597860 | flanking_3UTR |  |  | [A/G] | Dinner intake, adj EER (kcal) | 9.31E-06 | 0.033 | A | 0.021 | *BAK1* | BCL2-antagonist/killer 1 |
| rs2296743 | 6 | 33848003 | 3UTR |  |  | [T/C] | Dinner intake, adj EER (kcal) | 8.08E-06 | 0.032 | A | 0.302 | *LEMD2* | LEMD2 LEM domain containing 2 |
| rs1535950 | 6 | 33893805 | flanking_5UTR |  |  | [A/G] | Dinner intake, adj EER (kcal) | 9.13E-06 | 0.030 | G | 0.330 | *MLN* | motilin |
| rs2005 | 6 | 35164421 | 3UTR |  |  | [A/G] | BMR RQ | 6.76E-06 | 0.034 | A | 0.089 | *ANKS1A* | ankyrin repeat and sterile alpha motif domain containing 1A |
| rs9366999 | 6 | 39310135 | flanking_5UTR |  |  | [A/G] | Waist circumference (cm) | 6.92E-06 | 0.026 | A | 0.409 | *KCNK5* | potassium channel, subfamily K, member 5 |
| rs9296295 | 6 | 39469832 | intron |  |  | [T/G] | TG/HDLC (mmol/L) | 6.36E-06 | 0.027 | A | 0.060 | *KIF6* | kinesin family member 6 |
| rs9296335 | 6 | 40548478 | flanking_5UTR |  |  | [A/G] | HDL (mg/dL) | 8.38E-06 | 0.028 | A | 0.033 | *LRFN2* | leucine rich repeat and fibronectin type III domain containing 2 |
| rs9296335 | 6 | 40548478 | flanking_5UTR |  |  | [A/G] | TG/HDLC (mmol/L) | 3.03E-06 | 0.026 | A | 0.033 | *LRFN2* | leucine rich repeat and fibronectin type III domain containing 2 |
| rs16895812 | 6 | 42603256 | flanking_5UTR |  |  | [T/C] | NEFA (mmol/L) | 2.55E-06 | 0.027 | G | 0.181 | *UBR2* | ubiquitin protein ligase E3 component n-recognin 2 |
| rs61743561 | 6 | 43261765 | coding |  |  | [T/C] | TBF-ß1 (pg/mL) | 9.31E-06 | 0.025 | A | 0.005 | *CUL9* | cullin 9 |
| rs1342371 | 6 | 44847927 | flanking_3UTR |  |  | [A/G] | IGF-1 bound (ng/mL) | 1.68E-06 | 0.025 | A | 0.088 | *SUPT3H* | suppressor of Ty 3 homolog (S. cerevisiae) |
| rs2770664 | 6 | 51106094 | flanking_3UTR |  |  | [A/C] | Vigorous activity (%awake time) | 9.53E-06 | 0.029 | A | 0.057 | *TFAP2B* | transcription factor AP-2 beta (activating enhancer binding protein 2 beta) |
| rs7762246 | 6 | 51207215 | flanking_3UTR |  |  | [A/G] | Vigorous activity (min/d) | 5.98E-06 | 0.028 | G | 0.110 | *LOC646517* | Wilms tumor 1 associated protein pseudogene |
| rs9474614 | 6 | 53622905 | flanking_3UTR |  |  | [T/C] | Total T4 (µg/dL) | 5.59E-06 | 0.027 | G | 0.224 | *KLHL31* | kelch-like 31 (Drosophila) |
| rs11754509 | 6 | 55581253 | flanking_5UTR |  |  | [T/C] | Urinary free dopamine: creatinine | 3.03E-07 | 0.027 | G | 0.013 | *HMGCLL1* | 3-hydroxymethyl-3-methylglutaryl-CoA lyase-like 1 |
| rs789942 | 6 | 63861147 | flanking_3UTR |  |  | [T/G] | Hip circumference change (cm/y) | 5.24E-06 | 0.038 | C | 0.412 | *GLULD1 -- LGSN* | lengsin, lens protein with glutamine synthetase domain |
| rs473861 | 6 | 71670372 | intron |  |  | [A/G] | ALT/AST | 8.93E-06 | 0.027 | A | 0.194 | *B3GAT2* | B3GAT2 beta-1,3-glucuronyltransferase 2 (glucuronosyltransferase S) |
| rs6922893 | 6 | 71716832 | intron |  |  | [T/C] | ALT/AST | 7.43E-06 | 0.027 | A | 0.219 | *B3GAT2* | B3GAT2 beta-1,3-glucuronyltransferase 2 (glucuronosyltransferase S) |
| rs6912405 | 6 | 74927951 | flanking_3UTR |  |  | [T/C] | Free T3 (pg/mL) | 8.45E-06 | 0.023 | G | 0.462 | *CD109* | CD109 molecule |
| rs6902257 | 6 | 81538073 | flanking_5UTR |  |  | [A/G] | Energy storage (kcal/d) | 9.82E-06 | 0.033 | A | 0.015 | *LOC442232* | golgin A6 family-like 1 pseudogene |
| rs6902257 | 6 | 81538073 | flanking_5UTR |  |  | [A/G] | Fat mass deposition (kcal/d) | 6.63E-06 | 0.035 | A | 0.015 | *LOC442232* | golgin A6 family-like 1 pseudogene |
| rs2273130 | 6 | 88275756 | intron |  |  | [A/G] | Fat mass change (kg/y) | 4.16E-06 | 0.037 | A | 0.027 | *SLC35A1* | solute carrier family 35 (CMP-sialic acid transporter), member A1 |
| rs2273130 | 6 | 88275756 | intron |  |  | [A/G] | Fat mass deposition (kcal/d) | 6.17E-06 | 0.035 | A | 0.027 | *SLC35A1* | solute carrier family 35 (CMP-sialic acid transporter), member A1 |
| rs6900057 | 6 | 88330688 | intron |  |  | [A/C] | Fat mass change (kg/y) | 9.15E-06 | 0.034 | C | 0.035 | *RARS2* | arginyl-tRNA synthetase 2, mitochondrial |
| rs6900057 | 6 | 88330688 | intron |  |  | [A/C] | Fat mass deposition (kcal/d) | 7.59E-06 | 0.034 | C | 0.035 | *RARS2* | arginyl-tRNA synthetase 2, mitochondrial |
| rs13213285 | 6 | 88386661 | intron |  |  | [T/C] | Fat mass change (kg/y) | 4.37E-06 | 0.036 | A | 0.034 | *ORC3* | origin recognition complex, subunit 3 |
| rs13213285 | 6 | 88386661 | intron |  |  | [T/C] | Fat mass deposition (kcal/d) | 3.73E-06 | 0.036 | A | 0.034 | *ORC3* | origin recognition complex, subunit 3 |
| rs13206957 | 6 | 88410415 | intron |  |  | [T/C] | Fat mass change (kg/y) | 7.93E-06 | 0.035 | A | 0.031 | *ORC3* | origin recognition complex, subunit 3 |
| rs2307393 | 6 | 88419662 | coding | SYNON | L498L(NP_036513.2) | [A/G] | Fat mass change (kg/y) | 5.08E-06 | 0.036 | A | 0.031 | *ORC3* | origin recognition complex, subunit 3 |
| rs2307393 | 6 | 88419662 | coding | SYNON | L498L(NP_036513.2) | [A/G] | Fat mass deposition (kcal/d) | 9.20E-06 | 0.034 | A | 0.031 | *ORC3* | origin recognition complex, subunit 3 |
| rs28381552 | 6 | 88433768 | 3UTR |  |  | [T/C] | Fat mass change (kg/y) | 4.16E-06 | 0.037 | G | 0.025 | *ORC3* | origin recognition complex, subunit 3 |
| rs28381552 | 6 | 88433768 | 3UTR |  |  | [T/C] | Fat mass deposition (kcal/d) | 6.17E-06 | 0.035 | G | 0.025 | *ORC3* | origin recognition complex, subunit 3 |
| rs6917758 | 6 | 90738187 | intron |  |  | [A/C] | BMR (kcal/d) | 2.64E-06 | 0.038 | C | 0.145 | *BACH2* | BTB and CNC homology 1, basic leucine zipper transcription factor 2 |
| rs1383808 | 6 | 99135587 | flanking_5UTR |  |  | [T/C] | Arm span (cm) | 8.73E-06 | 0.028 | G | 0.008 | *POU3F2* | POU class 3 homeobox 2 |
| rs17430337 | 6 | 103077939 | flanking_3UTR |  |  | [T/C] | NEFA (mmol/L) | 4.53E-06 | 0.024 | A | 0.015 | *GRIK2* | glutamate receptor, ionotropic, kainate 2 |
| rs17041 | 6 | 108960186 | flanking_3UTR |  |  | [A/G] | ALT (U/L) | 5.92E-06 | 0.027 | A | 0.473 | *LACE1* | lactation elevated 1 |
| rs3823043 | 6 | 127691012 | intron |  |  | [A/C] | Total T3 (ng/dL) | 9.82E-06 | 0.023 | C | 0.038 | *ECHDC1* | enoyl CoA hydratase domain containing 1 |
| rs6907728 | 6 | 132270462 | flanking_3UTR |  |  | [A/C] | CRP (ng/mL) | 3.24E-06 | 0.036 | C | 0.186 | *ENPP1* | ectonucleotide pyrophosphatase/phosphodiesterase 1 |
| rs7756650 | 6 | 132579099 | flanking_3UTR |  |  | [A/G] | Lean body mass (kg) | 9.80E-06 | 0.029 | A | 0.125 | *MOXD1* | monooxygenase, DBH-like 1 |
| rs9388971 | 6 | 132653455 | flanking_3UTR |  |  | [T/C] | Fat free mass (kg) | 3.34E-06 | 0.031 | G | 0.149 | *MOXD1* | monooxygenase, DBH-like 1 |
| rs9388971 | 6 | 132653455 | flanking_3UTR |  |  | [T/C] | Lean body mass (kg) | 3.43E-06 | 0.032 | G | 0.149 | *MOXD1* | monooxygenase, DBH-like 1 |
| rs589756 | 6 | 132684810 | intron |  |  | [T/G] | Fat free mass (kg) | 1.44E-07 | 0.037 | A | 0.129 | *MOXD1* | monooxygenase, DBH-like 1 |
| rs589756 | 6 | 132684810 | intron |  |  | [T/G] | Lean body mass (kg) | 1.17E-07 | 0.038 | A | 0.129 | *MOXD1* | monooxygenase, DBH-like 1 |
| rs589756 | 6 | 132684810 | intron |  |  | [T/G] | Sleep energy expenditure (kcal/d) | 2.28E-06 | 0.034 | A | 0.129 | *MOXD1* | monooxygenase, DBH-like 1 |
| rs589756 | 6 | 132684810 | intron |  |  | [T/G] | Total energy expenditure (kcal/d) | 1.19E-06 | 0.037 | A | 0.129 | *MOXD1* | monooxygenase, DBH-like 1 |
| rs589756 | 6 | 132684810 | intron |  |  | [T/G] | VO2max (mL/min) | 2.16E-06 | 0.043 | A | 0.129 | *MOXD1* | monooxygenase, DBH-like 1 |
| rs589756 | 6 | 132684810 | intron |  |  | [T/G] | Waist circumference (cm) | 4.49E-06 | 0.026 | A | 0.129 | *MOXD1* | monooxygenase, DBH-like 1 |
| rs589756 | 6 | 132684810 | intron |  |  | [T/G] | Weight (kg) | 8.52E-06 | 0.024 | A | 0.129 | *MOXD1* | monooxygenase, DBH-like 1 |
| rs210937 | 6 | 135582073 | flanking_3UTR |  |  | [T/C] | Ghrelin (pg/100 µL) | 7.67E-06 | 0.035 | A | 0.345 | *MYB* | v-myb myeloblastosis viral oncogene homolog (avian) |
| rs11155053 | 6 | 139701705 | flanking_3UTR |  |  | [T/C] | HRmax (bpm) | 9.00E-06 | 0.034 | A | 0.059 | *CITED2* | Cbp/p300-interacting transactivator, with Glu/Asp-rich carboxy-terminal domain, 2 |
| rs9403521 | 6 | 144035892 | intron |  |  | [A/G] | Height z-score change (SD/y) | 8.05E-06 | 0.030 | G | 0.172 | *PHACTR2* | phosphatase and actin regulator 2 |
| rs9377063 | 6 | 147976640 | flanking_3UTR |  |  | [T/C] | Urinary free dopamine: creatinine | 8.16E-06 | 0.036 | A | 0.259 | *SAMD5* | sterile alpha motif domain containing 5 |
| rs10046456 | 6 | 151166944 | intron |  |  | [T/G] | Moderate activity (%awake time) | 2.90E-06 | 0.032 | A | 0.040 | *PLEKHG1* | pleckstrin homology domain containing, family G (with RhoGef domain) member 1 |
| rs10046456 | 6 | 151166944 | intron |  |  | [T/G] | Moderate activity (min/d) | 1.87E-06 | 0.033 | A | 0.040 | *PLEKHG1* | pleckstrin homology domain containing, family G (with RhoGef domain) member 1 |
| rs10046456 | 6 | 151166944 | intron |  |  | [T/G] | Moderate&vigorous activity (min/d) | 2.91E-06 | 0.032 | A | 0.040 | *PLEKHG1* | pleckstrin homology domain containing, family G (with RhoGef domain) member 1 |
| rs60945108 | 6 | 152122525 | flanking_5UTR |  |  | [T/C] | Light activity (min/d) | 3.09E-06 | 0.029 | A | 0.009 | *ESR1* | estrogen receptor 1 |
| rs73780860 | 6 | 152124374 | flanking_5UTR |  |  | [T/C] | Light activity (min/d) | 3.09E-06 | 0.029 | G | 0.009 | *ESR1* | estrogen receptor 1 |
| rs12190007 | 6 | 169469526 | flanking_5UTR |  |  | [T/C] | Urinary free epinephrine (nmol/d) | 4.84E-06 | 0.030 | A | 0.233 | *THBS2* | thrombospondin 2 |
| rs10946292 | 6 | 170267446 | flanking_3UTR |  |  | [A/G] | Adiponectin (ng/mL) | 2.48E-06 | 0.027 | G | 0.289 | *LOC154449* | uncharacterized LOC154449 |
| rs6972204 | 7 | 2548978 | intron |  |  | [T/C] | Bike energy expenditure (kcal/min) | 4.69E-06 | 0.031 | G | 0.171 | *BRAT1* | BRCA1-associated ATM activator 1 |
| rs314590 | 7 | 4440658 | flanking_3UTR |  |  | [T/C] | Vitamin B12 (pmol/L) | 3.77E-06 | 0.020 | G | 0.271 | *SDK1* | sidekick cell adhesion molecule 1 |
| rs2306921 | 7 | 4805791 | coding | SYNON | L1040L(NP_060529.4) | [T/C] | MCP-1 (pg/mL) | 5.94E-06 | 0.035 | A | 0.060 | *RADIL* | Ras association and DIL domains |
| rs10259199 | 7 | 7815437 | intron |  |  | [A/G] | Height change (cm/y) | 1.70E-06 | 0.035 | G | 0.091 | *LOC729852* | uncharacterized LOC729852 |
| rs12702661 | 7 | 7831276 | intron |  |  | [A/G] | Fat oxidation (%NPEE) | 1.79E-06 | 0.039 | A | 0.273 | *LOC729852* | uncharacterized LOC729852 |
| rs12702661 | 7 | 7831276 | intron |  |  | [A/G] | Total energy expenditure RQ | 1.74E-06 | 0.037 | A | 0.273 | *LOC729852* | uncharacterized LOC729852 |
| rs10486201 | 7 | 7905121 | flanking_3UTR |  |  | [T/C] | Light activity (min/d) | 6.17E-06 | 0.028 | A | 0.100 | *LOC729852* | uncharacterized LOC729852 |
| rs17143586 | 7 | 8143922 | intron |  |  | [T/G] | IL-6 (pg/mL) | 1.40E-06 | 0.028 | A | 0.022 | *ICA1* | islet cell autoantigen 1, 69kDa |
| rs10251121 | 7 | 8607890 | intron |  |  | [T/C] | HOMA-IR | 8.36E-06 | 0.030 | A | 0.183 | *NXPH1* | neurexophilin 1 |
| rs10251121 | 7 | 8607890 | intron |  |  | [T/C] | Insulin (µU/mL) | 8.50E-06 | 0.030 | A | 0.183 | *NXPH1* | neurexophilin 1 |
| rs10245124 | 7 | 8608879 | intron |  |  | [A/G] | HOMA-IR | 7.82E-06 | 0.030 | G | 0.177 | *NXPH1* | neurexophilin 1 |
| rs10245124 | 7 | 8608879 | intron |  |  | [A/G] | Insulin (µU/mL) | 8.16E-06 | 0.031 | G | 0.177 | *NXPH1* | neurexophilin 1 |
| rs737714 | 7 | 9153819 | flanking_3UTR |  |  | [A/G] | Diet carbohydrate (%energy) | 8.12E-06 | 0.032 | A | 0.367 | *NXPH1* | neurexophilin 1 |
| rs1371737 | 7 | 9156066 | flanking_3UTR |  |  | [A/C] | Diet carbohydrate (%energy) | 6.80E-06 | 0.035 | C | 0.336 | *NXPH1* | neurexophilin 1 |
| rs10243151 | 7 | 9164262 | flanking_3UTR |  |  | [A/G] | Diet carbohydrate (%energy) | 9.49E-06 | 0.034 | G | 0.368 | *NXPH1* | neurexophilin 1 |
| rs12531027 | 7 | 15302461 | intron |  |  | [A/G] | HRmax (bpm) | 2.74E-07 | 0.040 | G | 0.034 | *AGMO* | alkylglycerol monooxygenase |
| rs12666612 | 7 | 18341770 | flanking_5UTR |  |  | [A/G] | Vigorous activity (min/d) | 7.48E-06 | 0.033 | G | 0.088 | *HDAC9* | histone deacetylase 9 |
| rs2853552 | 7 | 18946792 | intron |  |  | [A/G] | BMI z-score change (SD/y) | 6.73E-06 | 0.032 | A | 0.260 | *HDAC9* | histone deacetylase 9 |
| rs430 | 7 | 19073064 | flanking_3UTR |  |  | [A/G] | Urinary free epinephrine (nmol/d) | 4.18E-07 | 0.025 | A | 0.016 | *TWIST1* | twist homolog 1 (Drosophila) |
| rs17140875 | 7 | 19214803 | flanking_5UTR |  |  | [T/G] | Sleep duration (min/d) | 4.51E-06 | 0.016 | A | 0.051 | *FERD3L* | Fer3-like (Drosophila) |
| rs11974269 | 7 | 21114203 | flanking_3UTR |  |  | [T/G] | Urinary creatinine (mmol/d) | 8.38E-08 | 0.051 | C | 0.128 | *ASS1P11* | argininosuccinate synthetase 1 pseudogene 11 |
| rs11974269 | 7 | 21114203 | flanking_3UTR |  |  | [T/G] | Urinary free dopamine (nmol/d) | 4.80E-06 | 0.041 | C | 0.128 | *ASS1P11* | argininosuccinate synthetase 1 pseudogene 11 |
| rs10950840 | 7 | 21129880 | flanking_3UTR |  |  | [A/G] | Urinary creatinine (mmol/d) | 2.95E-06 | 0.038 | G | 0.213 | *ASS1P11* | argininosuccinate synthetase 1 pseudogene 11 |
| rs10950840 | 7 | 21129880 | flanking_3UTR |  |  | [A/G] | Urinary free epinephrine (nmol/d) | 7.19E-06 | 0.041 | G | 0.213 | *ASS1P11* | argininosuccinate synthetase 1 pseudogene 11 |
| rs745580 | 7 | 25974577 | flanking_5UTR |  |  | [A/G] | Dinner intake, adj EER (kcal) | 3.41E-07 | 0.039 | A | 0.438 | *NFE2L3* | nuclear factor (erythroid-derived 2)-like 3 |
| rs6958870 | 7 | 25982488 | flanking_5UTR |  |  | [T/C] | Dinner intake, adj EER (kcal) | 2.75E-06 | 0.036 | G | 0.472 | *NFE2L3* | nuclear factor (erythroid-derived 2)-like 3 |
| rs6947773 | 7 | 25989615 | flanking_5UTR |  |  | [T/C] | Dinner intake, adj EER (kcal) | 1.67E-06 | 0.038 | G | 0.470 | *NFE2L3* | nuclear factor (erythroid-derived 2)-like 3 |
| rs245914 | 7 | 29184684 | flanking_5UTR |  |  | [A/C] | Fat mass (%) | 2.58E-06 | 0.028 | A | 0.149 | *CHN2* | chimerin (chimaerin) 2 |
| rs245914 | 7 | 29184684 | flanking_5UTR |  |  | [A/C] | Weight z-score (SD) | 5.78E-06 | 0.027 | A | 0.149 | *CHN2* | chimerin (chimaerin) 2 |
| rs17158483 | 7 | 29929049 | 3UTR |  |  | [T/C] | Sleep RQ | 9.25E-06 | 0.030 | A | 0.159 | *SCRN1* | secernin 1 |
| rs6973609 | 7 | 35573758 | flanking_3UTR |  |  | [A/G] | TBF-ß1 (pg/mL) | 8.59E-06 | 0.027 | G | 0.381 | *HERPUD2* | HERPUD family member 2 |
| rs9648428 | 7 | 36178274 | intron |  |  | [A/G] | ALT/AST | 2.73E-06 | 0.028 | A | 0.092 | *EEPD1* | endonuclease/exonuclease/phosphatase family domain containing 1 |
| rs7777593 | 7 | 36512594 | flanking_3UTR |  |  | [T/G] | Weight change (kg/y) | 9.52E-07 | 0.038 | A | 0.404 | *AOAH* | acyloxyacyl hydrolase (neutrophil) |
| rs6951258 | 7 | 37724081 | flanking_5UTR |  |  | [A/G] | RANTES (pg/mL) | 3.64E-06 | 0.031 | G | 0.015 | *GPR141* | G protein-coupled receptor 141 |
| rs2024125 | 7 | 43125002 | intron |  |  | [T/C] | Diet carbohydrate (%energy) | 5.45E-06 | 0.021 | G | 0.458 | *HECW1* | HECT, C2 and WW domain containing E3 ubiquitin protein ligase 1 |
| rs10232743 | 7 | 43877407 | flanking_5UTR |  |  | [A/G] | VO2max (mL/min) | 6.78E-06 | 0.038 | A | 0.016 | *MRPS24* | mitochondrial ribosomal protein S24 |
| rs79396556 | 7 | 44193390 | flanking_3UTR |  |  | [A/G] | Energy balance (kcal/d) | 9.76E-06 | 0.030 | A | 0.004 | *GCK* | glucokinase (hexokinase 4) |
| rs75076102 | 7 | 44194604 | flanking_3UTR |  |  | [T/C] | Energy balance (kcal/d) | 9.76E-06 | 0.030 | A | 0.004 | *GCK* | glucokinase (hexokinase 4) |
| rs700750 | 7 | 46720016 | flanking_5UTR |  |  | [T/G] | IGFBP-3 (ng/mL) | 2.88E-06 | 0.027 | A | 0.487 | *EPS15P1* | epidermal growth factor receptor pathway substrate 15 pseudogene 1 |
| rs4492324 | 7 | 49604348 | flanking_5UTR |  |  | [A/G] | RQmax | 7.97E-06 | 0.039 | G | 0.235 |  |  |
| rs679830 | 7 | 54642367 | flanking_5UTR |  |  | [A/G] | IGFBP-3 (ng/mL) | 6.04E-06 | 0.026 | A | 0.116 | *RPL31P35* | ribosomal protein L31 pseudogene 35 |
| rs1829737 | 7 | 62213435 | flanking_5UTR |  |  | [T/C] | CRP (ng/mL) | 9.01E-06 | 0.029 | G | 0.126 | *LOC442318* | chromosome 9 open reading frame 140 pseudogene |
| rs6956675 | 7 | 62215205 | flanking_5UTR |  |  | [A/G] | CRP (ng/mL) | 5.53E-06 | 0.029 | A | 0.135 | *LOC442318* | chromosome 9 open reading frame 140 pseudogene |
| rs10231112 | 7 | 62238692 | flanking_5UTR |  |  | [A/G] | CRP (ng/mL) | 9.05E-06 | 0.028 | A | 0.131 | *LOC442318* | chromosome 9 open reading frame 140 pseudogene |
| rs11766624 | 7 | 69525021 | intron |  |  | [T/C] | Arm span (cm) | 4.21E-07 | 0.035 | G | 0.153 | *AUTS2* | autism susceptibility candidate 2 |
| rs12666883 | 7 | 72632412 | flanking_5UTR |  |  | [T/G] | IGFBP-3 (ng/mL) | 6.44E-06 | 0.026 | A | 0.033 | *TBL2* | transducin (beta)-like 2 |
| rs236670 | 7 | 74897267 | flanking_3UTR |  |  | [A/C] | Diet protein (%energy) | 3.27E-06 | 0.027 | C | 0.005 | *POM121C* | POM121 membrane glycoprotein C |
| rs711302 | 7 | 74993683 | intron |  |  | [A/C] | Diet protein (%energy) | 3.27E-06 | 0.027 | C | 0.005 | *PMS2L3* | postmeiotic segregation increased 2 pseudogene 3 |
| rs3864639 | 7 | 76576527 | intron |  |  | [A/G] | Urinary nitrogen (g/d) | 2.04E-07 | 0.051 | G | 0.088 | *FAM185A* | family with sequence similarity 185, member A |
| rs4729710 | 7 | 77231675 | intron |  |  | [A/G] | Diet protein (%energy) | 8.21E-06 | 0.037 | G | 0.049 | *RSBN1L* | round spermatid basic protein 1-like |
| rs6966446 | 7 | 77288294 | flanking_5UTR |  |  | [T/C] | Diet protein (%energy) | 8.68E-06 | 0.030 | G | 0.059 | *PHTF2* | putative homeodomain transcription factor 2 |
| rs17158641 | 7 | 77306373 | flanking_5UTR |  |  | [T/C] | Diet protein (%energy) | 8.68E-06 | 0.030 | A | 0.059 | *PHTF2* | putative homeodomain transcription factor 2 |
| rs12234571 | 7 | 77387842 | intron |  |  | [T/G] | Diet protein (%energy) | 6.20E-06 | 0.032 | C | 0.056 | *PHTF2* | putative homeodomain transcription factor 2 |
| rs17159512 | 7 | 77394950 | intron |  |  | [T/G] | Diet protein (%energy) | 6.26E-06 | 0.031 | C | 0.058 | *PHTF2* | putative homeodomain transcription factor 2 |
| rs12666870 | 7 | 77996408 | intron |  |  | [T/C] | Light activity (min/d) | 2.58E-06 | 0.036 | G | 0.249 | *MAGI2* | membrane associated guanylate kinase, WW & PDZ domain containing 2 |
| rs6942458 | 7 | 81620450 | intron |  |  | [T/C] | HRmax (bpm) | 5.67E-07 | 0.053 | G | 0.219 | *CACNA2D1* | calcium channel, voltage-dependent, alpha 2/delta subunit 1 |
| rs17158675 | 7 | 83620775 | intron |  |  | [A/G] | Urinary free epinephrine (nmol/d) | 8.68E-06 | 0.034 | A | 0.057 | *SEMA3A* | sema domain, immunoglobulin domain, short basic domain, secreted, (semaphorin) 3A |
| rs1476442 | 7 | 91110280 | flanking_3UTR |  |  | [A/C] | LDL (mg/dL) | 2.83E-06 | 0.023 | C | 0.221 | *MTERF* | mitochondrial transcription termination factor |
| rs11975386 | 7 | 93542969 | flanking_5UTR |  |  | [A/C] | ALT/AST | 6.66E-06 | 0.025 | A | 0.145 | *BET1* | blocked early in transport 1 homolog (S. cerevisiae) |
| rs10487245 | 7 | 93551457 | flanking_5UTR |  |  | [A/G] | ALT/AST | 1.77E-06 | 0.028 | A | 0.148 | *BET1* | blocked early in transport 1 homolog (S. cerevisiae) |
| rs11973087 | 7 | 93604384 | flanking_5UTR |  |  | [T/C] | ALT/AST | 5.09E-06 | 0.026 | A | 0.179 | *BET1* | blocked early in transport 1 homolog (S. cerevisiae) |
| rs1990543 | 7 | 93650522 | flanking_5UTR |  |  | [T/G] | ALT/AST | 4.17E-06 | 0.026 | C | 0.227 | *BET1* | blocked early in transport 1 homolog (S. cerevisiae) |
| rs1488515 | 7 | 95469132 | intron |  |  | [T/G] | Fat oxidation (%NPEE) | 1.40E-06 | 0.037 | C | 0.225 | *DYNC1I1* | dynein, cytoplasmic 1, intermediate chain 1 |
| rs1488515 | 7 | 95469132 | intron |  |  | [T/G] | Total energy expenditure RQ | 1.66E-06 | 0.035 | C | 0.225 | *DYNC1I1* | dynein, cytoplasmic 1, intermediate chain 1 |
| rs77443149 | 7 | 97762490 | intron |  |  | [A/G] | MCP-1 (pg/mL) | 8.28E-06 | 0.037 | A | 0.039 | *BAIAP2L1* | BAI1-associated protein 2-like 1 |
| rs13228694 | 7 | 99778243 | flanking_5UTR |  |  | [A/G] | IGF-1 free (ng/mL) | 5.85E-06 | 0.030 | A | 0.043 | *PMS2L1* | postmeiotic segregation increased 2 pseudogene 1 |
| rs13438712 | 7 | 105329816 | flanking_5UTR |  |  | [A/G] | Arm span (cm) | 1.90E-06 | 0.030 | G | 0.200 | *ATXN7L1* | ataxin 7-like 1 |
| rs13438712 | 7 | 105329816 | flanking_5UTR |  |  | [A/G] | Total energy expenditure (kcal/d) | 7.69E-06 | 0.030 | G | 0.200 | *ATXN7L1* | ataxin 7-like 1 |
| rs1419607 | 7 | 125178881 | flanking_5UTR |  |  | [T/C] | IL-6 (pg/mL) | 7.24E-06 | 0.031 | G | 0.372 | *LOC646841* | similar to ribosomal protein L31 |
| rs6974130 | 7 | 125195173 | flanking_5UTR |  |  | [T/C] | IL-6 (pg/mL) | 7.24E-06 | 0.031 | A | 0.372 | *LOC646841* | similar to ribosomal protein L31 |
| rs9641855 | 7 | 129143442 | intron |  |  | [A/G] | Sleep duration (min/d) | 1.48E-06 | 0.024 | G | 0.335 | *NRF1* | nuclear respiratory factor 1 |
| rs12707249 | 7 | 135130113 | flanking_5UTR |  |  | [A/G] | Glucose (mg/dL) | 2.64E-06 | 0.019 | A | 0.292 | *UNQ1940 -- FAM180A* | family with sequence similarity 180, member A |
| rs194151 | 7 | 139204416 | intron |  |  | [T/C] | Bike energy expenditure (kcal/min) | 9.29E-06 | 0.032 | A | 0.420 | *TBXAS1* | thromboxane A synthase 1 (platelet) |
| rs11976180 | 7 | 143372513 | flanking_5UTR |  |  | [A/C] | Light activity (min/d) | 7.36E-06 | 0.026 | C | 0.195 | *OR2A5* | olfactory receptor, family 2, subfamily A, member 5 |
| rs1358075 | 7 | 145766871 | intron |  |  | [A/G] | TBF-ß1 (pg/mL) | 8.66E-06 | 0.029 | G | 0.012 | *CNTNAP2* | contactin associated protein-like 2 |
| rs4726178 | 7 | 151903280 | flanking_5UTR |  |  | [A/G] | IGFBP-3 (ng/mL) | 9.72E-06 | 0.025 | A | 0.351 |  |  |
| rs11981919 | 7 | 152300773 | flanking_3UTR |  |  | [A/G] | Weight (kg) | 9.10E-06 | 0.023 | A | 0.010 | *ACTR3B* | ARP3 actin-related protein 3 homolog B (yeast) |
| rs10267337 | 7 | 155461510 | flanking_5UTR |  |  | [A/C] | ALT (U/L) | 8.00E-06 | 0.025 | A | 0.040 | *tcag7.1213* | uncharacterized LOC393076 |
| rs7786808 | 7 | 157915867 | intron |  |  | [A/G] | Diet protein (g/d) | 7.57E-06 | 0.029 | G | 0.445 | *PTPRN2* | protein tyrosine phosphatase, receptor type, N polypeptide 2 |
| rs13263558 | 8 | 1938707 | 3UTR |  |  | [T/C] | MCP-1 (pg/mL) | 3.19E-06 | 0.041 | A | 0.062 | *KBTBD11* | kelch repeat and BTB (POZ) domain containing 11 |
| rs2551043 | 8 | 3177303 | intron |  |  | [T/C] | Vigorous activity (min/d) | 7.29E-06 | 0.029 | G | 0.234 | *CSMD1* | CUB and Sushi multiple domains 1 |
| rs17394429 | 8 | 3304394 | intron |  |  | [A/G] | Total homocysteine (µmol/L) | 6.85E-06 | 0.028 | A | 0.110 | *CSMD1* | CUB and Sushi multiple domains 1 |
| rs1600857 | 8 | 3465744 | intron |  |  | [A/C] | Light activity (%awake time) | 6.60E-06 | 0.023 | A | 0.340 | *CSMD1* | CUB and Sushi multiple domains 1 |
| rs2449215 | 8 | 3475746 | intron |  |  | [A/G] | Light activity (%awake time) | 6.50E-07 | 0.035 | G | 0.380 | *CSMD1* | CUB and Sushi multiple domains 1 |
| rs9644772 | 8 | 8595095 | flanking_5UTR |  |  | [A/G] | IGFBP-3 (ng/mL) | 7.67E-06 | 0.026 | A | 0.406 | *CLDN23* | claudin 23 |
| rs9644773 | 8 | 8595490 | flanking_5UTR |  |  | [T/C] | IGFBP-3 (ng/mL) | 4.18E-06 | 0.027 | G | 0.433 | *CLDN23* | claudin 23 |
| rs12153 | 8 | 8598319 | 3UTR |  |  | [C/G] | IGFBP-3 (ng/mL) | 6.39E-06 | 0.026 | G | 0.404 | *CLDN23* | claudin 23 |
| rs1060106 | 8 | 8598862 | 3UTR |  |  | [A/G] | IGFBP-3 (ng/mL) | 3.30E-06 | 0.028 | G | 0.407 | *CLDN23* | claudin 23 |
| rs11249884 | 8 | 8598962 | 3UTR |  |  | [T/C] | IGFBP-3 (ng/mL) | 4.60E-06 | 0.027 | G | 0.405 | *CLDN23* | claudin 23 |
| rs885000 | 8 | 8606285 | flanking_3UTR |  |  | [A/C] | IGFBP-3 (ng/mL) | 3.87E-06 | 0.028 | A | 0.412 | *CLDN23* | claudin 23 |
| rs13256369 | 8 | 8614789 | flanking_3UTR |  |  | [T/C] | IGFBP-3 (ng/mL) | 3.29E-06 | 0.028 | G | 0.413 | *CLDN23* | claudin 23 |
| rs4281086 | 8 | 10389718 | flanking_5UTR |  |  | [T/G] | Waist circumference change (cm/y) | 3.75E-06 | 0.036 | C | 0.304 | *UNQ9391 -- PRSS55* | protease, serine, 55 |
| rs4841398 | 8 | 10500216 | flanking_3UTR |  |  | [A/G] | Cystathionine (µmol/L) | 2.68E-06 | 0.022 | G | 0.160 | *RP1L1* | retinitis pigmentosa 1-like 1 |
| rs6996950 | 8 | 10507227 | coding | SYNON | G597G(NP_849188.4) | [T/C] | Cystathionine (µmol/L) | 9.72E-06 | 0.016 | G | 0.163 | *RP1L1* | retinitis pigmentosa 1-like 1 |
| rs1671400 | 8 | 13220778 | intron |  |  | [A/G] | TBF-ß1 (pg/mL) | 3.66E-06 | 0.032 | A | 0.153 | *DLC1* | deleted in liver cancer 1 |
| rs11203649 | 8 | 14561665 | intron |  |  | [A/G] | Urinary free epinephrine (nmol/d) | 5.32E-07 | 0.054 | A | 0.177 | *SGCZ* | sarcoglycan, zeta |
| rs10097849 | 8 | 14574222 | intron |  |  | [T/G] | Urinary free epinephrine (nmol/d) | 5.52E-07 | 0.053 | A | 0.285 | *SGCZ* | sarcoglycan, zeta |
| rs9657208 | 8 | 14574828 | intron |  |  | [T/G] | Urinary free epinephrine (nmol/d) | 1.50E-06 | 0.049 | A | 0.287 | *SGCZ* | sarcoglycan, zeta |
| rs920616 | 8 | 14587953 | intron |  |  | [T/G] | Urinary free epinephrine (nmol/d) | 3.81E-06 | 0.048 | C | 0.267 | *SGCZ* | sarcoglycan, zeta |
| rs6989198 | 8 | 14591655 | intron |  |  | [A/C] | sICAM-1 (pg/mL) | 5.48E-06 | 0.028 | C | 0.391 | *SGCZ* | sarcoglycan, zeta |
| rs11989868 | 8 | 14594527 | intron |  |  | [T/G] | sICAM-1 (pg/mL) | 2.29E-06 | 0.030 | C | 0.386 | *SGCZ* | sarcoglycan, zeta |
| rs13282394 | 8 | 14596436 | intron |  |  | [A/C] | Urinary free epinephrine (nmol/d) | 4.30E-06 | 0.048 | A | 0.264 | *SGCZ* | sarcoglycan, zeta |
| rs13271465 | 8 | 17326782 | flanking_5UTR |  |  | [A/G] | Total glutathione (µmol/L) | 7.61E-06 | 0.027 | A | 0.244 | *ADAM24P* | ADAM metallopeptidase domain 24, pseudogene |
| rs7844500 | 8 | 18747623 | intron |  |  | [A/G] | Bone mineral density (g/cm^2^) | 9.86E-06 | 0.035 | A | 0.479 | *PSD3* | pleckstrin and Sec7 domain containing 3 |
| rs7833787 | 8 | 18752151 | intron |  |  | [T/C] | Bone mineral density (g/cm^2^) | 5.41E-06 | 0.037 | A | 0.474 | *PSD3* | pleckstrin and Sec7 domain containing 3 |
| rs11776272 | 8 | 21940490 | intron |  |  | [T/C] | Birth weight (kg) | 5.01E-06 | 0.048 | G | 0.180 | *NPM2* | nucleophosmin/nucleoplasmin 2 |
| rs76547188 | 8 | 22034317 | coding |  |  | [A/G] | TBF-ß1 (pg/mL) | 4.60E-06 | 0.031 | A | 0.005 | *HR* | hairless homolog (mouse) |
| rs13280242 | 8 | 28812657 | intron |  |  | [A/G] | Free T3 (pg/mL) | 6.93E-06 | 0.024 | G | 0.224 | *HMBOX1* | homeobox containing 1 |
| rs2221894 | 8 | 28847078 | intron |  |  | [A/G] | Free T3 (pg/mL) | 6.20E-06 | 0.024 | G | 0.228 | *HMBOX1* | homeobox containing 1 |
| rs2575029 | 8 | 31294372 | flanking_5UTR |  |  | [T/C] | Diet protein (%energy) | 6.23E-06 | 0.026 | A | 0.229 | *LOC642513* | potassium channel tetramerisation domain containing 9 pseudogene |
| rs7814403 | 8 | 36578612 | flanking_5UTR |  |  | [A/G] | Diet carbohydrate (g/d) | 3.21E-07 | 0.033 | G | 0.462 | *FKSG2* | tumor protein, translationally-controlled 1 pseudogene |
| rs7814403 | 8 | 36578612 | flanking_5UTR |  |  | [A/G] | Energy intake (kcal/d) | 2.37E-06 | 0.028 | G | 0.462 | *FKSG2* | tumor protein, translationally-controlled 1 pseudogene |
| rs7822058 | 8 | 53847414 | flanking_3UTR |  |  | [T/C] | Leptin (ng/mL) | 2.97E-07 | 0.036 | A | 0.076 | *LOC644727* | uncharacterized LOC644727 |
| rs7827790 | 8 | 56937095 | flanking_5UTR |  |  | [T/C] | Fat mass (%) | 9.90E-06 | 0.031 | A | 0.075 | *LYN* | v-yes-1 Yamaguchi sarcoma viral related oncogene homolog |
| rs12545109 | 8 | 57476460 | flanking_5UTR |  |  | [A/C] | Light activity (min/d) | 9.12E-06 | 0.035 | A | 0.225 | *SDR16C6P* | short chain dehydrogenase/reductase family 16C, member 6, pseudogene |
| rs12541902 | 8 | 60785902 | flanking_3UTR |  |  | [A/C] | Urinary free dopamine: creatinine | 8.96E-06 | 0.027 | A | 0.154 | *CA8* | carbonic anhydrase VIII |
| rs4237035 | 8 | 60788653 | flanking_3UTR |  |  | [A/G] | Urinary free dopamine: creatinine | 9.66E-06 | 0.026 | A | 0.131 | *CA8* | carbonic anhydrase VIII |
| rs720208 | 8 | 61258730 | flanking_3UTR |  |  | [A/G] | Urinary free dopamine (nmol/d) | 6.54E-06 | 0.033 | A | 0.286 | *CA8* | carbonic anhydrase VIII |
| rs10098647 | 8 | 61267753 | intron |  |  | [T/C] | Urinary free dopamine (nmol/d) | 4.95E-06 | 0.033 | G | 0.300 | *CA8* | carbonic anhydrase VIII |
| rs6981992 | 8 | 66626032 | flanking_3UTR |  |  | [T/G] | Sleep duration (min/d) | 7.69E-06 | 0.024 | A | 0.489 | *ARMC1* | armadillo repeat containing 1 |
| rs920016 | 8 | 69703772 | intron |  |  | [A/C] | AST (U/L) | 3.26E-07 | 0.038 | C | 0.238 | *C8orf34* | chromosome 8 open reading frame 34 |
| rs7825271 | 8 | 69726249 | intron |  |  | [T/G] | AST (U/L) | 2.80E-07 | 0.038 | C | 0.251 | *C8orf34* | chromosome 8 open reading frame 34 |
| rs16935110 | 8 | 69893273 | 3UTR |  |  | [A/T] | Urinary free norepinephrine (nmol/d) | 5.35E-06 | 0.033 | T | 0.116 | *C8orf34* | chromosome 8 open reading frame 34 |
| rs1909881 | 8 | 96585064 | flanking_5UTR |  |  | [A/G] | IL-6 (pg/mL) | 7.09E-06 | 0.025 | G | 0.089 | *C8orf37* | chromosome 8 open reading frame 37 |
| rs2640806 | 8 | 97438810 | flanking_3UTR |  |  | [T/G] | Methionine (µmol/L) | 4.65E-06 | 0.031 | A | 0.317 | *PTDSS1* | phosphatidylserine synthase 1p |
| rs10107366 | 8 | 99182863 | flanking_3UTR |  |  | [T/C] | Energy balance (kcal/d) | 6.15E-07 | 0.041 | G | 0.008 | *HRSP12* | heat-responsive protein 12 |
| rs7004587 | 8 | 108109528 | flanking_3UTR |  |  | [T/C] | HDL (mg/dL) | 2.88E-06 | 0.023 | A | 0.007 |  |  |
| rs73313124 | 8 | 110547998 | coding |  |  | [T/G] | Urinary free epinephrine (nmol/d) | 2.81E-06 | 0.021 | A | 0.003 | *PKHD1L1* | polycystic kidney and hepatic disease 1 (autosomal recessive)-like 1 |
| rs11989843 | 8 | 118220174 | intron |  |  | [A/G] | ALT/AST | 8.33E-06 | 0.025 | G | 0.167 | *SLC30A8* | solute carrier family 30 (zinc transporter), member 8 |
| rs7459527 | 8 | 118978441 | intron |  |  | [T/G] | Cortisol (ng/mL) | 1.68E-06 | 0.025 | A | 0.093 | *EXT1* | exostosin 1 |
| rs6999867 | 8 | 118999922 | intron |  |  | [T/C] | Cortisol (ng/mL) | 2.55E-06 | 0.023 | A | 0.105 | *EXT1* | exostosin 1 |
| rs11995252 | 8 | 122691090 | flanking_3UTR |  |  | [T/C] | Folate (nmol/L) | 4.57E-06 | 0.025 | G | 0.047 | *HAS2* | hyaluronan synthase 2 |
| rs921720 | 8 | 126603853 | flanking_3UTR |  |  | [A/G] | Gestational age (wk) | 2.68E-06 | 0.050 | A | 0.414 | *TRIB1* | tribbles homolog 1 (Drosophila) |
| rs1551398 | 8 | 126609233 | flanking_3UTR |  |  | [A/G] | Gestational age (wk) | 2.68E-06 | 0.050 | G | 0.414 | *TRIB1* | tribbles homolog 1 (Drosophila) |
| rs4871750 | 8 | 127971185 | flanking_5UTR |  |  | [A/G] | Bone mineral content (kg) | 4.12E-07 | 0.035 | A | 0.383 | *FAM84B* | family with sequence similarity 84, member B |
| rs4871750 | 8 | 127971185 | flanking_5UTR |  |  | [A/G] | Bone mineral density (g/cm^2^) | 9.11E-06 | 0.027 | A | 0.383 | *FAM84B* | family with sequence similarity 84, member B |
| rs7817717 | 8 | 127972444 | flanking_5UTR |  |  | [T/G] | Bone mineral content (kg) | 6.88E-07 | 0.033 | C | 0.385 | *FAM84B* | family with sequence similarity 84, member B |
| rs2220321 | 8 | 127980778 | flanking_5UTR |  |  | [T/C] | Bone mineral content (kg) | 5.45E-07 | 0.034 | A | 0.385 | *FAM84B* | family with sequence similarity 84, member B |
| rs2220321 | 8 | 127980778 | flanking_5UTR |  |  | [T/C] | Bone mineral density (g/cm^2^) | 7.94E-06 | 0.027 | A | 0.385 | *FAM84B* | family with sequence similarity 84, member B |
| rs7012789 | 8 | 127986390 | flanking_5UTR |  |  | [T/C] | Bone mineral content (kg) | 6.11E-07 | 0.034 | G | 0.386 | *FAM84B* | family with sequence similarity 84, member B |
| rs7012789 | 8 | 127986390 | flanking_5UTR |  |  | [T/C] | Bone mineral density (g/cm^2^) | 9.44E-06 | 0.027 | G | 0.386 | *FAM84B* | family with sequence similarity 84, member B |
| rs4871752 | 8 | 127989768 | flanking_5UTR |  |  | [A/G] | Bone mineral content (kg) | 4.19E-07 | 0.035 | A | 0.383 | *FAM84B* | family with sequence similarity 84, member B |
| rs4871752 | 8 | 127989768 | flanking_5UTR |  |  | [A/G] | Bone mineral density (g/cm^2^) | 9.65E-06 | 0.027 | A | 0.383 | *FAM84B* | family with sequence similarity 84, member B |
| rs11995854 | 8 | 129289255 | flanking_5UTR |  |  | [A/C] | Diet carbohydrate (g/d) | 7.54E-06 | 0.035 | C | 0.277 | *TMEM75* | transmembrane protein 75 |
| rs984440 | 8 | 139146910 | intron |  |  | [A/G] | RANTES (pg/mL) | 2.93E-06 | 0.026 | A | 0.127 | *FLJ45872* | FLJ45872 protein |
| rs1870805 | 8 | 141064425 | flanking_3UTR |  |  | [T/C] | Urinary nitrogen (g/d) | 2.93E-06 | 0.033 | A | 0.275 | *TRAPPC9* | trafficking protein particle complex 9 |
| rs3816063 | 8 | 142230246 | intron |  |  | [T/C] | Diet protein (%energy) | 1.95E-06 | 0.027 | A | 0.242 | *DENND3* | DENN/MADD domain containing 3 |
| rs1566080 | 8 | 142625987 | flanking_5UTR |  |  | [T/G] | Total antioxidants (mM) | 5.89E-06 | 0.026 | A | 0.220 | *FLJ43860* | FLJ43860 protein |
| rs4072286 | 8 | 142805713 | flanking_3UTR |  |  | [A/C] | Eotaxin (pg/mL) | 4.18E-07 | 0.033 | C | 0.461 |  |  |
| rs35925379 | 8 | 144874444 | coding | NONSYN | R277W(NP_620590.2) | [A/G] | IGFBP-3 (ng/mL) | 1.81E-06 | 0.029 | A | 0.052 | *MAPK15* | mitogen-activated protein kinase 15 |
| rs6476030 | 9 | 279061 | intron |  |  | [T/C] | Hip circumference change (cm/y) | 3.27E-06 | 0.022 | G | 0.086 | *DOCK8* | dedicator of cytokinesis 8 |
| rs2297075 | 9 | 380512 | coding | SYNON | T904T(NP_982272.1) | [A/G] | Hip circumference change (cm/y) | 7.56E-06 | 0.029 | A | 0.208 | *DOCK8* | dedicator of cytokinesis 8 |
| rs912062 | 9 | 831152 | flanking_5UTR |  |  | [T/G] | Diet carbohydrate (g/d) | 2.55E-06 | 0.028 | C | 0.161 | *DMRT1* | doublesex and mab-3 related transcription factor 1 |
| rs1407808 | 9 | 837292 | intron |  |  | [T/G] | Diet carbohydrate (g/d) | 4.15E-06 | 0.025 | C | 0.153 | *DMRT1* | doublesex and mab-3 related transcription factor 1 |
| rs930811 | 9 | 2686555 | flanking_5UTR |  |  | [A/G] | Birth weight (kg) | 1.21E-06 | 0.042 | G | 0.406 | *KCNV2* | potassium channel, subfamily V, member |
| rs10757686 | 9 | 2777856 | flanking_3UTR |  |  | [T/G] | Birth weight (kg) | 9.30E-06 | 0.034 | C | 0.488 | *KIAA0020* | KIAA0020 |
| rs10968457 | 9 | 2828470 | 5UTR |  |  | [A/G] | Diastolic blood pressure (mmHg) | 9.44E-06 | 0.025 | A | 0.047 | *KIAA0020* | KIAA0020 |
| rs1535454 | 9 | 5400723 | intron |  |  | [T/C] | Triglycerides (mg/dL) | 3.42E-06 | 0.029 | A | 0.045 | *C9orf46* | chromosome 9 open reading frame 46 |
| rs2820914 | 9 | 7185408 | flanking_3UTR |  |  | [A/G] | Triglycerides (mg/dL) | 2.41E-06 | 0.024 | G | 0.140 | *JMJD2C* | lysine (K)-specific demethylase 4C |
| rs10815578 | 9 | 7263950 | flanking_3UTR |  |  | [A/G] | Total energy expenditure RQ | 5.69E-06 | 0.032 | A | 0.301 | *JMJD2C* | lysine (K)-specific demethylase 4C |
| rs1887867 | 9 | 7264696 | flanking_3UTR |  |  | [T/C] | Fat oxidation (%NPEE) | 5.45E-06 | 0.034 | G | 0.405 | *JMJD2C* | lysine (K)-specific demethylase 4C |
| rs1887867 | 9 | 7264696 | flanking_3UTR |  |  | [T/C] | Total energy expenditure RQ | 1.34E-06 | 0.037 | G | 0.405 | *JMJD2C* | lysine (K)-specific demethylase 4C |
| rs7034160 | 9 | 7266798 | flanking_3UTR |  |  | [A/G] | Fat oxidation (%NPEE) | 7.92E-06 | 0.034 | G | 0.380 | *JMJD2C* | lysine (K)-specific demethylase 4C |
| rs10815582 | 9 | 7274763 | flanking_3UTR |  |  | [T/C] | Fat oxidation (%NPEE) | 8.01E-06 | 0.034 | G | 0.382 | *JMJD2C* | lysine (K)-specific demethylase 4C |
| rs4742322 | 9 | 7276599 | flanking_3UTR |  |  | [A/C] | Total energy expenditure RQ | 4.67E-06 | 0.033 | A | 0.361 | *JMJD2C* | lysine (K)-specific demethylase 4C |
| rs4400445 | 9 | 7548261 | flanking_5UTR |  |  | [A/G] | Hip circumference (cm) | 8.19E-06 | 0.026 | A | 0.124 | *LOC392285* | peptidyl-prolyl cis-trans isomerase A-like |
| rs4400445 | 9 | 7548261 | flanking_5UTR |  |  | [A/G] | Weight z-score (SD) | 8.75E-06 | 0.024 | A | 0.124 | *LOC392285* | peptidyl-prolyl cis-trans isomerase A-like |
| rs294845 | 9 | 10129580 | flanking_3UTR |  |  | [T/C] | Dinner intake, adj TEE (kcal) | 7.39E-06 | 0.036 | A | 0.239 | *PTPRD* | protein tyrosine phosphatase, receptor type, D |
| rs1471871 | 9 | 11724178 | flanking_5UTR |  |  | [T/C] | Vigorous activity (min/d) | 9.70E-06 | 0.026 | A | 0.414 | *LOC646087* | IMP3, U3 small nucleolar ribonucleoprotein, homolog (yeast) pseudogene |
| rs16933006 | 9 | 15325914 | flanking_5UTR |  |  | [A/C] | Light activity (%awake time) | 9.75E-07 | 0.030 | C | 0.110 | *RPL7P3* | ribosomal protein L7 pseudogene 33 |
| rs16933006 | 9 | 15325914 | flanking_5UTR |  |  | [A/C] | Light activity (min/d) | 7.49E-08 | 0.035 | C | 0.110 | *RPL7P3* | ribosomal protein L7 pseudogene 33 |
| rs16933006 | 9 | 15325914 | flanking_5UTR |  |  | [A/C] | Sedentary activity (%awake time) | 4.12E-06 | 0.028 | C | 0.110 | *RPL7P3* | ribosomal protein L7 pseudogene 33 |
| rs2026766 | 9 | 17946000 | flanking_3UTR |  |  | [A/C] | Light activity (min/d) | 3.92E-06 | 0.039 | C | 0.207 | *SH3GL2* | SH3-domain GRB2-like 2 |
| rs1620986 | 9 | 17951642 | flanking_3UTR |  |  | [T/G] | Light activity (min/d) | 2.70E-06 | 0.039 | C | 0.284 | *SH3GL2* | SH3-domain GRB2-like 2 |
| rs2772686 | 9 | 17953004 | flanking_3UTR |  |  | [T/C] | Light activity (min/d) | 2.76E-06 | 0.039 | G | 0.279 | *SH3GL2* | SH3-domain GRB2-like 2 |
| rs1755271 | 9 | 17969578 | flanking_3UTR |  |  | [A/G] | Light activity (min/d) | 2.05E-06 | 0.038 | A | 0.298 | *SH3GL2* | SH3-domain GRB2-like 2 |
| rs6475216 | 9 | 18434138 | flanking_5UTR |  |  | [T/C] | Trunk fat mass (kg) | 8.68E-06 | 0.030 | A | 0.484 | *ADAMTSL1* | ADAMTS-like 1 |
| rs1340043 | 9 | 18448068 | flanking_5UTR |  |  | [A/G] | Fat mass (kg) | 9.16E-06 | 0.030 | A | 0.410 | *ADAMTSL1* | ADAMTS-like 1 |
| rs1340043 | 9 | 18448068 | flanking_5UTR |  |  | [A/G] | Trunk fat mass (kg) | 9.27E-06 | 0.030 | A | 0.410 | *ADAMTSL1* | ADAMTS-like 1 |
| rs10967875 | 9 | 27299659 | flanking_5UTR |  |  | [T/C] | Systolic blood pressure (mmHg) | 5.84E-06 | 0.028 | G | 0.275 | *C9orf11* | chromosome 9 open reading frame 11 |
| rs16912725 | 9 | 28349782 | intron |  |  | [T/G] | IGF-1 free (ng/mL) | 2.11E-06 | 0.037 | A | 0.004 | *LINGO2* | leucine rich repeat and Ig domain containing 2 |
| rs16912739 | 9 | 28354240 | intron |  |  | [A/G] | IGF-1 free (ng/mL) | 2.11E-06 | 0.037 | A | 0.004 | *LINGO2* | leucine rich repeat and Ig domain containing 2 |
| rs10968562 | 9 | 28392043 | intron |  |  | [T/C] | BMI z-score change (SD/y) | 6.97E-06 | 0.030 | G | 0.302 | *LINGO2* | leucine rich repeat and Ig domain containing 2 |
| rs13290799 | 9 | 29535875 | flanking_5UTR |  |  | [A/G] | Diet fat (%energy) | 3.65E-06 | 0.028 | A | 0.129 | *LINGO2* | leucine rich repeat and Ig domain containing 2 |
| rs10969375 | 9 | 29773989 | flanking_5UTR |  |  | [T/C] | HDL (mg/dL) | 5.74E-06 | 0.021 | G | 0.140 | *LINGO2* | leucine rich repeat and Ig domain containing 2 |
| rs10813168 | 9 | 29778364 | flanking_3UTR |  |  | [T/G] | HDL (mg/dL) | 7.38E-06 | 0.021 | A | 0.137 | *LOC401497* | uncharacterized LOC401497 |
| rs7039377 | 9 | 38665465 | flanking_3UTR |  |  | [A/G] | Total glutathione (µmol/L) | 1.67E-06 | 0.029 | A | 0.496 | *LOC647051* | tyrosine 3-monooxygenase/tryptophan 5-monooxygenase activation protein, beta polypeptide pseudogene |
| rs17058153 | 9 | 74596979 | coding | NONSYN | M486T(NP_619636.2) | [T/C] | C-peptide (ng/mL) | 9.73E-06 | 0.035 | G | 0.009 | *TMC1* | transmembrane channel-like 1 |
| rs12555078 | 9 | 75347520 | flanking_5UTR |  |  | [A/G] | RANTES (pg/mL) | 2.18E-06 | 0.034 | A | 0.098 | *LOC138972* | similar to centaurin, gamma 2 isoform 2 |
| rs10125737 | 9 | 80273717 | flanking_3UTR |  |  | [A/G] | Vigorous activity (%awake time) | 5.68E-06 | 0.028 | A | 0.294 | *PSAT1* | phosphoserine aminotransferase 1 |
| rs10867921 | 9 | 84799601 | intron |  |  | [T/C] | Bike energy expenditure (kcal/min) | 7.72E-06 | 0.030 | A | 0.030 | *RASEF* | RAS and EF-hand domain containing |
| rs12000457 | 9 | 86134245 | intron |  |  | [T/C] | Sleep duration (min/d) | 4.03E-06 | 0.026 | A | 0.040 | *SLC28A3* | solute carrier family 28 (sodium-coupled nucleoside transporter), member 3 |
| rs17054265 | 9 | 90450382 | flanking_3UTR |  |  | [A/G] | Gestational age (wk) | 3.85E-06 | 0.047 | A | 0.170 | *LOC286238* | uncharacterized LOC286238 |
| rs10125337 | 9 | 96483859 | flanking_5UTR |  |  | [A/G] | CRP (ng/mL) | 3.92E-06 | 0.031 | G | 0.004 | *FBP1* | fructose-1,6-bisphosphatase 1 |
| rs10120534 | 9 | 96503622 | flanking_5UTR |  |  | [T/G] | CRP (ng/mL) | 3.92E-06 | 0.031 | C | 0.004 | *C9orf3* | chromosome 9 open reading frame 3 |
| rs1475545 | 9 | 99356051 | intron |  |  | [T/C] | Urinary free dopamine (nmol/d) | 5.19E-06 | 0.033 | A | 0.394 | *TMOD1* | tropomodulin 1 |
| rs7875482 | 9 | 99536486 | flanking_3UTR |  |  | [T/C] | TSH (µIU/mL) | 9.84E-06 | 0.024 | A | 0.301 | *XPA / FOXE1* | xeroderma pigmentosum, complementation group A / forkhead box E1 (thyroid transcription factor 2) |
| rs2805771 | 9 | 99543836 | flanking_3UTR |  |  | [A/G] | TSH (µIU/mL) | 3.43E-06 | 0.026 | A | 0.283 | *XPA / FOXE1* | xeroderma pigmentosum, complementation group A / forkhead box E1 (thyroid transcription factor 2) |
| rs2808699 | 9 | 99545651 | flanking_3UTR |  |  | [A/C] | TSH (µIU/mL) | 6.02E-06 | 0.024 | A | 0.294 | *XPA / FOXE1* | xeroderma pigmentosum, complementation group A / forkhead box E1 (thyroid transcription factor 2) |
| rs10818037 | 9 | 99565123 | flanking_5UTR |  |  | [C/G] | TSH (µIU/mL) | 1.68E-06 | 0.023 | G | 0.299 | *XPA / FOXE1* | xeroderma pigmentosum, complementation group A / forkhead box E1 (thyroid transcription factor 2) |
| rs1912995 | 9 | 99570720 | flanking_5UTR |  |  | [A/G] | TSH (µIU/mL) | 1.00E-06 | 0.024 | A | 0.296 | *XPA / FOXE1* | xeroderma pigmentosum, complementation group A / forkhead box E1 (thyroid transcription factor 2) |
| rs1877431 | 9 | 99573968 | flanking_3UTR |  |  | [A/G] | TSH (µIU/mL) | 5.50E-08 | 0.030 | A | 0.287 | *XPA / FOXE1* | xeroderma pigmentosum, complementation group A / forkhead box E1 (thyroid transcription factor 2) |
| rs7045465 | 9 | 99574644 | flanking_5UTR |  |  | [T/A] | TSH (µIU/mL) | 5.50E-08 | 0.030 | A | 0.287 | *XPA / FOXE1* | xeroderma pigmentosum, complementation group A / forkhead box E1 (thyroid transcription factor 2) |
| rs7030256 | 9 | 99575024 | flanking_5UTR |  |  | [C/G] | TSH (µIU/mL) | 2.51E-09 | 0.034 | C | 0.259 | *XPA / FOXE1* | xeroderma pigmentosum, complementation group A / forkhead box E1 (thyroid transcription factor 2) |
| rs7030280 | 9 | 99575088 | flanking_5UTR |  |  | [A/G] | TSH (µIU/mL) | 4.60E-09 | 0.033 | G | 0.258 | *XPA / FOXE1* | xeroderma pigmentosum, complementation group A / forkhead box E1 (thyroid transcription factor 2) |
| rs10983700 | 9 | 99577276 | flanking_5UTR |  |  | [T/C] | TSH (µIU/mL) | 2.51E-09 | 0.034 | A | 0.259 | *XPA / FOXE1* | xeroderma pigmentosum, complementation group A / forkhead box E1 (thyroid transcription factor 2) |
| rs10983701 | 9 | 99577398 | flanking_5UTR |  |  | [A/G] | TSH (µIU/mL) | 3.79E-09 | 0.034 | G | 0.260 | *XPA / FOXE1* | xeroderma pigmentosum, complementation group A / forkhead box E1 (thyroid transcription factor 2) |
| rs1588635 | 9 | 99577623 | flanking_5UTR |  |  | [T/G] | TSH (µIU/mL) | 2.51E-09 | 0.034 | A | 0.259 | *XPA / FOXE1* | xeroderma pigmentosum, complementation group A / forkhead box E1 (thyroid transcription factor 2) |
| rs7028661 | 9 | 99578291 | flanking_5UTR |  |  | [A/G] | TSH (µIU/mL) | 3.59E-09 | 0.033 | A | 0.258 | *XPA / FOXE1* | xeroderma pigmentosum, complementation group A / forkhead box E1 (thyroid transcription factor 2) |
| rs7021576 | 9 | 99580362 | flanking_5UTR |  |  | [A/G] | TSH (µIU/mL) | 4.22E-09 | 0.033 | G | 0.258 | *XPA / FOXE1* | xeroderma pigmentosum, complementation group A / forkhead box E1 (thyroid transcription factor 2) |
| rs4743130 | 9 | 99585861 | flanking_5UTR |  |  | [A/G] | TSH (µIU/mL) | 2.96E-09 | 0.034 | G | 0.259 | *XPA / FOXE1* | xeroderma pigmentosum, complementation group A / forkhead box E1 (thyroid transcription factor 2) |
| rs1561962 | 9 | 99586040 | flanking_5UTR |  |  | [A/G] | TSH (µIU/mL) | 4.15E-09 | 0.033 | G | 0.258 | *XPA / FOXE1* | xeroderma pigmentosum, complementation group A / forkhead box E1 (thyroid transcription factor 2) |
| rs925488 | 9 | 99586212 | flanking_5UTR |  |  | [A/G] | TSH (µIU/mL) | 2.74E-09 | 0.034 | G | 0.260 | *XPA / FOXE1* | xeroderma pigmentosum, complementation group A / forkhead box E1 (thyroid transcription factor 2) |
| rs925489 | 9 | 99586421 | flanking_5UTR |  |  | [T/C] | TSH (µIU/mL) | 2.74E-09 | 0.034 | G | 0.260 | *XPA / FOXE1* | xeroderma pigmentosum, complementation group A / forkhead box E1 (thyroid transcription factor 2) |
| rs7864322 | 9 | 99588755 | flanking_5UTR |  |  | [A/G] | TSH (µIU/mL) | 3.23E-09 | 0.033 | G | 0.259 | *XPA / FOXE1* | xeroderma pigmentosum, complementation group A / forkhead box E1 (thyroid transcription factor 2) |
| rs7850258 | 9 | 99588834 | flanking_5UTR |  |  | [A/G] | TSH (µIU/mL) | 2.96E-09 | 0.034 | A | 0.259 | *XPA / FOXE1* | xeroderma pigmentosum, complementation group A / forkhead box E1 (thyroid transcription factor 2) |
| rs1443438 | 9 | 99589849 | flanking_5UTR |  |  | [T/C] | TSH (µIU/mL) | 1.03E-09 | 0.038 | A | 0.253 | *XPA / FOXE1* | xeroderma pigmentosum, complementation group A / forkhead box E1 (thyroid transcription factor 2) |
| rs7030241 | 9 | 99590196 | flanking_5UTR |  |  | [T/A] | TSH (µIU/mL) | 1.03E-09 | 0.038 | T | 0.253 | *XPA / FOXE1* | xeroderma pigmentosum, complementation group A / forkhead box E1 (thyroid transcription factor 2) |
| rs7027030 | 9 | 99590276 | flanking_5UTR |  |  | [T/G] | TSH (µIU/mL) | 1.49E-09 | 0.037 | A | 0.252 | *XPA / FOXE1* | xeroderma pigmentosum, complementation group A / forkhead box E1 (thyroid transcription factor 2) |
| rs10739496 | 9 | 99592380 | flanking_5UTR |  |  | [T/C] | TSH (µIU/mL) | 2.03E-08 | 0.029 | G | 0.258 | *XPA / FOXE1* | xeroderma pigmentosum, complementation group A / forkhead box E1 (thyroid transcription factor 2) |
| rs10983761 | 9 | 99593778 | flanking_5UTR |  |  | [T/G] | TSH (µIU/mL) | 2.03E-08 | 0.029 | A | 0.258 | *XPA / FOXE1* | xeroderma pigmentosum, complementation group A / forkhead box E1 (thyroid transcription factor 2) |
| rs965513 | 9 | 99595930 | flanking_5UTR |  |  | [A/G] | TSH (µIU/mL) | 7.52E-09 | 0.033 | A | 0.252 | *XPA / FOXE1* | xeroderma pigmentosum, complementation group A / forkhead box E1 (thyroid transcription factor 2) |
| rs10759944 | 9 | 99596793 | flanking_5UTR |  |  | [T/C] | TSH (µIU/mL) | 2.03E-08 | 0.029 | A | 0.258 | *XPA / FOXE1* | xeroderma pigmentosum, complementation group A / forkhead box E1 (thyroid transcription factor 2) |
| rs10818090 | 9 | 99601307 | flanking_5UTR |  |  | [T/C] | TSH (µIU/mL) | 2.03E-08 | 0.029 | G | 0.258 | *XPA / FOXE1* | xeroderma pigmentosum, complementation group A / forkhead box E1 (thyroid transcription factor 2) |
| rs6478413 | 9 | 99621845 | flanking_3UTR |  |  | [A/G] | TSH (µIU/mL) | 6.15E-06 | 0.016 | A | 0.309 | *XPA / FOXE1* | xeroderma pigmentosum, complementation group A / forkhead box E1 (thyroid transcription factor 2) |
| rs10124220 | 9 | 99622895 | flanking_3UTR |  |  | [A/G] | TSH (µIU/mL) | 3.75E-06 | 0.017 | A | 0.307 | *XPA / FOXE1* | xeroderma pigmentosum, complementation group A / forkhead box E1 (thyroid transcription factor 2) |
| rs1443432 | 9 | 99623016 | flanking_3UTR |  |  | [T/C] | TSH (µIU/mL) | 5.11E-06 | 0.016 | G | 0.309 | *XPA / FOXE1* | xeroderma pigmentosum, complementation group A / forkhead box E1 (thyroid transcription factor 2) |
| rs7848973 | 9 | 99628660 | flanking_5UTR |  |  | [A/G] | TSH (µIU/mL) | 3.75E-06 | 0.017 | A | 0.307 | *XPA / FOXE1* | xeroderma pigmentosum, complementation group A / forkhead box E1 (thyroid transcription factor 2) |
| rs4523321 | 9 | 103577431 | flanking_5UTR |  |  | [A/G] | Vigorous activity (%awake time) | 2.08E-06 | 0.031 | A | 0.004 | *GRIN3A* | glutamate receptor, ionotropic, N-methyl-D-aspartate 3A |
| rs4523321 | 9 | 103577431 | flanking_5UTR |  |  | [A/G] | Vigorous activity (min/d) | 1.29E-06 | 0.033 | A | 0.004 | *GRIN3A* | glutamate receptor, ionotropic, N-methyl-D-aspartate 3A |
| rs2210533 | 9 | 104481938 | flanking_5UTR |  |  | [T/C] | Weight change (kg/y) | 6.45E-06 | 0.029 | A | 0.110 | *CYLC2* | cylicin, basic protein of sperm head cytoskeleton 2 |
| rs2472476 | 9 | 106571777 | intron |  |  | [T/C] | Glucose (mg/dL) | 2.73E-06 | 0.026 | G | 0.267 | *NIPSNAP3B* | nipsnap homolog 3B (C. elegans) |
| rs2025875 | 9 | 111636732 | intron |  |  | [T/C] | Total cholesterol (mg/dL) | 7.98E-06 | 0.029 | G | 0.157 | *PALM2AKAP2* | PALM2-AKAP2 readthrough |
| rs10125054 | 9 | 113837858 | flanking_3UTR |  |  | [A/G] | Sedentary activity (min/d) | 4.05E-06 | 0.027 | A | 0.302 | *SUSD1* | sushi domain containing 1 |
| rs79509430 | 9 | 116280727 | coding |  |  | [T/C] | MCP-1 (pg/mL) | 2.03E-08 | 0.056 | A | 0.068 | *DFNB31* | deafness, autosomal recessive 31 |
| rs16934284 | 9 | 118879149 | intron |  |  | [T/C] | AST (U/L) | 2.35E-06 | 0.028 | G | 0.085 | *ASTN2* | astrotactin 2 |
| rs2780246 | 9 | 119809665 | flanking_3UTR |  |  | [T/C] | Vigorous activity (min/d) | 9.11E-06 | 0.030 | A | 0.255 | *TLR4* | toll-like receptor 4 |
| rs2416604 | 9 | 119835811 | flanking_3UTR |  |  | [T/C] | Vigorous activity (min/d) | 2.57E-06 | 0.032 | A | 0.430 | *TLR4* | toll-like receptor 4 |
| rs7042758 | 9 | 123368015 | flanking_5UTR |  |  | [T/C] | LDL (mg/dL) | 9.57E-06 | 0.030 | G | 0.021 | *DAB2IP* | DAB2 interacting protein |
| rs3818638 | 9 | 123946544 | intron |  |  | [T/C] | AST (U/L) | 6.25E-06 | 0.033 | A | 0.326 | *NDUFA8* | NADH dehydrogenase (ubiquinone) 1 alpha subcomplex, 8, 19kDa |
| rs16912238 | 9 | 124612851 | flanking_3UTR |  |  | [T/C] | Weight (kg) | 4.91E-06 | 0.026 | G | 0.005 | *PDCL* | phosducin-like |
| rs758970 | 9 | 128234594 | intron |  |  | [A/G] | Total T4 (µg/dL) | 6.23E-07 | 0.047 | A | 0.309 | *FAM125B* | family with sequence similarity 125, member B |
| rs11539570 | 9 | 130124449 | coding | SYNON | G20G(NP_056494.1) | [T/C] | Waist circumference change (cm/y) | 6.81E-06 | 0.024 | A | 0.007 | *TRUB2, COQ4* | TruB pseudouridine (psi) synthase homolog 2 (E. coli); coenzyme Q4 homolog (S. cerevisiae) |
| rs72758841 | 9 | 130515404 | coding |  |  | [G/C] | IGFBP-3 (ng/mL) | 6.06E-06 | 0.026 | G | 0.036 | *PKN3* | protein kinase N3 |
| rs569434 | 9 | 134334228 | intron |  |  | [T/C] | Fat oxidation (%NPEE) | 8.59E-06 | 0.032 | G | 0.248 | *C9orf171* | chromosome 9 open reading frame 171 |
| rs10901212 | 9 | 134636842 | intron |  |  | [T/G] | IL-6 (pg/mL) | 4.61E-06 | 0.033 | C | 0.089 | *AK8* | adenylate kinase 8 |
| rs10901253 | 9 | 135118593 | flanking_3UTR |  |  | [A/G] | IL-6 (pg/mL) | 9.12E-06 | 0.028 | G | 0.452 | *ABO* | ABO blood group (transferase A, alpha 1-3-N-acetylgalactosaminyltransferase; transferase B, alpha 1-3-galactosyltransferase) |
| rs77641731 | 9 | 135122729 | coding |  |  | [A/G] | IL-6 (pg/mL) | 2.16E-08 | 0.042 | G | 0.235 | *ABO* | ABO blood group (transferase A, alpha 1-3-N-acetylgalactosaminyltransferase; transferase B, alpha 1-3-galactosyltransferase) |
| rs687289 | 9 | 135126927 | intron |  |  | [T/C] | IL-6 (pg/mL) | 2.94E-08 | 0.042 | A | 0.236 | *ABO* | ABO blood group (transferase A, alpha 1-3-N-acetylgalactosaminyltransferase; transferase B, alpha 1-3-galactosyltransferase) |
| rs657152 | 9 | 135129086 | intron |  |  | [A/C] | IL-6 (pg/mL) | 2.03E-08 | 0.041 | A | 0.254 | *ABO* | ABO blood group (transferase A, alpha 1-3-N-acetylgalactosaminyltransferase; transferase B, alpha 1-3-galactosyltransferase) |
| rs507666 | 9 | 135139220 | intron |  |  | [T/C] | IL-6 (pg/mL) | 2.07E-07 | 0.037 | A | 0.150 | *ABO* | ABO blood group (transferase A, alpha 1-3-N-acetylgalactosaminyltransferase; transferase B, alpha 1-3-galactosyltransferase) |
| rs552148 | 9 | 135143302 | flanking_5UTR |  |  | [T/C] | IL-6 (pg/mL) | 7.85E-06 | 0.029 | A | 0.443 | *ABO* | ABO blood group (transferase A, alpha 1-3-N-acetylgalactosaminyltransferase; transferase B, alpha 1-3-galactosyltransferase) |
| rs579459 | 9 | 135143989 | flanking_5UTR |  |  | [T/C] | IL-6 (pg/mL) | 1.66E-07 | 0.036 | G | 0.166 | *ABO* | ABO blood group (transferase A, alpha 1-3-N-acetylgalactosaminyltransferase; transferase B, alpha 1-3-galactosyltransferase) |
| rs633862 | 9 | 135145265 | flanking_5UTR |  |  | [A/G] | IL-6 (pg/mL) | 1.75E-06 | 0.032 | A | 0.323 | *ABO* | ABO blood group (transferase A, alpha 1-3-N-acetylgalactosaminyltransferase; transferase B, alpha 1-3-galactosyltransferase) |
| rs3849221 | 9 | 137168498 | flanking_3UTR |  |  | [T/G] | Estradiol (pg/mL) | 9.85E-07 | 0.031 | A | 0.409 | *OLFM1* | olfactomedin 1 |
| rs34971035 | 9 | 138385417 | coding | SYNON | D108D(NP_434700.2) | [A/G] | MCP-1 (pg/mL) | 4.64E-06 | 0.042 | A | 0.064 | *CARD9* | caspase recruitment domain family, member 9 |
| rs28417902 | 9 | 139208451 | intron |  |  | [A/G] | Testosterone (ng/mL) | 7.38E-06 | 0.025 | A | 0.032 | *TPRN* | taperin |
| rs12571964 | 10 | 2414556 | flanking_3UTR |  |  | [A/G] | Gestational age (wk) | 8.68E-06 | 0.047 | A | 0.210 |  |  |
| rs2165953 | 10 | 4706542 | flanking_3UTR |  |  | [A/G] | Sedentary activity (min/d) | 8.65E-06 | 0.024 | G | 0.153 | *LOC338588* | uncharacterized LOC338588 |
| rs17145612 | 10 | 5958438 | intron |  |  | [T/C] | Head circumference (cm) | 5.54E-06 | 0.013 | A | 0.008 | *ANKRD16* | ankyrin repeat domain 16 |
| rs4749791 | 10 | 8673867 | flanking_5UTR |  |  | [T/C] | Total antioxidants (mM) | 7.78E-06 | 0.025 | A | 0.469 | *LOC338591* | coiled-coil-helix-coiled-coil-helix domain containing 3 pseudogene |
| rs12263766 | 10 | 10036996 | flanking_5UTR |  |  | [A/G] | Urinary free epinephrine (nmol/d) | 2.89E-06 | 0.022 | G | 0.004 | *TCEB1P3* | transcription elongation factor B (SIII), polypeptide 1 pseudogene 3 |
| rs17147135 | 10 | 10124572 | flanking_5UTR |  |  | [A/G] | Vigorous activity (min/d) | 7.68E-06 | 0.030 | G | 0.067 | *TCEB1P3* | transcription elongation factor B (SIII), polypeptide 1 pseudogene 3 |
| rs7097577 | 10 | 10336125 | flanking_3UTR |  |  | [A/C] | Urinary free dopamine (nmol/d) | 6.00E-06 | 0.038 | C | 0.006 | *TCEB1P3* | transcription elongation factor B (SIII), polypeptide 1 pseudogene 3 |
| rs10905868 | 10 | 11014262 | flanking_5UTR |  |  | [T/C] | TSH (µIU/mL) | 3.69E-06 | 0.020 | G | 0.151 | *CUGBP2 -- CELF2* | CUGBP, Elav-like family member 2 |
| rs12265836 | 10 | 11759016 | flanking_5UTR |  |  | [T/G] | AST/ALT | 7.92E-06 | 0.027 | C | 0.106 | *USP6NL* | USP6 N-terminal like |
| rs4750211 | 10 | 12475385 | intron |  |  | [A/G] | RQmax | 5.69E-07 | 0.047 | A | 0.300 | *CAMK1D* | calcium/calmodulin-dependent protein kinase ID |
| rs4747982 | 10 | 12475401 | intron |  |  | [A/G] | RQmax | 9.48E-07 | 0.046 | A | 0.300 | *CAMK1D* | calcium/calmodulin-dependent protein kinase ID |
| rs10906142 | 10 | 12478789 | intron |  |  | [A/G] | Energy storage (kcal/d) | 3.56E-06 | 0.034 | A | 0.236 | *CAMK1D* | calcium/calmodulin-dependent protein kinase ID |
| rs10906142 | 10 | 12478789 | intron |  |  | [A/G] | Fat mass change (kg/y) | 1.36E-06 | 0.038 | A | 0.236 | *CAMK1D* | calcium/calmodulin-dependent protein kinase ID |
| rs10906142 | 10 | 12478789 | intron |  |  | [A/G] | Fat mass deposition (kcal/d) | 1.43E-06 | 0.037 | A | 0.236 | *CAMK1D* | calcium/calmodulin-dependent protein kinase ID |
| rs11259474 | 10 | 15169408 | intron |  |  | [A/G] | Free T3 (pg/mL) | 5.26E-06 | 0.029 | G | 0.109 | *ACBD7* | acyl-CoA binding domain containing 7 |
| rs11254160 | 10 | 16784529 | intron |  |  | [T/C] | Testosterone (ng/mL) | 8.26E-06 | 0.028 | A | 0.138 | *RSU1* | Ras suppressor protein 1 |
| rs11254160 | 10 | 16784529 | intron |  |  | [T/C] | VO2max (mL/min) | 6.21E-06 | 0.041 | A | 0.138 | *RSU1* | Ras suppressor protein 1 |
| rs7916663 | 10 | 16788616 | intron |  |  | [T/C] | Bone mineral content (kg) | 1.71E-06 | 0.036 | A | 0.184 | *RSU1* | Ras suppressor protein 1 |
| rs7916663 | 10 | 16788616 | intron |  |  | [T/C] | Height (cm) | 4.07E-07 | 0.035 | A | 0.184 | *RSU1* | Ras suppressor protein 1 |
| rs7916663 | 10 | 16788616 | intron |  |  | [T/C] | Height z-score (SD) | 5.34E-06 | 0.030 | A | 0.184 | *RSU1* | Ras suppressor protein 1 |
| rs8181477 | 10 | 18822886 | intron |  |  | [T/G] | BMR RQ | 1.19E-06 | 0.041 | C | 0.419 | *CACNB2* | calcium channel, voltage-dependent, beta 2 subunit |
| rs16918253 | 10 | 19445537 | intron |  |  | [T/G] | TG/HDLC (mmol/L) | 7.62E-06 | 0.029 | A | 0.036 | *LOC100652979* | MAM and LDL-receptor class A domain-containing protein C10orf112-like |
| rs7094131 | 10 | 22887445 | 5UTR |  |  | [A/G] | Vigorous activity (%awake time) | 9.49E-06 | 0.031 | G | 0.392 | *PIP4K2A* | phosphatidylinositol-5-phosphate 4-kinase, type II, alpha |
| rs7893600 | 10 | 23474750 | flanking_3UTR |  |  | [T/G] | IL-6 (pg/mL) | 2.60E-06 | 0.037 | A | 0.329 | *MSRB2* | methionine sulfoxide reductase B2 |
| rs11014249 | 10 | 25151916 | flanking_3UTR |  |  | [T/G] | Trunk fat mass (kg) | 2.65E-06 | 0.035 | C | 0.284 | *PRTFDC1* | phosphoribosyl transferase domain containing 1 |
| rs11014249 | 10 | 25151916 | flanking_3UTR |  |  | [T/G] | Trunk fat mass (kg) | 5.64E-06 | 0.032 | C | 0.284 | *PRTFDC1* | phosphoribosyl transferase domain containing 1 |
| rs4749080 | 10 | 26202772 | flanking_5UTR |  |  | [A/G] | IL-6 (pg/mL) | 4.03E-07 | 0.043 | G | 0.268 | *MYO3A* | myosin IIIA |
| rs12413953 | 10 | 26213701 | flanking_5UTR |  |  | [A/G] | IL-6 (pg/mL) | 1.98E-06 | 0.040 | A | 0.283 | *MYO3A* | myosin IIIA |
| rs9787562 | 10 | 26292011 | intron |  |  | [A/G] | IL-6 (pg/mL) | 4.71E-06 | 0.035 | G | 0.266 | *MYO3A* | myosin IIIA |
| rs12573019 | 10 | 26354311 | intron |  |  | [T/C] | IL-6 (pg/mL) | 8.20E-06 | 0.035 | A | 0.276 | *MYO3A* | myosin IIIA |
| rs1339811 | 10 | 26358343 | intron |  |  | [T/C] | IL-6 (pg/mL) | 8.86E-06 | 0.034 | G | 0.276 | *MYO3A* | myosin IIIA |
| rs2096176 | 10 | 26473372 | intron |  |  | [A/G] | Cortisol (ng/mL) | 4.80E-06 | 0.023 | A | 0.330 | *MYO3A* | myosin IIIA |
| rs12764197 | 10 | 26524160 | intron |  |  | [T/C] | IL-6 (pg/mL) | 5.97E-06 | 0.034 | G | 0.304 | *MYO3A* | myosin IIIA |
| rs11006747 | 10 | 28190941 | intron |  |  | [A/C] | Total cysteine (µmol/L) | 7.67E-06 | 0.022 | A | 0.383 | *ARMC4* | armadillo repeat containing 4 |
| rs10763642 | 10 | 28428156 | intron |  |  | [T/C] | Total glutathione (µmol/L) | 6.65E-06 | 0.027 | A | 0.426 | *MPP7* | membrane protein, palmitoylated 7 (MAGUK p55 subfamily member 7) |
| rs2153299 | 10 | 28792112 | flanking_3UTR |  |  | [T/C] | RQmax | 3.32E-06 | 0.043 | G | 0.463 | *RPSAP10* | ribosomal protein SA pseudogene 10 |
| rs28461806 | 10 | 43075760 | intron |  |  | [A/G] | IGFBP-3 (ng/mL) | 2.04E-06 | 0.029 | G | 0.049 | *RASGEF1A* | RasGEF domain family, member 1A |
| rs28461806 | 10 | 43075760 | intron |  |  | [A/G] | MCP-1 (pg/mL) | 4.58E-08 | 0.053 | G | 0.049 | *RASGEF1A* | RasGEF domain family, member 1A |
| rs7924201 | 10 | 43776035 | flanking_3UTR |  |  | [T/G] | Birth weight (kg) | 9.09E-06 | 0.062 | A | 0.284 | *C10orf136* | chromosome 10 open reading frame 136 |
| rs898549 | 10 | 43778720 | flanking_3UTR |  |  | [A/G] | Birth weight (kg) | 6.02E-06 | 0.062 | A | 0.278 | *C10orf136* | chromosome 10 open reading frame 136 |
| rs2047009 | 10 | 43859919 | flanking_3UTR |  |  | [A/C] | Birth weight (kg) | 8.86E-06 | 0.056 | C | 0.268 | *C10orf136* | chromosome 10 open reading frame 136 |
| rs293314 | 10 | 52912709 | flanking_3UTR |  |  | [T/C] | Sitting height (cm) | 6.53E-06 | 0.027 | G | 0.056 | *PRKG1* | protein kinase, cGMP-dependent, type I |
| rs2790232 | 10 | 59655677 | intron |  |  | [T/C] | Arm span (cm) | 8.11E-06 | 0.013 | A | 0.029 | *IPMK* | inositol polyphosphate multikinase |
| rs11006263 | 10 | 60225584 | intron |  |  | [A/G] | Height (cm) | 7.00E-06 | 0.028 | G | 0.030 | *BICC1* | bicaudal C homolog 1 (Drosophila) |
| rs2814021 | 10 | 63031993 | flanking_5UTR |  |  | [T/C] | Fat free mass change (kg/y) | 6.75E-06 | 0.036 | G | 0.204 | *C10orf107* | chromosome 10 open reading frame 107 |
| rs7909235 | 10 | 68044090 | intron |  |  | [T/G] | ALT/AST | 7.78E-06 | 0.028 | A | 0.193 | *CTNNA3* | catenin (cadherin-associated protein), alpha 3 |
| rs1466576 | 10 | 72358377 | flanking_5UTR |  |  | [A/G] | Total glutathione (µmol/L) | 8.25E-06 | 0.026 | G | 0.472 | *PCBD1* | pterin-4 alpha-carbinolamine dehydratase/dimerization cofactor of hepatocyte nuclear factor 1 alpha |
| rs1891159 | 10 | 72465883 | flanking_5UTR |  |  | [A/G] | HOMA-IR | 5.88E-06 | 0.034 | A | 0.491 | *PCBD1* | pterin-4 alpha-carbinolamine dehydratase/dimerization cofactor of hepatocyte nuclear factor 1 alpha |
| rs1891159 | 10 | 72465883 | flanking_5UTR |  |  | [A/G] | Insulin (µU/mL) | 2.72E-06 | 0.036 | A | 0.491 | *PCBD1* | pterin-4 alpha-carbinolamine dehydratase/dimerization cofactor of hepatocyte nuclear factor 1 alpha |
| rs1084004 | 10 | 72791951 | coding | SYNON | G336G(NP_060814.4) | [A/G] | HOMA-IR | 2.30E-06 | 0.027 | A | 0.377 | *SLC29A3* | solute carrier family 29 (nucleoside transporters), member 3 |
| rs1084004 | 10 | 72791951 | coding | SYNON | G336G(NP_060814.4) | [A/G] | Insulin (µU/mL) | 8.19E-07 | 0.029 | A | 0.377 | *SLC29A3* | solute carrier family 29 (nucleoside transporters), member 3 |
| rs12261589 | 10 | 79210841 | flanking_3UTR |  |  | [T/C] | Sedentary activity (%awake time) | 4.23E-06 | 0.042 | A | 0.012 | *DLG5* | discs, large homolog 5 (Drosophila) |
| rs7915137 | 10 | 84860056 | flanking_3UTR |  |  | [A/G] | Methionine (µmol/L) | 6.91E-06 | 0.032 | G | 0.333 | *NRG3* | neuregulin 3 |
| rs11201253 | 10 | 86578882 | flanking_3UTR |  |  | [A/G] | TSH (µIU/mL) | 6.40E-06 | 0.020 | A | 0.162 | *RPS3AP5* | ribosomal protein S3A pseudogene 5 |
| rs7920888 | 10 | 90808357 | flanking_3UTR |  |  | [A/G] | BMI z-score (SD) | 5.06E-06 | 0.027 | A | 0.494 | *FAS* | Fas (TNF receptor superfamily, member 6) |
| rs7920888 | 10 | 90808357 | flanking_3UTR |  |  | [A/G] | Hip circumference (cm) | 6.98E-06 | 0.025 | A | 0.494 | *FAS* | Fas (TNF receptor superfamily, member 6) |
| rs7920888 | 10 | 90808357 | flanking_3UTR |  |  | [A/G] | Waist circumference (cm) | 9.22E-06 | 0.025 | A | 0.494 | *FAS* | Fas (TNF receptor superfamily, member 6) |
| rs7920888 | 10 | 90808357 | flanking_3UTR |  |  | [A/G] | Weight z-score (SD) | 6.81E-06 | 0.026 | A | 0.494 | *FAS* | Fas (TNF receptor superfamily, member 6) |
| rs579342 | 10 | 97219752 | intron |  |  | [A/G] | Height z-score change (SD/y) | 3.48E-06 | 0.031 | A | 0.041 | *SORBS1* | sorbin and SH3 domain containing 1 |
| rs12247897 | 10 | 97241752 | intron |  |  | [T/C] | Total energy expenditure RQ | 9.74E-06 | 0.031 | A | 0.028 | *SORBS1* | sorbin and SH3 domain containing 1 |
| rs3824789 | 10 | 98790089 | intron |  |  | [A/G] | Cystathionine (µmol/L) | 6.13E-06 | 0.029 | A | 0.248 | *SLIT1* | slit homolog 1 (Drosophila) |
| rs12767760 | 10 | 99284787 | intron |  |  | [T/C] | Total cysteine (µmol/L) | 6.51E-06 | 0.018 | G | 0.364 | *UBTD1* | ubiquitin domain containing 1 |
| rs2147901 | 10 | 100155486 | intron |  |  | [T/G] | Urinary creatinine (mmol/d) | 1.32E-06 | 0.037 | A | 0.364 | *PYROXD2* | pyridine nucleotide-disulphide oxidoreductase domain 2 |
| rs12763326 | 10 | 100157386 | coding | SYNON | R86R(NP_116098.1) | [T/C] | Urinary creatinine (mmol/d) | 3.55E-06 | 0.033 | A | 0.352 | *PYROXD2* | pyridine nucleotide-disulphide oxidoreductase domain 2 |
| rs7923729 | 10 | 100159455 | intron |  |  | [T/C] | Urinary creatinine (mmol/d) | 2.64E-06 | 0.034 | A | 0.355 | *PYROXD2* | pyridine nucleotide-disulphide oxidoreductase domain 2 |
| rs6584202 | 10 | 100160373 | intron |  |  | [T/C] | Urinary creatinine (mmol/d) | 4.04E-07 | 0.041 | A | 0.367 | *PYROXD2* | pyridine nucleotide-disulphide oxidoreductase domain 2 |
| rs2147900 | 10 | 100164711 | intron |  |  | [A/G] | Urinary creatinine (mmol/d) | 2.20E-06 | 0.035 | G | 0.355 | *PYROXD2* | pyridine nucleotide-disulphide oxidoreductase domain 2 |
| rs1061437 | 10 | 100166144 | 3UTR |  |  | [A/G] | Urinary creatinine (mmol/d) | 4.54E-06 | 0.033 | A | 0.354 | *HPS1* | Hermansky-Pudlak syndrome 1 |
| rs7921146 | 10 | 100176591 | intron |  |  | [A/G] | Urinary creatinine (mmol/d) | 4.21E-06 | 0.035 | A | 0.324 | *HPS1* | Hermansky-Pudlak syndrome 1 |
| rs2801405 | 10 | 100573905 | intron |  |  | [A/G] | Urinary free norepinephrine (nmol/d) | 8.95E-06 | 0.032 | G | 0.330 | *HPSE2* | heparanase 2 |
| rs2281636 | 10 | 101482393 | intron |  |  | [A/C] | LDL (mg/dL) | 6.21E-06 | 0.030 | A | 0.336 | *COX15* | COX15 homolog, cytochrome c oxidase assembly protein (yeast) |
| rs7913190 | 10 | 101488386 | intron |  |  | [A/C] | LDL (mg/dL) | 7.46E-06 | 0.030 | C | 0.330 | *CUTC* | cutC copper transporter homolog (E. coli) |
| rs2804419 | 10 | 101499103 | intron |  |  | [T/C] | LDL (mg/dL) | 7.77E-06 | 0.029 | A | 0.337 | *CUTC* | cutC copper transporter homolog (E. coli) |
| rs2278842 | 10 | 102046735 | coding | NONSYN | V393I(NP_057196.2) | [T/C] | Total antioxidants (mM) | 3.45E-06 | 0.027 | A | 0.145 | *PKD2L1* | polycystic kidney disease 2-like 1 |
| rs17112901 | 10 | 102056175 | intron |  |  | [A/G] | Total antioxidants (mM) | 1.87E-06 | 0.029 | A | 0.163 | *PKD2L1* | polycystic kidney disease 2-like 1 |
| rs3981351 | 10 | 115505110 | intron |  |  | [A/G] | Free T3 (pg/mL) | 1.32E-06 | 0.038 | G | 0.276 | *C10orf81* | chromosome 10 open reading frame 81 |
| rs941853 | 10 | 116189165 | intron |  |  | [A/G] | BMI z-score change (SD/y) | 5.53E-06 | 0.030 | A | 0.189 | *ABLIM1* | actin binding LIM protein 1 |
| rs384627 | 10 | 119326736 | flanking_3UTR |  |  | [T/C] | TBF-ß1 (pg/mL) | 5.17E-06 | 0.026 | A | 0.006 | *EMX2* | empty spiracles homeobox 2 |
| rs1397615 | 10 | 120956329 | intron |  |  | [T/C] | IGFBP-3 (ng/mL) | 1.70E-06 | 0.030 | A | 0.044 | *GRK5* | G protein-coupled receptor kinase 5 |
| rs2919009 | 10 | 122581363 | flanking_5UTR |  |  | [A/G] | Diet protein (%energy) | 4.75E-06 | 0.035 | A | 0.120 | *BRWD2 -- WDR11* | WD repeat domain 11 |
| rs11200392 | 10 | 123841817 | intron |  |  | [T/C] | Height change (cm/y) | 2.51E-06 | 0.036 | A | 0.368 | *TACC2* | transforming, acidic coiled-coil containing protein 2 |
| rs3740540 | 10 | 126284157 | intron |  |  | [A/G] | Sitting height (cm) | 4.90E-06 | 0.028 | A | 0.287 | *LHPP* | phospholysine phosphohistidine inorganic pyrophosphate phosphatase |
| rs10510138 | 10 | 126428009 | flanking_5UTR |  |  | [A/G] | TSH (µIU/mL) | 7.64E-06 | 0.022 | A | 0.052 | *FAM53B* | family with sequence similarity 53, member B |
| rs2306702 | 10 | 126436463 | flanking_3UTR |  |  | [A/C] | TSH (µIU/mL) | 7.64E-06 | 0.022 | C | 0.052 | *METTL10* | methyltransferase like 10 |
| rs989507 | 10 | 127125828 | flanking_5UTR |  |  | [A/G] | Urinary creatinine (mmol/d) | 1.88E-06 | 0.033 | A | 0.188 |  |  |
| rs4750827 | 10 | 132437174 | flanking_5UTR |  |  | [A/G] | Weight change (kg/y) | 5.18E-06 | 0.028 | A | 0.184 |  |  |
| rs4750829 | 10 | 132437251 | flanking_5UTR |  |  | [A/G] | Energy storage (kcal/d) | 9.32E-06 | 0.030 | A | 0.185 |  |  |
| rs4750829 | 10 | 132437251 | flanking_5UTR |  |  | [A/G] | Weight change (kg/y) | 4.35E-06 | 0.029 | A | 0.185 |  |  |
| rs7070213 | 10 | 132451984 | flanking_5UTR |  |  | [T/C] | Weight change (kg/y) | 8.35E-06 | 0.026 | A | 0.153 |  |  |
| rs2249694 | 10 | 135202143 | intron |  |  | [A/G] | Arm span (cm) | 9.53E-06 | 0.033 | A | 0.225 | *CYP2E1* | cytochrome P450, family 2, subfamily E, polypeptide 1 |
| rs2249694 | 10 | 135202143 | intron |  |  | [A/G] | Height z-score (SD) | 3.26E-06 | 0.032 | A | 0.225 | *CYP2E1* | cytochrome P450, family 2, subfamily E, polypeptide 1 |
| rs2249695 | 10 | 135202158 | intron |  |  | [T/C] | Arm span (cm) | 9.53E-06 | 0.033 | A | 0.225 | *CYP2E1* | cytochrome P450, family 2, subfamily E, polypeptide 1 |
| rs2249695 | 10 | 135202158 | intron |  |  | [T/C] | Height z-score (SD) | 3.26E-06 | 0.032 | A | 0.225 | *CYP2E1* | cytochrome P450, family 2, subfamily E, polypeptide 1 |
| rs35694355 | 11 | 821883 | 3UTR |  |  | [T/C] | Head circumference (cm) | 2.54E-06 | 0.030 | A | 0.005 | *EFCAB4A* | EF-hand calcium binding domain 4A |
| rs2521263 | 11 | 2265338 | flanking_3UTR |  |  | [A/G] | Fat free mass change (kg/y) | 4.89E-06 | 0.033 | A | 0.309 | *C11orf21* | chromosome 11 open reading frame 21 |
| rs2074018 | 11 | 2266210 | flanking_3UTR |  |  | [A/T] | Fat free mass change (kg/y) | 5.15E-06 | 0.033 | A | 0.312 | *C11orf21* | chromosome 11 open reading frame 21 |
| rs10160399 | 11 | 4473152 | flanking_3UTR |  |  | [A/G] | Diastolic blood pressure (mmHg) | 1.95E-06 | 0.028 | A | 0.042 | *OR52K1* | olfactory receptor, family 52, subfamily K, member 1 |
| rs12416860 | 11 | 4519794 | flanking_5UTR |  |  | [A/G] | TNF-α (pg/mL) | 4.15E-06 | 0.026 | G | 0.073 | *OR52M1* | olfactory receptor, family 52, subfamily M, member 1 |
| rs16912210 | 11 | 5220429 | intron |  |  | [T/C] | Fat free mass change (kg/y) | 3.46E-07 | 0.046 | G | 0.080 | *HBBP1* | hemoglobin, beta pseudogene |
| rs16912210 | 11 | 5220429 | intron |  |  | [T/C] | Protein deposition (kcal/d) | 1.18E-06 | 0.040 | G | 0.080 | *HBBP1* | hemoglobin, beta pseudogene |
| rs16912210 | 11 | 5220429 | intron |  |  | [T/C] | Protein (kg/y) | 1.18E-06 | 0.040 | G | 0.080 | *HBBP1* | hemoglobin, beta pseudogene |
| rs2727405 | 11 | 13047518 | flanking_3UTR |  |  | [T/C] | Bike energy expenditure (kcal/min) | 7.85E-06 | 0.033 | A | 0.323 | *RASSF10* | Ras association (RalGDS/AF-6) domain family (N-terminal) member 10 |
| rs16931632 | 11 | 15576519 | flanking_3UTR |  |  | [T/C] | Diet protein (g/d) | 7.34E-06 | 0.028 | G | 0.051 | *INSC* | inscuteable homolog (Drosophila) |
| rs17439299 | 11 | 15577162 | flanking_3UTR |  |  | [A/G] | Diet protein (g/d) | 6.19E-06 | 0.032 | G | 0.044 | *INSC* | inscuteable homolog (Drosophila) |
| rs2351059 | 11 | 15577483 | flanking_3UTR |  |  | [T/G] | Diet protein (g/d) | 7.34E-06 | 0.028 | C | 0.051 | *INSC* | inscuteable homolog (Drosophila) |
| rs10500856 | 11 | 19541078 | flanking_5UTR |  |  | [T/C] | Height z-score change (SD/y) | 7.17E-06 | 0.029 | G | 0.009 | *NAV2* | neuron navigator 2 |
| rs7128099 | 11 | 20313020 | flanking_5UTR |  |  | [A/G] | Adiponectin (ng/mL) | 2.94E-06 | 0.028 | G | 0.335 | *HTATIP2* | HIV-1 Tat interactive protein 2, 30kDa |
| rs11025523 | 11 | 20334700 | flanking_5UTR |  |  | [T/C] | Sedentary&light activity (min/d) | 6.50E-06 | 0.035 | A | 0.091 | *HTATIP2* | HIV-1 Tat interactive protein 2, 30kDa |
| rs2241941 | 11 | 20579599 | coding | SYNON | L118L(NP_004202.2) | [A/G] | Methionine (µmol/L) | 7.57E-06 | 0.029 | A | 0.339 | *SLC6A5* | solute carrier family 6 (neurotransmitter transporter, glycine), member 5 |
| rs10833583 | 11 | 21625869 | flanking_3UTR |  |  | [T/C] | IGFBP-1 (ng/mL) | 7.33E-06 | 0.032 | A | 0.061 | *NELL1* | NEL-like 1 (chicken) |
| rs2051457 | 11 | 21633918 | flanking_3UTR |  |  | [A/C] | Sleep duration (min/d) | 4.46E-06 | 0.027 | A | 0.096 | *NELL1* | NEL-like 1 (chicken) |
| rs11601602 | 11 | 28190344 | intron |  |  | [T/C] | Dinner intake (kcal) | 8.84E-06 | 0.043 | G | 0.142 | *METTL15* | methyltransferase like 15 |
| rs3026354 | 11 | 31787233 | flanking_3UTR |  |  | [T/C] | Head circumference (cm) | 9.87E-06 | 0.026 | G | 0.241 | *PAX6* | paired box 6 |
| rs286905 | 11 | 34619537 | intron |  |  | [A/G] | Estradiol (pg/mL) | 4.14E-06 | 0.029 | A | 0.311 | *EHF* | ets homologous factor |
| rs1488665 | 11 | 45484692 | flanking_3UTR |  |  | [T/G] | Urinary free norepinephrine (nmol/d) | 4.93E-06 | 0.032 | A | 0.105 | *FLJ41423* | uncharacterized LOC399886 |
| rs17150243 | 11 | 55776501 | coding | NONSYN | W84G(NP_001004747.1) | [T/G] | Total cholesterol (mg/dL) | 9.00E-06 | 0.029 | C | 0.158 | *OR5T3* | olfactory receptor, family 5, subfamily T, member 3 |
| rs28362944 | 11 | 57122299 | UTR |  |  | [A/G] | Insulin (µU/mL) | 8.99E-06 | 0.028 | G | 0.022 | *SERPING1* | serpin peptidase inhibitor, clade G (C1 inhibitor), member 1 |
| rs7124676 | 11 | 64069867 | flanking_5UTR |  |  | [A/G] | Urinary free dopamine (nmol/d) | 3.18E-06 | 0.036 | A | 0.232 | *SLC22A11* | solute carrier family 22 (organic anion/urate transporter), member 11 |
| rs3759053 | 11 | 64079656 | flanking_5UTR |  |  | [T/C] | Urinary free dopamine (nmol/d) | 5.31E-06 | 0.032 | A | 0.272 | *SLC22A11* | solute carrier family 22 (organic anion/urate transporter), member 11 |
| rs2924679 | 11 | 68313358 | intron |  |  | [A/G] | Fat oxidation (%NPEE) | 3.97E-06 | 0.033 | A | 0.094 | *CPT1A* | carnitine palmitoyltransferase 1A (liver) |
| rs75677837 | 11 | 68317356 | intron |  |  | [T/C] | Fat oxidation (%NPEE) | 5.34E-06 | 0.032 | A | 0.085 | *CPT1A* | carnitine palmitoyltransferase 1A (liver) |
| rs35198051 | 11 | 70522852 | flanking_5UTR |  |  | [T/C] | Estradiol (pg/mL) | 1.24E-06 | 0.030 | A | 0.262 | *SHANK2* | SH3 and multiple ankyrin repeat domains 2 |
| rs1723838 | 11 | 73154693 | flanking_5UTR |  |  | [A/G] | Diet carbohydrate (%energy) | 3.76E-06 | 0.024 | A | 0.032 | *RAB6A* | RAB6A, member RAS oncogene family |
| rs1723845 | 11 | 73175572 | flanking_5UTR |  |  | [A/G] | Diet carbohydrate (%energy) | 3.76E-06 | 0.024 | G | 0.032 | *MRPL48* | mitochondrial ribosomal protein L48 |
| rs1151200 | 11 | 78670334 | flanking_5UTR |  |  | [T/C] | Birth weight (kg) | 4.10E-07 | 0.058 | G | 0.419 | *ODZ4* | odd Oz/ten-m homolog 4 (Drosophila) [Homo sapiens |
| rs499949 | 11 | 78670855 | flanking_5UTR |  |  | [A/G] | Birth weight (kg) | 7.80E-07 | 0.061 | A | 0.449 | *ODZ4* | odd Oz/ten-m homolog 4 (Drosophila) [Homo sapiens |
| rs7931512 | 11 | 78697937 | flanking_5UTR |  |  | [T/C] | Birth weight (kg) | 2.39E-06 | 0.038 | G | 0.147 | *ODZ4* | odd Oz/ten-m homolog 4 (Drosophila) [Homo sapiens |
| rs10501481 | 11 | 80214984 | flanking_5UTR |  |  | [T/C] | Free T3 (pg/mL) | 9.64E-06 | 0.028 | G | 0.054 | *LOC729790* | ADP-ribosylation factor-like 6 interacting protein 1 pseudogene |
| rs10792665 | 11 | 82327416 | flanking_3UTR |  |  | [A/C] | Total T3 (ng/dL) | 5.63E-06 | 0.028 | C | 0.443 | *C11orf82* | chromosome 11 open reading frame 8 |
| rs1943345 | 11 | 82553918 | intron |  |  | [T/G] | Moderate activity (%awake time) | 7.12E-06 | 0.031 | C | 0.239 | *PCF11* | PCF11, cleavage and polyadenylation factor subunit, homolog (S. cerevisiae) |
| rs2116483 | 11 | 82934516 | intron |  |  | [A/G] | Energy intake (kcal/d) | 7.74E-06 | 0.024 | A | 0.416 | *DLG2* | discs, large homolog 2 (Drosophila) |
| rs10830265 | 11 | 88752298 | intron |  |  | [T/C] | Total homocysteine (µmol/L) | 7.89E-06 | 0.019 | A | 0.187 | *NOX4* | NADPH oxidase 4 |
| rs317150 | 11 | 88763416 | intron |  |  | [A/G] | Total homocysteine (µmol/L) | 8.47E-06 | 0.019 | A | 0.196 | *NOX4* | NADPH oxidase 4 |
| rs1387153 | 11 | 92313476 | intron |  |  | [A/G] | Glucose (mg/dL) | 1.91E-06 | 0.040 | A | 0.233 | *LOC642791* | eukaryotic translation elongation factor 1 alpha 2 pseudogene |
| rs10830963 | 11 | 92348358 | intron |  |  | [G/C] | Glucose (mg/dL) | 3.72E-08 | 0.048 | G | 0.205 | *MTNR1B* | melatonin receptor 1B |
| rs12296063 | 11 | 92560050 | intron |  |  | [T/C] | Folate (nmol/L) | 1.56E-06 | 0.026 | A | 0.003 | *SLC36A4* | solute carrier family 36 (proton/amino acid symporter), member 4 [Homo sapiens] |
| rs12098946 | 11 | 92732785 | intron |  |  | [T/C] | Folate (nmol/L) | 3.94E-06 | 0.023 | A | 0.005 | *CCDC67* | coiled-coil domain containing 67 [Homo sapiens] |
| rs11825709 | 11 | 93343697 | flanking_5UTR |  |  | [A/G] | Folate (nmol/L) | 4.37E-06 | 0.024 | G | 0.034 | *HEPHL1* | hephaestin-like 1 |
| rs11217223 | 11 | 98228426 | flanking_5UTR |  |  | [A/G] | Moderate activity (%awake time) | 1.31E-06 | 0.040 | G | 0.090 | *CNTN5* | contactin 5 |
| rs11217223 | 11 | 98228426 | flanking_5UTR |  |  | [A/G] | Moderate&vigorous activity (min/d) | 6.28E-06 | 0.034 | G | 0.090 | *CNTN5* | contactin 5 |
| rs12364992 | 11 | 106028434 | flanking_3UTR |  |  | [T/C] | Hip circumference change (cm/y) | 6.22E-06 | 0.026 | G | 0.176 | *GUCY1A2* | guanylate cyclase 1, soluble, alpha 2 |
| rs11213935 | 11 | 110916818 | coding | NONSYN | V14M(NP_849156.1) | [T/C] | IGFBP-3 (ng/mL) | 3.64E-06 | 0.028 | A | 0.078 | *LAYN* | layilin |
| rs220836 | 11 | 114807081 | intron |  |  | [T/C] | AST (U/L) | 6.42E-06 | 0.031 | G | 0.160 | *CADM1* | cell adhesion molecule 1 |
| rs2160669 | 11 | 116152817 | flanking_3UTR |  |  | [A/G] | TG/HDLC (mmol/L) | 8.66E-06 | 0.028 | G | 0.168 | *ZNF259* | zinc finger protein 259 |
| rs2160669 | 11 | 116152817 | flanking_3UTR |  |  | [A/G] | Triglycerides (mg/dL) | 5.10E-08 | 0.041 | G | 0.168 | *ZNF259* | zinc finger protein 259 |
| rs3741298 | 11 | 116162771 | intron |  |  | [A/G] | Triglycerides (mg/dL) | 2.47E-08 | 0.043 | G | 0.480 | *ZNF259* | zinc finger protein 259 |
| rs2266788 | 11 | 116165896 | 3UTR |  |  | [A/G] | TG/HDLC (mmol/L) | 8.92E-06 | 0.028 | G | 0.163 | *APOA5* | apolipoprotein A-V |
| rs2266788 | 11 | 116165896 | 3UTR |  |  | [A/G] | Triglycerides (mg/dL) | 4.82E-08 | 0.042 | G | 0.163 | *APOA5* | apolipoprotein A-V |
| rs651821 | 11 | 116167789 | 5UTR |  |  | [T/C] | Triglycerides (mg/dL) | 5.30E-07 | 0.035 | G | 0.165 | *APOA5* | apolipoprotein A-V |
| rs662799 | 11 | 116168917 | flanking_5UTR |  |  | [T/C] | Triglycerides (mg/dL) | 5.43E-07 | 0.035 | G | 0.166 | *APOA5* | apolipoprotein A-V |
| rs58603829 | 11 | 117356262 | flanking_5UTR |  |  | [A/G] | Folate (nmol/L) | 6.09E-06 | 0.028 | A | 0.028 | *IL10RA* | interleukin 10 receptor, alpha |
| rs2229115 | 11 | 117374801 | coding | SYNON | T324T(NP_001549.1) | [T/C] | Folate (nmol/L) | 6.09E-06 | 0.028 | A | 0.028 | *IL10RA* | interleukin 10 receptor, alpha |
| rs1893767 | 11 | 123461676 | flanking_5UTR |  |  | [A/G] | Height (cm) | 3.54E-06 | 0.036 | G | 0.126 | *LOH11CR2A* | von Willebrand factor A domain containing 5A |
| rs1893767 | 11 | 123461676 | flanking_5UTR |  |  | [A/G] | Sitting height (cm) | 9.31E-06 | 0.029 | G | 0.126 | *LOH11CR2A* | von Willebrand factor A domain containing 5A |
| rs7112365 | 11 | 124782704 | intron |  |  | [T/C] | IGFBP-3 (ng/mL) | 1.85E-06 | 0.029 | A | 0.070 | *PKNOX2* | PBX/knotted 1 homeobox 2 |
| rs7117932 | 11 | 127942163 | flanking_5UTR |  |  | [A/G] | MCP-1 (pg/mL) | 7.82E-06 | 0.026 | A | 0.287 | *ETS1* | v-ets erythroblastosis virus E26 oncogene homolog 1 |
| rs10894147 | 11 | 129204066 | intron |  |  | [T/C] | Weight z-score change (SD/y) | 7.13E-06 | 0.031 | A | 0.104 | *TMEM45B* | transmembrane protein 45B |
| rs10894239 | 11 | 130134332 | flanking_3UTR |  |  | [T/G] | Waist circumference change (cm/y) | 5.53E-06 | 0.032 | A | 0.466 | *C11orf44* | chromosome 11 open reading frame 44 |
| rs11603690 | 11 | 130830379 | flanking_3UTR |  |  | [T/C] | Free T4 (ng/dL) | 9.20E-06 | 0.036 | G | 0.025 | *NTM* | neurotrimin |
| rs11601906 | 11 | 130830456 | flanking_3UTR |  |  | [A/G] | Free T4 (ng/dL) | 1.67E-06 | 0.041 | A | 0.021 | *NTM* | neurotrimin |
| rs1682859 | 11 | 133305429 | intron |  |  | [T/G] | Urinary free dopamine (nmol/d) | 1.22E-06 | 0.037 | C | 0.480 | *IGSF9B* | immunoglobulin superfamily, member 9B |
| rs478881 | 11 | 133644990 | flanking_3UTR |  |  | [A/G] | Cystathionine (µmol/L) | 6.95E-06 | 0.028 | A | 0.141 | *ACAD8* | acyl-CoA dehydrogenase family, member 8 |
| rs478881 | 11 | 133644990 | flanking_3UTR |  |  | [A/G] | Insulin (µU/mL) | 6.74E-06 | 0.030 | A | 0.141 | *ACAD8* | acyl-CoA dehydrogenase family, member 8 |
| rs10505725 | 12 | 1926624 | intron |  |  | [A/G] | IGF-1 free (ng/mL) | 7.46E-06 | 0.032 | A | 0.280 | *DCP1B* | DCP1 decapping enzyme homolog B (S. cerevisiae) |
| rs12827476 | 12 | 4176144 | flanking_5UTR |  |  | [A/G] | IGF-1 free (ng/mL) | 9.33E-06 | 0.032 | A | 0.452 | *CCND2* | cyclin D2 |
| rs7307889 | 12 | 5851614 | intron |  |  | [T/C] | Calorimeter activity (counts/d) | 2.66E-06 | 0.041 | A | 0.026 | *ANO2* | anoctamin 2 |
| rs10849441 | 12 | 6305832 | intron |  |  | [T/C] | Urinary free dopamine: creatinine | 8.99E-06 | 0.026 | G | 0.352 | *PLEKHG6* | pleckstrin homology domain containing, family G (with RhoGef domain) member 6 |
| rs10771515 | 12 | 9355073 | flanking_5UTR |  |  | [T/C] | RQmax | 9.47E-06 | 0.041 | A | 0.049 | *PZP* | pregnancy-zone protein |
| rs10743889 | 12 | 10412656 | flanking_3UTR |  |  | [T/G] | VO2max (mL/min) | 6.20E-06 | 0.039 | C | 0.400 | *KLRK1* | killer cell lectin-like receptor subfamily K, member 1 |
| rs10841287 | 12 | 19671060 | flanking_3UTR |  |  | [A/G] | Sedentary&light activity (min/d) | 2.24E-06 | 0.029 | G | 0.165 | *AEBP2* | AE binding protein 2 |
| rs11046207 | 12 | 21887312 | intron |  |  | [A/G] | Sedentary activity (min/d) | 5.08E-06 | 0.038 | G | 0.023 | *ABCC9* | ATP-binding cassette, sub-family C (CFTR/MRP), member 9 |
| rs10843215 | 12 | 28635908 | flanking_3UTR |  |  | [A/G] | BMI z-score change (SD/y) | 7.76E-06 | 0.028 | G | 0.309 | *CCDC91* | coiled-coil domain containing 91 |
| rs4931594 | 12 | 32041510 | flanking_3UTR |  |  | [A/C] | Total T4 (µg/dL) | 5.13E-06 | 0.032 | C | 0.242 | *C12orf35* | chromosome 12 open reading frame 35 |
| rs1144713 | 12 | 32152506 | intron |  |  | [A/G] | Urinary free norepinephrine: creatinine | 7.10E-06 | 0.024 | A | 0.230 | *BICD1* | bicaudal D homolog 1 (Drosophila) |
| rs1454933 | 12 | 32908310 | intron |  |  | [T/G] | Energy storage (kcal/d) | 2.92E-06 | 0.036 | C | 0.169 | *PKP2* | plakophilin 2 |
| rs1454933 | 12 | 32908310 | intron |  |  | [T/G] | Fat mass change (kg/y) | 9.14E-06 | 0.033 | C | 0.169 | *PKP2* | plakophilin 2 |
| rs1454933 | 12 | 32908310 | intron |  |  | [T/G] | Fat mass deposition (kcal/d) | 3.33E-06 | 0.036 | C | 0.169 | *PKP2* | plakophilin 2 |
| rs10467147 | 12 | 39053629 | flanking_3UTR |  |  | [T/C] | C-peptide (ng/mL) | 4.71E-06 | 0.033 | A | 0.196 | *LRRK2* | leucine-rich repeat kinase 2 |
| rs1167125 | 12 | 41720549 | flanking_3UTR |  |  | [A/C] | Diet fat (g/d) | 4.97E-06 | 0.028 | C | 0.042 | *ADAMTS20* | ADAM metallopeptidase with thrombospondin type 1 motif, 20 |
| rs7307902 | 12 | 46209283 | flanking_3UTR |  |  | [A/G] | Dinner intake, adj TEE (kcal) | 1.16E-06 | 0.047 | G | 0.243 |  |  |
| rs75365750 | 12 | 46473521 | coding |  |  | [T/C] | MCP-1 (pg/mL) | 5.27E-06 | 0.037 | G | 0.043 | *HDAC7* | histone deacetylase 7 |
| rs7305290 | 12 | 48627732 | flanking_5UTR |  |  | [A/G] | Height change (cm/y) | 9.58E-06 | 0.031 | G | 0.190 | *AQP2* | aquaporin 2 (collecting duct) |
| rs3951439 | 12 | 50013863 | intron |  |  | [A/G] | TBF-ß1 (pg/mL) | 6.17E-06 | 0.027 | G | 0.006 | *CELA1* | chymotrypsin-like elastase family, member 1 |
| rs2272306 | 12 | 51840550 | intron |  |  | [A/G] | Leptin (ng/mL) | 1.54E-06 | 0.035 | A | 0.134 | *CSAD* | cysteine sulfinic acid decarboxylase |
| rs11574546 | 12 | 51871138 | flanking_3UTR |  |  | [A/G] | Leptin (ng/mL) | 1.63E-06 | 0.035 | G | 0.131 | *ITGB7* | integrin, beta 7 |
| rs1629826 | 12 | 53195411 | intron |  |  | [T/C] | Urinary free epinephrine (nmol/d) | 2.94E-06 | 0.035 | G | 0.319 | *NCKAP1L* | NCK-associated protein 1-like |
| rs34379766 | 12 | 54760410 | coding | NONSYN | S20Y(NP_001005915.1) | [A/C] | IGFBP-3 (ng/mL) | 4.50E-07 | 0.033 | A | 0.064 | *ERBB3* | v-erb-b2 erythroblastic leukemia viral oncogene homolog 3 |
| rs1795708 | 12 | 57036947 | flanking_5UTR |  |  | [A/G] | Diet protein (g/d) | 3.76E-06 | 0.035 | G | 0.178 | *ST13P8* | suppression of tumorigenicity 13 (colon carcinoma) (Hsp70 interacting protein) pseudogene 8 |
| rs7974425 | 12 | 59021440 | flanking_3UTR |  |  | [A/G] | Hip circumference (cm) | 5.76E-06 | 0.028 | G | 0.005 |  |  |
| rs7974425 | 12 | 59021440 | flanking_3UTR |  |  | [A/G] | Weight (kg) | 5.96E-06 | 0.027 | G | 0.005 |  |  |
| rs7974425 | 12 | 59021440 | flanking_3UTR |  |  | [A/G] | Weight z-score (SD) | 8.83E-06 | 0.028 | G | 0.005 |  |  |
| rs2655898 | 12 | 59096919 | flanking_3UTR |  |  | [T/C] | Fat mass deposition (kcal/d) | 7.87E-06 | 0.031 | G | 0.246 |  |  |
| rs7304109 | 12 | 60484742 | intron |  |  | [T/C] | BMI z-score (SD) | 1.26E-06 | 0.031 | G | 0.293 | *FAM19A2* | family with sequence similarity 19 (chemokine (C-C motif)-like), member A2 |
| rs1869890 | 12 | 60499668 | intron |  |  | [T/C] | BMI z-score (SD) | 1.04E-06 | 0.031 | A | 0.290 | *FAM19A2* | family with sequence similarity 19 (chemokine (C-C motif)-like), member A2 |
| rs2198776 | 12 | 60521100 | intron |  |  | [T/C] | BMI z-score (SD) | 4.73E-07 | 0.033 | A | 0.284 | *FAM19A2* | family with sequence similarity 19 (chemokine (C-C motif)-like), member A2 |
| rs2198776 | 12 | 60521100 | intron |  |  | [T/C] | Weight z-score (SD) | 4.47E-06 | 0.026 | A | 0.284 | *FAM19A2* | family with sequence similarity 19 (chemokine (C-C motif)-like), member A2 |
| rs549433 | 12 | 61467083 | intron |  |  | [A/G] | Total energy expenditure (kcal/d) | 3.73E-06 | 0.032 | G | 0.183 | *PPM1H* | protein phosphatase, Mg2+/Mn2+ dependent, 1H |
| rs10877945 | 12 | 61732229 | flanking_3UTR |  |  | [A/G] | Fat free mass change (kg/y) | 6.45E-06 | 0.034 | A | 0.304 | *LOC121498* | lactate dehydrogenase A-like 6B pseudogene |
| rs7970034 | 12 | 72363584 | flanking_3UTR |  |  | [T/C] | TNF-α (pg/mL) | 5.81E-06 | 0.026 | G | 0.277 | *RPL31P48* | ribosomal protein L31 pseudogene 48 |
| rs7964120 | 12 | 72374075 | flanking_3UTR |  |  | [A/G] | TNF-α (pg/mL) | 4.50E-06 | 0.027 | A | 0.317 | *RPL31P48* | ribosomal protein L31 pseudogene 48 |
| rs7311660 | 12 | 73340860 | flanking_3UTR |  |  | [A/G] | Eotaxin (pg/mL) | 4.33E-06 | 0.028 | G | 0.397 | *LOC387869* | microtubule-associated protein 1 light chain 3 beta pseudogene |
| rs1368578 | 12 | 74763223 | intron |  |  | [T/C] | HRmax (bpm) | 1.32E-06 | 0.037 | A | 0.072 | *NAP1L1* | nucleosome assembly protein 1-like 1 |
| rs7978060 | 12 | 74766557 | flanking_5UTR |  |  | [T/C] | HRmax (bpm) | 1.70E-06 | 0.035 | G | 0.073 | *NAP1L1* | nucleosome assembly protein 1-like 1 |
| rs11114027 | 12 | 78299379 | intron |  |  | [A/C] | Diet protein (%energy) | 2.84E-06 | 0.032 | A | 0.094 | *SYT1* | synaptotagmin I |
| rs11114112 | 12 | 78336960 | intron |  |  | [A/G] | Diet protein (%energy) | 6.51E-06 | 0.029 | G | 0.090 | *SYT1* | synaptotagmin I |
| rs11116045 | 12 | 82720660 | flanking_3UTR |  |  | [A/G] | Eotaxin (pg/mL) | 2.00E-07 | 0.031 | G | 0.269 | *RPL6P25* | ribosomal protein L6 pseudogene 25 |
| rs1520724 | 12 | 93389608 | flanking_5UTR |  |  | [A/G] | TNF-α (pg/mL) | 8.66E-06 | 0.025 | G | 0.415 | *CCDC41* | coiled-coil domain containing 41 |
| rs483610 | 12 | 97858660 | intron |  |  | [T/C] | Hip circumference change (cm/y) | 7.06E-06 | 0.029 | G | 0.387 | *ANKS1B* | ankyrin repeat and sterile alpha motif domain containing 1B |
| rs11112046 | 12 | 103342322 | flanking_5UTR |  |  | [T/C] | IGF-1 free (ng/mL) | 3.34E-06 | 0.020 | A | 0.179 | *CHST11* | carbohydrate (chondroitin 4) sulfotransferase 11 |
| rs986292 | 12 | 107362971 | flanking_5UTR |  |  | [A/G] | Total cholesterol (mg/dL) | 6.35E-06 | 0.026 | G | 0.399 | *FICD* | FIC domain containing |
| rs11113894 | 12 | 107365993 | flanking_5UTR |  |  | [T/C] | Total cholesterol (mg/dL) | 1.94E-06 | 0.029 | A | 0.397 | *FICD* | FIC domain containing |
| rs7973374 | 12 | 113195065 | flanking_3UTR |  |  | [T/C] | Amylin (pM) | 5.99E-07 | 0.042 | A | 0.221 | *TBX5* | T-box 5 |
| rs10744816 | 12 | 113198649 | flanking_3UTR |  |  | [A/G] | Amylin (pM) | 5.70E-07 | 0.042 | G | 0.234 | *TBX5* | T-box 5 |
| rs725086 | 12 | 113230122 | flanking_3UTR |  |  | [T/C] | Amylin (pM) | 1.34E-06 | 0.040 | A | 0.218 | *TBX5* | T-box 5 |
| rs11067037 | 12 | 113231340 | flanking_3UTR |  |  | [T/C] | Amylin (pM) | 7.39E-06 | 0.035 | G | 0.215 | *TBX5* | T-box 5 |
| rs12322695 | 12 | 115683797 | flanking_5UTR |  |  | [A/C] | Vigorous activity (%awake time) | 6.54E-06 | 0.031 | A | 0.009 | *RNFT2* | ring finger protein, transmembrane 2 |
| rs5745796 | 12 | 116939052 | coding | NONSYN | A13T(NP_031396.1) | [A/G] | IGFBP-3 (ng/mL) | 5.23E-06 | 0.026 | A | 0.032 | *RFC5* | replication factor C (activator 1) 5, 36.5kDa |
| rs7957470 | 12 | 117008809 | intron |  |  | [A/G] | Arm span (cm) | 7.50E-06 | 0.023 | G | 0.259 | *VSIG10* | V-set and immunoglobulin domain containing 10 |
| rs1846598 | 12 | 117633088 | flanking_5UTR |  |  | [T/G] | Moderate activity (%awake time) | 4.95E-06 | 0.028 | C | 0.130 | *KIAA1853* | serine/arginine repetitive matrix 4 |
| rs11069062 | 12 | 117647462 | flanking_5UTR |  |  | [A/G] | Moderate activity (%awake time) | 3.37E-06 | 0.030 | A | 0.124 | *KIAA1853* | serine/arginine repetitive matrix 4 |
| rs10744748 | 12 | 119046219 | flanking_3UTR |  |  | [T/C] | AST (U/L) | 6.15E-06 | 0.031 | G | 0.332 | *GCN1L1* | GCN1 general control of amino-acid synthesis 1-like 1 (yeast) |
| rs10744749 | 12 | 119046265 | flanking_3UTR |  |  | [A/G] | AST (U/L) | 6.15E-06 | 0.031 | G | 0.332 | *GCN1L1* | GCN1 general control of amino-acid synthesis 1-like 1 (yeast) |
| rs11065028 | 12 | 119047357 | flanking_3UTR |  |  | [A/G] | AST (U/L) | 4.14E-06 | 0.032 | G | 0.310 | *GCN1L1* | GCN1 general control of amino-acid synthesis 1-like 1 (yeast) |
| rs2422 | 12 | 119049571 | 3UTR |  |  | [T/C] | AST (U/L) | 7.16E-06 | 0.031 | G | 0.328 | *GCN1L1* | GCN1 general control of amino-acid synthesis 1-like 1 (yeast) |
| rs4298948 | 12 | 120760638 | flanking_3UTR |  |  | [G/C] | Vigorous activity (%awake time) | 2.87E-06 | 0.035 | G | 0.025 | *HPD* | 4-hydroxyphenylpyruvate dioxygenase |
| rs10847255 | 12 | 126062441 | flanking_3UTR |  |  | [A/G] | Light activity (min/d) | 9.92E-06 | 0.028 | A | 0.263 | *LOC121296* | transmembrane protein 132B pseudogene |
| rs1696432 | 12 | 129062929 | flanking_5UTR |  |  | [T/C] | Free T4 (ng/dL) | 1.17E-06 | 0.018 | A | 0.485 | *TMEM132D* | transmembrane protein 132Dpro |
| rs1624802 | 12 | 129063425 | flanking_5UTR |  |  | [A/G] | Free T4 (ng/dL) | 5.44E-07 | 0.022 | A | 0.490 | *TMEM132D* | transmembrane protein 132Dpro |
| rs10773920 | 12 | 130580159 | flanking_3UTR |  |  | [T/G] | Waist circumference (cm) | 7.89E-06 | 0.029 | C | 0.477 |  |  |
| rs4770049 | 13 | 19991604 | intron |  |  | [T/C] | BMI z-score change (SD/y) | 5.33E-06 | 0.028 | G | 0.061 | *CRYL1* | crystallin, lambda 1 |
| rs17064136 | 13 | 20713869 | flanking_5UTR |  |  | [T/C] | C-peptide (ng/mL) | 3.54E-06 | 0.038 | A | 0.037 | *ESRRAP2* | estrogen-related receptor alpha pseudogene 2 |
| rs17356983 | 13 | 20813114 | flanking_3UTR |  |  | [T/C] | Total homocysteine (µmol/L) | 1.59E-06 | 0.029 | G | 0.158 |  |  |
| rs1816752 | 13 | 23878949 | flanking_3UTR |  |  | [T/C] | Urinary free norepinephrine (nmol/d) | 1.89E-06 | 0.048 | G | 0.332 | *PARP4* | poly (ADP-ribose) polymerase family, member 4 |
| rs4770666 | 13 | 23887029 | flanking_3UTR |  |  | [T/C] | Urinary free norepinephrine (nmol/d) | 2.15E-06 | 0.048 | G | 0.349 | *PARP4* | poly (ADP-ribose) polymerase family, member 4 |
| rs1050112 | 13 | 23907297 | coding | NONSYN | P1328T(NP_006428.2) | [A/C] | Urinary free norepinephrine (nmol/d) | 3.43E-06 | 0.046 | A | 0.346 | *PARP4* | poly (ADP-ribose) polymerase family, member 4 |
| rs7995909 | 13 | 23911297 | intron |  |  | [T/C] | Urinary free norepinephrine (nmol/d) | 4.00E-06 | 0.045 | G | 0.346 | *PARP4* | poly (ADP-ribose) polymerase family, member 4 |
| rs1530530 | 13 | 24145286 | flanking_5UTR |  |  | [A/G] | Testosterone (ng/mL) | 6.24E-07 | 0.032 | G | 0.068 | *ATP12A* | ATPase, H+/K+ transporting, nongastric, alpha polypeptide |
| rs12872010 | 13 | 24163271 | coding | SYNON | I317I(NP_001667.4) | [A/G] | Testosterone (ng/mL) | 6.24E-06 | 0.027 | A | 0.065 | *ATP12A* | ATPase, H+/K+ transporting, nongastric, alpha polypeptide |
| rs2483374 | 13 | 24431831 | intron |  |  | [T/G] | Amylin (pM) | 5.17E-06 | 0.025 | C | 0.386 | *TPTE2P1* | transmembrane phosphoinositide 3-phosphatase and tensin homolog 2 pseudogene 1 |
| rs1792023 | 13 | 28266502 | flanking_5UTR |  |  | [A/G] | Diet fat (%energy) | 5.67E-06 | 0.016 | A | 0.499 | *SLC46A3* | solute carrier family 46, member 3 |
| rs954108 | 13 | 28266706 | flanking_5UTR |  |  | [T/C] | Diet fat (%energy) | 1.36E-06 | 0.021 | A | 0.463 | *SLC46A3* | solute carrier family 46, member 3 |
| rs715705 | 13 | 28267467 | flanking_5UTR |  |  | [A/G] | Diet fat (%energy) | 5.15E-06 | 0.016 | G | 0.499 | *SLC46A3* | solute carrier family 46, member 3 |
| rs947247 | 13 | 28267685 | flanking_5UTR |  |  | [A/C] | Diet fat (%energy) | 8.66E-06 | 0.015 | A | 0.496 | *SLC46A3* | solute carrier family 46, member 3 |
| rs9508154 | 13 | 28268726 | flanking_5UTR |  |  | [A/G] | Diet fat (%energy) | 8.27E-06 | 0.016 | G | 0.497 | *SLC46A3* | solute carrier family 46, member 3 |
| rs6490331 | 13 | 28284700 | flanking_5UTR |  |  | [A/C] | Diet fat (%energy) | 8.12E-06 | 0.015 | C | 0.446 | *SLC46A3* | solute carrier family 46, member 3 |
| rs1341329 | 13 | 28294100 | flanking_5UTR |  |  | [A/G] | Diet fat (%energy) | 8.08E-06 | 0.014 | A | 0.446 | *SLC46A3* | solute carrier family 46, member 3 |
| rs2105300 | 13 | 28296616 | flanking_5UTR |  |  | [A/G] | Diet fat (%energy) | 9.57E-06 | 0.015 | A | 0.445 | *SLC46A3* | solute carrier family 46, member 3 |
| rs7322214 | 13 | 28299271 | flanking_5UTR |  |  | [T/G] | Diet fat (%energy) | 9.42E-06 | 0.015 | C | 0.445 | *SLC46A3* | solute carrier family 46, member 3 |
| rs2153666 | 13 | 28309518 | flanking_5UTR |  |  | [A/G] | Diet fat (%energy) | 6.40E-06 | 0.014 | G | 0.444 | *SLC46A3* | solute carrier family 46, member 3 |
| rs61999321 | 13 | 28796783 | coding |  |  | [A/G] | Cystathionine (µmol/L) | 7.65E-06 | 0.029 | A | 0.015 | *MTUS2* | microtubule associated tumor suppressor candidate 2 |
| rs571411 | 13 | 33801092 | flanking_3UTR |  |  | [A/G] | Bone mineral content (kg) | 2.58E-06 | 0.023 | G | 0.015 | *RFC3* | replication factor C (activator 1) 3, 38kDa |
| rs627871 | 13 | 33804055 | flanking_3UTR |  |  | [T/G] | Vigorous activity (%awake time) | 3.31E-06 | 0.031 | A | 0.007 | *RFC3* | replication factor C (activator 1) 3, 38kDa |
| rs683203 | 13 | 33825803 | flanking_3UTR |  |  | [T/C] | Bone mineral content (kg) | 4.50E-06 | 0.023 | A | 0.018 | *RFC3* | replication factor C (activator 1) 3, 38kDa |
| rs12428086 | 13 | 35339099 | intron |  |  | [A/G] | Dinner intake (kcal) | 4.57E-06 | 0.038 | G | 0.374 | *DCLK1* | doublecortin-like kinase 1 |
| rs11838747 | 13 | 35341674 | intron |  |  | [T/G] | Dinner intake (kcal) | 4.62E-06 | 0.038 | A | 0.367 | *DCLK1* | doublecortin-like kinase 1 |
| rs9548050 | 13 | 37261450 | intron |  |  | [T/C] | Protein deposition (kcal/d) | 9.13E-06 | 0.028 | G | 0.240 | *TRPC4* | transient receptor potential cation channel, subfamily C, member 4 |
| rs9548050 | 13 | 37261450 | intron |  |  | [T/C] | Protein (kg/y) | 9.13E-06 | 0.028 | G | 0.240 | *TRPC4* | transient receptor potential cation channel, subfamily C, member 4 |
| rs17064002 | 13 | 42468801 | flanking_5UTR |  |  | [T/C] | Birth weight (kg) | 6.56E-07 | 0.060 | A | 0.007 | *EPSTI1* | epithelial stromal interaction 1 (breast) |
| rs9535307 | 13 | 49189843 | intron |  |  | [A/C] | Fat mass change (kg/y) | 7.74E-06 | 0.031 | A | 0.082 | *KPNA3* | karyopherin alpha 3 (importin alpha 4) |
| rs1127021 | 13 | 49194116 | coding | SYNON | P234P(NP_002258.2) | [T/C] | Fat mass change (kg/y) | 7.74E-06 | 0.031 | G | 0.082 | *KPNA3* | karyopherin alpha 3 (importin alpha 4) |
| rs1239954 | 13 | 50017138 | intron |  |  | [T/C] | TBF-ß1 (pg/mL) | 9.09E-06 | 0.023 | A | 0.006 |  |  |
| rs2806731 | 13 | 53188334 | flanking_3UTR |  |  | [T/C] | Urinary free dopamine (nmol/d) | 5.05E-06 | 0.040 | G | 0.202 | *OLFM4* | olfactomedin 4 |
| rs2593525 | 13 | 68817400 | flanking_3UTR |  |  | [T/G] | HDL (mg/dL) | 2.10E-06 | 0.026 | C | 0.010 | *KLHL1* | kelch-like 1 (Drosophila) |
| rs352236 | 13 | 68921686 | flanking_3UTR |  |  | [T/C] | HDL (mg/dL) | 2.10E-06 | 0.026 | G | 0.010 | *KLHL1* | kelch-like 1 (Drosophila) |
| rs12431307 | 13 | 79542619 | intron |  |  | [T/C] | IL-6 (pg/mL) | 4.99E-06 | 0.032 | G | 0.412 |  |  |
| rs1413568 | 13 | 80970946 | flanking_3UTR |  |  | [A/G] | TNF-α (pg/mL) | 1.83E-06 | 0.029 | A | 0.300 |  |  |
| rs9545740 | 13 | 80973593 | flanking_3UTR |  |  | [T/C] | TNF-α (pg/mL) | 7.06E-07 | 0.032 | A | 0.408 |  |  |
| rs9319064 | 13 | 84149713 | flanking_5UTR |  |  | [A/G] | Arm span (cm) | 3.43E-06 | 0.026 | A | 0.006 | *LOC387939* | microtubule-associated protein 1 light chain 3 beta pseudogene |
| rs16951984 | 13 | 91266365 | intron |  |  | [A/G] | Sedentary activity (%awake time) | 1.24E-06 | 0.032 | A | 0.057 | *GPC5* | glypican 5 |
| rs7328464 | 13 | 91299665 | intron |  |  | [T/C] | Sedentary activity (%awake time) | 3.60E-07 | 0.038 | A | 0.053 | *GPC5* | glypican 5 |
| rs7328464 | 13 | 91299665 | intron |  |  | [T/C] | Sedentary activity (min/d) | 5.28E-06 | 0.031 | A | 0.053 | *GPC5* | glypican 5 |
| rs7981515 | 13 | 91321015 | intron |  |  | [A/C] | Sedentary activity (%awake time) | 9.41E-07 | 0.037 | A | 0.051 | *GPC5* | glypican 5 |
| rs7981515 | 13 | 91321015 | intron |  |  | [A/C] | Sedentary activity (min/d) | 8.29E-06 | 0.031 | A | 0.051 | *GPC5* | glypican 5 |
| rs7998314 | 13 | 93031130 | intron |  |  | [A/C] | Energy storage (kcal/d) | 7.43E-07 | 0.035 | A | 0.487 | *GPC6* | glypican 6 |
| rs7998314 | 13 | 93031130 | intron |  |  | [A/C] | Fat mass change (kg/y) | 3.62E-07 | 0.037 | A | 0.487 | *GPC6* | glypican 6 |
| rs7998314 | 13 | 93031130 | intron |  |  | [A/C] | Fat mass deposition (kcal/d) | 3.48E-07 | 0.038 | A | 0.487 | *GPC6* | glypican 6 |
| rs9788393 | 13 | 93042338 | intron |  |  | [T/C] | Energy storage (kcal/d) | 2.15E-06 | 0.034 | A | 0.465 | *GPC6* | glypican 6 |
| rs9788393 | 13 | 93042338 | intron |  |  | [T/C] | Fat mass change (kg/y) | 1.58E-06 | 0.035 | A | 0.465 | *GPC6* | glypican 6 |
| rs9788393 | 13 | 93042338 | intron |  |  | [T/C] | Fat mass deposition (kcal/d) | 9.31E-07 | 0.037 | A | 0.465 | *GPC6* | glypican 6 |
| rs11070098 | 13 | 94303263 | flanking_5UTR |  |  | [T/C] | Fat mass change (kg/y) | 7.92E-06 | 0.035 | G | 0.204 |  |  |
| rs11070098 | 13 | 94303263 | flanking_5UTR |  |  | [T/C] | Fat mass deposition (kcal/d) | 4.84E-06 | 0.035 | G | 0.204 |  |  |
| rs4771996 | 13 | 96851793 | flanking_3UTR |  |  | [T/C] | Arm span (cm) | 5.59E-06 | 0.025 | G | 0.232 | *MBNL2* | muscleblind-like 2 (Drosophila) |
| rs349114 | 13 | 97001728 | flanking_3UTR |  |  | [T/C] | Arm span (cm) | 3.23E-06 | 0.030 | A | 0.224 | *LOC121906* | proteasome (prosome, macropain) subunit, alpha type, 6 pseudogene 4 |
| rs1854226 | 13 | 97036243 | flanking_3UTR |  |  | [A/G] | Arm span (cm) | 5.13E-06 | 0.029 | A | 0.222 | *LOC121906* | proteasome (prosome, macropain) subunit, alpha type, 6 pseudogene 4 |
| rs9517302 | 13 | 97894035 | intron |  |  | [A/G] | Sedentary&light activity (min/d) | 4.92E-06 | 0.028 | A | 0.342 | *FARP1* | FERM, RhoGEF (ARHGEF) and pleckstrin domain protein 1 (chondrocyte-derived) |
| rs9513627 | 13 | 98994953 | intron |  |  | [T/C] | Diastolic blood pressure (mmHg) | 3.45E-06 | 0.033 | G | 0.057 | *TM9SF2* | transmembrane 9 superfamily member 2 |
| rs9513675 | 13 | 99291161 | intron |  |  | [A/G] | Hip circumference change (cm/y) | 5.04E-06 | 0.032 | G | 0.464 | *CLYBL* | citrate lyase beta like |
| rs1335587 | 13 | 100943561 | intron |  |  | [A/G] | BMI z-score change (SD/y) | 8.51E-06 | 0.031 | G | 0.404 | *ITGBL1* | integrin, beta-like 1 |
| rs16966085 | 13 | 104633171 | flanking_5UTR |  |  | [T/C] | HRmax (bpm) | 2.95E-06 | 0.040 | A | 0.096 | *DAOA* | D-amino acid oxidase activator |
| rs1549059 | 13 | 105047310 | flanking_3UTR |  |  | [A/G] | Energy balance (kcal/d) | 3.87E-06 | 0.032 | A | 0.458 | *DAOA* | D-amino acid oxidase activator |
| rs1253809 | 13 | 105048405 | flanking_3UTR |  |  | [T/C] | Energy balance (kcal/d) | 4.12E-06 | 0.032 | A | 0.469 | *DAOA* | D-amino acid oxidase activator |
| rs9558942 | 13 | 106498219 | flanking_3UTR |  |  | [T/C] | Total glutathione (µmol/L) | 6.66E-06 | 0.031 | G | 0.460 | *FAM155A* | family with sequence similarity 155, member A |
| rs2036707 | 13 | 107282455 | intron |  |  | [T/C] | Fat mass (%) | 7.44E-06 | 0.024 | A | 0.142 | *FAM155A* | family with sequence similarity 155, member A |
| rs984300 | 13 | 108253836 | intron |  |  | [T/C] | Urinary free dopamine (nmol/d) | 3.30E-06 | 0.022 | G | 0.373 | *MYO16* | myosin XVI |
| rs641862 | 13 | 109588233 | flanking_3UTR |  |  | [A/G] | IGFBP-3 (ng/mL) | 7.08E-07 | 0.032 | G | 0.062 | *COL4A1* | collagen, type IV, alpha 1 |
| rs494558 | 13 | 109727163 | intron |  |  | [T/C] | Weight z-score change (SD/y) | 4.66E-08 | 0.045 | G | 0.078 | *COL4A1* | collagen, type IV, alpha 1 |
| rs167890 | 13 | 110247116 | flanking_3UTR |  |  | [T/C] | Diet fat (%energy) | 5.03E-06 | 0.000 | A | 0.042 | *C13orf29* | long intergenic non-protein coding RNA 346 |
| rs10131141 | 14 | 20331573 | flanking_3UTR |  |  | [A/G] | Urinary nitrogen (g/d) | 8.19E-08 | 0.040 | G | 0.280 | *RNASE1* | ribonuclease, RNase A family, 1 (pancreatic) |
| rs4982364 | 14 | 20332271 | flanking_3UTR |  |  | [T/C] | Urinary nitrogen (g/d) | 2.32E-06 | 0.033 | G | 0.436 | *RNASE1* | ribonuclease, RNase A family, 1 (pancreatic) |
| rs4981349 | 14 | 20556227 | coding | SYNON | S277S(NP_057334.1) | [T/C] | Energy balance (kcal/d) | 9.57E-06 | 0.036 | A | 0.010 | *NDRG2* | NDRG family member 2 |
| rs1474476 | 14 | 21677342 | flanking_3UTR |  |  | [A/C] | Urinary free dopamine (nmol/d) | 4.82E-06 | 0.030 | A | 0.108 | *TRAV8-7* | T cell receptor alpha variable 8-7 (non-functional) |
| rs11845134 | 14 | 21852217 | flanking_5UTR |  |  | [A/G] | Weight change (kg/y) | 5.60E-06 | 0.033 | A | 0.181 | *TRAV40* | T cell receptor alpha variable 40 |
| rs12586774 | 14 | 25199406 | flanking_5UTR |  |  | [T/G] | Total cysteine (µmol/L) | 3.99E-07 | 0.033 | A | 0.094 | *LOC401767* | 1-acylglycerol-3-phosphate O-acyltransferase 6 (lysophosphatidic acid acyltransferase, zeta) pseudogene |
| rs12434794 | 14 | 25200279 | flanking_5UTR |  |  | [T/C] | Total cysteine (µmol/L) | 6.94E-06 | 0.030 | A | 0.108 | *LOC401767* | 1-acylglycerol-3-phosphate O-acyltransferase 6 (lysophosphatidic acid acyltransferase, zeta) pseudogene |
| rs1191373 | 14 | 27660868 | flanking_5UTR |  |  | [T/C] | Light activity (min/d) | 9.89E-06 | 0.029 | G | 0.442 | *LOC728755* | uncharacterized LOC728755 |
| rs718545 | 14 | 29559374 | flanking_5UTR |  |  | [A/G] | Diet fat (%energy) | 8.93E-06 | 0.033 | G | 0.139 | *PRKD1* | protein kinase D1 |
| rs12884395 | 14 | 44176382 | flanking_5UTR |  |  | [T/C] | IGFBP-1 (ng/mL) | 9.32E-06 | 0.023 | G | 0.178 | *FSCB* | fibrous sheath CABYR binding protein |
| rs3007103 | 14 | 46436090 | intron |  |  | [T/C] | IL-6 (pg/mL) | 3.38E-07 | 0.041 | A | 0.338 | *MDGA2* | MAM domain containing glycosylphosphatidylinositol anchor 2 |
| rs11627056 | 14 | 46524736 | intron |  |  | [A/G] | IL-6 (pg/mL) | 3.34E-07 | 0.041 | A | 0.281 | *MDGA2* | MAM domain containing glycosylphosphatidylinositol anchor 2 |
| rs17127713 | 14 | 54192668 | flanking_3UTR |  |  | [A/G] | Systolic blood pressure (mmHg) | 2.96E-06 | 0.026 | G | 0.018 | *SAMD4A* | sterile alpha motif domain containing 4A |
| rs17128050 | 14 | 54413629 | intron |  |  | [T/C] | Urinary free dopamine (nmol/d) | 5.22E-06 | 0.030 | G | 0.118 | *GCH1* | GTP cyclohydrolase 1 |
| rs17128050 | 14 | 54413629 | intron |  |  | [T/C] | Urinary free dopamine: creatinine | 2.64E-07 | 0.046 | G | 0.118 | *GCH1* | GTP cyclohydrolase 1 |
| rs3783637 | 14 | 54417868 | intron |  |  | [T/C] | Urinary free dopamine (nmol/d) | 1.30E-06 | 0.034 | A | 0.121 | *GCH1* | GTP cyclohydrolase 1 |
| rs3783637 | 14 | 54417868 | intron |  |  | [T/C] | Urinary free dopamine: creatinine | 6.29E-08 | 0.051 | A | 0.121 | *GCH1* | GTP cyclohydrolase 1 |
| rs8004018 | 14 | 54420446 | intron |  |  | [A/G] | Urinary free dopamine: creatinine | 7.55E-06 | 0.040 | G | 0.139 | *GCH1* | GTP cyclohydrolase 1 |
| rs10151037 | 14 | 55487872 | flanking_5UTR |  |  | [T/G] | Insulin (µU/mL) | 8.30E-06 | 0.029 | A | 0.240 | *LINC00520* | long intergenic non-protein coding RNA 520 |
| rs1427324 | 14 | 58434446 | flanking_5UTR |  |  | [A/G] | IGFBP-3 (ng/mL) | 4.05E-06 | 0.027 | A | 0.343 | *LOC440181* | transmembrane protein 98 pseudogene |
| rs405460 | 14 | 58436068 | flanking_5UTR |  |  | [T/G] | IGFBP-3 (ng/mL) | 5.49E-07 | 0.031 | A | 0.418 | *LOC440181* | transmembrane protein 98 pseudogene |
| rs7141238 | 14 | 60804393 | flanking_3UTR |  |  | [T/G] | Hip circumference change (cm/y) | 2.63E-06 | 0.042 | C | 0.155 | *TMEM30B* | transmembrane protein 30B |
| rs2351807 | 14 | 60821954 | flanking_5UTR |  |  | [A/G] | Hip circumference change (cm/y) | 6.80E-06 | 0.033 | A | 0.131 | *TMEM30B* | transmembrane protein 30B |
| rs11158559 | 14 | 64310702 | intron |  |  | [A/G] | Insulin (µU/mL) | 6.12E-06 | 0.026 | A | 0.148 | *SPTB* | spectrin, beta, erythrocytic |
| rs11158559 | 14 | 64310702 | intron |  |  | [A/G] | Leptin (ng/mL) | 1.36E-06 | 0.031 | A | 0.148 | *SPTB* | spectrin, beta, erythrocytic |
| rs11158577 | 14 | 64666243 | flanking_5UTR |  |  | [A/G] | Eotaxin (pg/mL) | 4.59E-06 | 0.026 | G | 0.207 | *MAX* | MYC associated factor X |
| rs17102423 | 14 | 64674481 | flanking_5UTR |  |  | [A/C] | Eotaxin (pg/mL) | 4.13E-07 | 0.029 | A | 0.200 | *MAX* | MYC associated factor X |
| rs7494064 | 14 | 65357663 | flanking_3UTR |  |  | [A/G] | Head circumference (cm) | 7.88E-06 | 0.035 | G | 0.048 | *FUT8* | fucosyltransferase 8 (alpha (1,6) fucosyltransferase) |
| rs17104363 | 14 | 67009236 | intron |  |  | [T/C] | Dinner intake, adj EER (kcal) | 5.13E-08 | 0.044 | G | 0.050 | *TMEM229B* | transmembrane protein 229B |
| rs11624164 | 14 | 68055910 | intron |  |  | [T/C] | HDL (mg/dL) | 4.62E-06 | 0.031 | A | 0.177 | *RAD51L1* | RAD51 homolog B (S. cerevisiae) |
| rs11850220 | 14 | 71262133 | intron |  |  | [T/C] | TG/HDLC (mmol/L) | 2.02E-06 | 0.031 | A | 0.053 | *SIPA1L1* | signal-induced proliferation-associated 1 like 1 |
| rs17100926 | 14 | 71264154 | intron |  |  | [T/C] | TG/HDLC (mmol/L) | 9.46E-07 | 0.034 | A | 0.049 | *SIPA1L1* | signal-induced proliferation-associated 1 like 1 |
| rs84044 | 14 | 74986217 | intron |  |  | [T/G] | Glucose (mg/dL) | 6.95E-06 | 0.025 | A | 0.175 | *JDP2* | Jun dimerization protein 2 |
| rs2049829 | 14 | 79501910 | intron |  |  | [A/G] | Bike energy expenditure (kcal/min) | 9.85E-06 | 0.033 | G | 0.045 |  |  |
| rs4903976 | 14 | 80831498 | intron |  |  | [T/G] | Urinary free norepinephrine (nmol/d) | 4.73E-06 | 0.033 | C | 0.152 | *STON2* | stonin 2 |
| rs6574638 | 14 | 80836800 | intron |  |  | [A/G] | Urinary free norepinephrine (nmol/d) | 8.25E-06 | 0.032 | G | 0.143 | *STON2* | stonin 2 |
| rs6574644 | 14 | 80848397 | intron |  |  | [T/C] | Urinary free norepinephrine (nmol/d) | 4.66E-06 | 0.034 | G | 0.154 | *STON2* | stonin 2 |
| rs746630 | 14 | 82324958 | coding | NONSYN | Y46*(XP_001132445.1) | [C/G] | Urinary free epinephrine (nmol/d) | 4.24E-06 | 0.039 | G | 0.260 |  |  |
| rs10130259 | 14 | 82412075 | flanking_5UTR |  |  | [T/C] | Diet protein (%energy) | 4.28E-06 | 0.025 | G | 0.066 |  |  |
| rs10130429 | 14 | 82412224 | flanking_5UTR |  |  | [A/G] | Diet protein (%energy) | 4.28E-06 | 0.025 | G | 0.066 |  |  |
| rs7161595 | 14 | 82418887 | flanking_5UTR |  |  | [A/G] | Diet protein (%energy) | 4.28E-06 | 0.025 | G | 0.066 |  |  |
| rs7152554 | 14 | 83667138 | flanking_5UTR |  |  | [A/G] | Diet carbohydrate (g/d) | 5.85E-06 | 0.022 | G | 0.201 |  |  |
| rs17124955 | 14 | 88442504 | flanking_3UTR |  |  | [T/C] | HRmax (bpm) | 9.41E-06 | 0.050 | G | 0.086 | *TTC8* | tetratricopeptide repeat domain 8 |
| rs1741439 | 14 | 91772349 | flanking_3UTR |  |  | [T/C] | Amylin (pM) | 2.68E-06 | 0.035 | A | 0.005 | *CPSF2* | cleavage and polyadenylation specific factor 2, 100kDa |
| rs1998207 | 14 | 94027467 | intron |  |  | [T/G] | Fat mass (%) | 1.04E-06 | 0.038 | A | 0.220 | *SERPINA12* | serpin peptidase inhibitor, clade A (alpha-1 antiproteinase, antitrypsin), member 12 |
| rs1998207 | 14 | 94027467 | intron |  |  | [T/G] | Fat mass (kg) | 3.09E-06 | 0.032 | A | 0.220 | *SERPINA12* | serpin peptidase inhibitor, clade A (alpha-1 antiproteinase, antitrypsin), member 12 |
| rs1998207 | 14 | 94027467 | intron |  |  | [T/G] | Trunk fat mass (kg) | 6.89E-06 | 0.032 | A | 0.220 | *SERPINA12* | serpin peptidase inhibitor, clade A (alpha-1 antiproteinase, antitrypsin), member 12 |
| rs10140797 | 14 | 94035438 | intron |  |  | [T/G] | C-peptide (ng/mL) | 9.76E-06 | 0.028 | A | 0.048 | *SERPINA12* | serpin peptidase inhibitor, clade A (alpha-1 antiproteinase, antitrypsin), member 12 |
| rs11627075 | 14 | 94041995 | intron |  |  | [T/G] | C-peptide (ng/mL) | 3.37E-06 | 0.032 | A | 0.046 | *SERPINA12* | serpin peptidase inhibitor, clade A (alpha-1 antiproteinase, antitrypsin), member 12 |
| rs11625754 | 14 | 94051300 | intron |  |  | [T/C] | C-peptide (ng/mL) | 3.91E-06 | 0.030 | A | 0.042 | *SERPINA12* | serpin peptidase inhibitor, clade A (alpha-1 antiproteinase, antitrypsin), member 12 |
| rs12432260 | 14 | 94918047 | flanking_3UTR |  |  | [A/G] | Trunk fat mass (kg) | 7.47E-06 | 0.032 | A | 0.056 | *C14orf139* | long intergenic non-protein coding RNA 341 |
| rs716315 | 14 | 95079060 | intron |  |  | [A/G] | BMR RQ | 2.08E-06 | 0.034 | A | 0.278 | *GLRX5* | glutaredoxin 5 |
| rs7120 | 14 | 95080342 | 3UTR |  |  | [A/G] | BMR RQ | 1.90E-06 | 0.034 | A | 0.277 | *GLRX5* | glutaredoxin 5 |
| rs2069596 | 14 | 95780932 | flanking_3UTR |  |  | [T/C] | Sedentary&light activity (min/d) | 9.78E-06 | 0.021 | A | 0.102 | *BDKRB2* | bradykinin receptor B2 |
| rs8016620 | 14 | 96463438 | flanking_5UTR |  |  | [A/G] | Total cysteine (µmol/L) | 4.60E-06 | 0.030 | G | 0.033 |  |  |
| rs11623713 | 14 | 98310069 | flanking_3UTR |  |  | [T/C] | Free T3 (pg/mL) | 8.36E-06 | 0.033 | A | 0.043 | *C14orf177* | chromosome 14 open reading frame 177 |
| rs35600665 | 15 | 22292384 | flanking_5UTR |  |  | [T/C] | Diastolic blood pressure (mmHg) | 6.48E-06 | 0.023 | A | 0.151 | *C15orf2* | chromosome 15 open reading frame |
| rs2030062 | 15 | 22685079 | intron |  |  | [T/C] | Amylin (pM) | 2.28E-06 | 0.034 | G | 0.105 | *SNRPN* | small nuclear ribonucleoprotein polypeptide N |
| rs8025093 | 15 | 23154043 | intron |  |  | [T/G] | Bone mineral density (g/cm^2^) | 4.07E-06 | 0.026 | C | 0.003 | *UBE3A* | ubiquitin protein ligase E3A |
| rs2636061 | 15 | 27212047 | flanking_3UTR |  |  | [T/C] | Folate (nmol/L) | 6.72E-06 | 0.021 | A | 0.007 | *FAM189A1* | family with sequence similarity 189, member A1 |
| rs7179495 | 15 | 30687587 | flanking_3UTR |  |  | [T/G] | Sleep duration (min/d) | 1.19E-07 | 0.040 | A | 0.160 | *ARHGAP11A* | Rho GTPase activating protein 11A |
| rs7183147 | 15 | 30699032 | intron |  |  | [A/G] | Sleep duration (min/d) | 1.04E-07 | 0.038 | A | 0.164 | *ARHGAP11A* | Rho GTPase activating protein 11A |
| rs12438775 | 15 | 30708644 | intron |  |  | [A/G] | Sleep duration (min/d) | 2.62E-07 | 0.036 | A | 0.154 | *ARHGAP11A* | Rho GTPase activating protein 11A |
| rs11634439 | 15 | 30710239 | intron |  |  | [A/C] | Sleep duration (min/d) | 6.76E-08 | 0.044 | A | 0.188 | *ARHGAP11A* | Rho GTPase activating protein 11A |
| rs11632524 | 15 | 30713368 | intron |  |  | [A/G] | Sleep duration (min/d) | 1.18E-07 | 0.040 | G | 0.218 | *ARHGAP11A* | Rho GTPase activating protein 11A |
| rs8037818 | 15 | 30714768 | intron |  |  | [A/G] | Sleep duration (min/d) | 4.95E-08 | 0.042 | G | 0.181 | *ARHGAP11A* | Rho GTPase activating protein 11A |
| rs28670903 | 15 | 30719597 | flanking_3UTR |  |  | [T/G] | Sleep duration (min/d) | 1.86E-07 | 0.037 | C | 0.212 | *SCG5* | secretogranin V (7B2 protein) |
| rs8037102 | 15 | 30719942 | flanking_3UTR |  |  | [T/C] | Sleep duration (min/d) | 1.37E-07 | 0.041 | A | 0.182 | *SCG5* | secretogranin V (7B2 protein) |
| rs11635997 | 15 | 30721385 | intron |  |  | [T/C] | Sleep duration (min/d) | 1.84E-06 | 0.036 | A | 0.363 | *SCG5* | secretogranin V (7B2 protein) |
| rs6495130 | 15 | 31597460 | intron |  |  | [A/G] | Waist:height | 9.57E-06 | 0.029 | A | 0.187 | *RYR3* | ryanodine receptor 3 |
| rs8033957 | 15 | 37825725 | intron |  |  | [T/C] | Folate (nmol/L) | 2.34E-06 | 0.026 | G | 0.449 | *FSIP1* | fibrous sheath interacting protein 1 |
| rs2412475 | 15 | 38121868 | flanking_5UTR |  |  | [A/G] | Folate (nmol/L) | 1.54E-06 | 0.034 | A | 0.463 | *SRP14* | signal recognition particle 14kDa (homologous Alu RNA binding protein) |
| rs4924439 | 15 | 38320160 | UTR |  |  | [A/G] | IGFBP-3 (ng/mL) | 1.13E-06 | 0.031 | A | 0.051 | *PAK6* | p21 protein (Cdc42/Rac)-activated kinase 6 |
| rs607541 | 15 | 43722161 | intron |  |  | [A/C] | IGFBP-3 (ng/mL) | 8.13E-06 | 0.025 | C | 0.464 | *SQRDL* | sulfide quinone reductase-like (yeast) |
| rs16961557 | 15 | 46835420 | coding |  |  | [A/G] | Arm span (cm) | 9.63E-06 | 0.033 | G | 0.009 | *CEP152* | centrosomal protein 152kDa |
| rs16961557 | 15 | 46835420 | coding |  |  | [A/G] | Height (cm) | 4.50E-06 | 0.033 | G | 0.009 | *CEP152* | centrosomal protein 152kDa |
| rs2414208 | 15 | 50990593 | flanking_3UTR |  |  | [A/G] | BMR RQ | 2.06E-06 | 0.034 | G | 0.004 | *RPSAP55* | ribosomal protein SA pseudogene 55 |
| rs9920330 | 15 | 51010080 | flanking_3UTR |  |  | [A/G] | BMR RQ | 2.06E-06 | 0.034 | G | 0.004 | *EEF1A1P22* | eukaryotic translation elongation factor 1 alpha 1 pseudogene 22 |
| rs9920982 | 15 | 51011761 | flanking_3UTR |  |  | [A/G] | BMR RQ | 2.06E-06 | 0.034 | G | 0.004 | *EEF1A1P22* | eukaryotic translation elongation factor 1 alpha 1 pseudogene 22 |
| rs17821310 | 15 | 56490156 | flanking_5UTR |  |  | [A/G] | Weight (kg) | 6.25E-06 | 0.026 | G | 0.136 | *LIPC* | lipase, hepatic |
| rs11857380 | 15 | 56499495 | flanking_5UTR |  |  | [T/G] | Weight (kg) | 2.70E-06 | 0.029 | C | 0.131 | *LIPC* | lipase, hepatic |
| rs12595433 | 15 | 61077698 | flanking_3UTR |  |  | [A/G] | Weight z-score (SD) | 5.40E-06 | 0.029 | A | 0.464 | *LOC100287243* | uncharacterized LOC100287243 |
| rs2277554 | 15 | 67115052 | intron |  |  | [T/C] | Fat mass (%) | 2.10E-06 | 0.028 | G | 0.173 | *NOX5* | NADPH oxidase, EF-hand calcium binding domain 5 |
| rs2277554 | 15 | 67115052 | intron |  |  | [T/C] | Leptin (ng/mL) | 4.50E-06 | 0.028 | G | 0.173 | *NOX5* | NADPH oxidase, EF-hand calcium binding domain 5 |
| rs10775200 | 15 | 67118812 | intron |  |  | [T/C] | Fat mass (%) | 1.92E-06 | 0.029 | G | 0.171 | *NOX5* | NADPH oxidase, EF-hand calcium binding domain 5 |
| rs10775200 | 15 | 67118812 | intron |  |  | [T/C] | Leptin (ng/mL) | 3.31E-06 | 0.028 | G | 0.171 | *NOX5* | NADPH oxidase, EF-hand calcium binding domain 5 |
| rs311904 | 15 | 67134000 | intron |  |  | [A/G] | Fat mass (%) | 1.75E-06 | 0.029 | A | 0.164 | *NOX5* | NADPH oxidase, EF-hand calcium binding domain 5 |
| rs311904 | 15 | 67134000 | intron |  |  | [A/G] | Leptin (ng/mL) | 2.17E-06 | 0.030 | A | 0.164 | *NOX5* | NADPH oxidase, EF-hand calcium binding domain 5 |
| rs9783698 | 15 | 72335826 | flanking_3UTR |  |  | [T/C] | Urinary creatinine (mmol/d) | 3.12E-06 | 0.041 | A | 0.150 | *CCDC33* | coiled-coil domain containing 33 |
| rs9783698 | 15 | 72335826 | flanking_3UTR |  |  | [T/C] | Urinary free dopamine (nmol/d) | 9.80E-07 | 0.043 | A | 0.150 | *CCDC33* | coiled-coil domain containing 33 |
| rs7163013 | 15 | 76485814 | flanking_5UTR |  |  | [A/G] | Urinary free epinephrine (nmol/d) | 8.92E-06 | 0.040 | G | 0.351 | *IREB2* | iron-responsive element binding protein 2 |
| rs8040868 | 15 | 76698236 | coding | SYNON | V53V(NP_000734.2) | [A/G] | Sleep energy expenditure adj weight (kcal/d) | 5.95E-08 | 0.048 | G | 0.239 | *CHRNA3* | cholinergic receptor, nicotinic, alpha 3 (neuronal) |
| rs8027587 | 15 | 87050143 | flanking_3UTR |  |  | [A/G] | Systolic blood pressure (mmHg) | 4.81E-06 | 0.030 | G | 0.480 | *ISG20* | interferon stimulated exonuclease gene 20kDa |
| rs12902002 | 15 | 87053522 | flanking_3UTR |  |  | [A/G] | Systolic blood pressure (mmHg) | 5.38E-06 | 0.030 | A | 0.429 | *ISG20* | interferon stimulated exonuclease gene 20kDa |
| rs420017 | 15 | 87418085 | flanking_5UTR |  |  | [A/G] | BMR (kcal/d) | 8.16E-06 | 0.036 | A | 0.336 | *ABHD2* | abhydrolase domain containing 2 |
| rs894157 | 15 | 87969112 | coding | NONSYN | R266C(NP_689472.3) | [A/G] | IGF-1 free (ng/mL) | 9.05E-06 | 0.029 | G | 0.273 | *C15orf42* | chromosome 15 open reading frame 42 |
| rs2238356 | 15 | 90486764 | intron |  |  | [A/C] | IL-6 (pg/mL) | 8.63E-06 | 0.026 | A | 0.436 | *SLCO3A1* | solute carrier organic anion transporter family, member 3A1 |
| rs2238355 | 15 | 90486782 | intron |  |  | [T/C] | IL-6 (pg/mL) | 7.79E-06 | 0.027 | A | 0.435 | *SLCO3A1* | solute carrier organic anion transporter family, member 3A1 |
| rs17612678 | 15 | 91616133 | flanking_3UTR |  |  | [A/G] | TBF-ß1 (pg/mL) | 5.28E-06 | 0.033 | G | 0.275 | *UNQ9370* | IFMQ9370 |
| rs744738 | 15 | 94173633 | flanking_3UTR |  |  | [A/G] | TNF-α (pg/mL) | 5.58E-06 | 0.026 | A | 0.144 | *LOC644192* | uncharacterized LOC644192 |
| rs1513859 | 15 | 95551339 | flanking_3UTR |  |  | [A/G] | Vitamin B12 (pmol/L) | 6.74E-06 | 0.028 | G | 0.011 | *SPATA8* | spermatogenesis associated 8 |
| rs45502703 | 16 | 2047114 | coding |  |  | [C/G] | IGFBP-3 (ng/mL) | 4.71E-06 | 0.027 | G | 0.052 | *TSC2* | tuberous sclerosis 2 |
| rs3751845 | 16 | 3718338 | coding | SYNON | P2237P(NP_004371.2) | [A/G] | IGFBP-3 (ng/mL) | 9.07E-06 | 0.026 | A | 0.053 | *CREBBP* | CREB binding protein |
| rs75825892 | 16 | 4197314 | intron |  |  | [T/C] | Eotaxin (pg/mL) | 5.37E-06 | 0.032 | A | 0.099 | *SRL* | sarcalumenin |
| rs8050907 | 16 | 4586879 | intron |  |  | [T/C] | Total antioxidants (mM) | 6.47E-07 | 0.031 | A | 0.027 | *C16orf96* | chromosome 16 open reading frame 96 |
| rs7195763 | 16 | 4589301 | coding | NONSYN | S966C(XP_296817.7) | [C/G] | Total antioxidants (mM) | 1.46E-06 | 0.029 | G | 0.037 | *C16orf96* | chromosome 16 open reading frame 96 |
| rs13335998 | 16 | 4591922 | flanking_3UTR |  |  | [T/G] | Total antioxidants (mM) | 1.67E-06 | 0.029 | A | 0.029 | *LOC342346* | chromosome 16 open reading frame 96 |
| rs4786816 | 16 | 6088031 | intron |  |  | [T/C] | BMI (kg/m^2^) | 1.94E-06 | 0.033 | A | 0.281 | *RBFOX1* | RNA binding protein, fox-1 homolog (C. elegans) 1 |
| rs4786816 | 16 | 6088031 | intron |  |  | [T/C] | Weight (kg) | 1.87E-06 | 0.031 | A | 0.281 | *RBFOX1* | RNA binding protein, fox-1 homolog (C. elegans) 1 |
| rs7403856 | 16 | 6096327 | intron |  |  | [T/C] | BMI (kg/m^2^) | 2.22E-06 | 0.033 | A | 0.287 | *RBFOX1* | RNA binding protein, fox-1 homolog (C. elegans) 1 |
| rs7403856 | 16 | 6096327 | intron |  |  | [T/C] | BMI z-score (SD) | 9.92E-06 | 0.028 | A | 0.287 | *RBFOX1* | RNA binding protein, fox-1 homolog (C. elegans) 1 |
| rs7403856 | 16 | 6096327 | intron |  |  | [T/C] | Fat mass (kg) | 9.23E-06 | 0.029 | A | 0.287 | *RBFOX1* | RNA binding protein, fox-1 homolog (C. elegans) 1 |
| rs7403856 | 16 | 6096327 | intron |  |  | [T/C] | Weight (kg) | 1.84E-06 | 0.031 | A | 0.287 | *RBFOX1* | RNA binding protein, fox-1 homolog (C. elegans) 1 |
| rs1397142 | 16 | 9250848 | flanking_5UTR |  |  | [A/G] | TBF-ß1 (pg/mL) | 6.01E-06 | 0.028 | A | 0.359 | *RPL21P119* | ribosomal protein L21 pseudogene 119 |
| rs78394940 | 16 | 11129739 | intron |  |  | [T/C] | IGFBP-3 (ng/mL) | 9.39E-07 | 0.031 | A | 0.063 | *CLEC16A* | C-type lectin domain family 16, member A |
| rs78394940 | 16 | 11129739 | intron |  |  | [T/C] | MCP-1 (pg/mL) | 1.71E-06 | 0.044 | A | 0.063 | *CLEC16A* | C-type lectin domain family 16, member A |
| rs737008 | 16 | 11282367 | coding | SYNON | R47R(NP_002752.1) | [T/G] | Fat free mass change (kg/y) | 1.12E-06 | 0.039 | A | 0.352 | *PRM1* | protamine 1 |
| rs737008 | 16 | 11282367 | coding | SYNON | R47R(NP_002752.1) | [T/G] | Protein deposition (kcal/d) | 8.09E-07 | 0.039 | A | 0.352 | *PRM1* | protamine 1 |
| rs737008 | 16 | 11282367 | coding | SYNON | R47R(NP_002752.1) | [T/G] | Protein (kg/y) | 8.09E-07 | 0.039 | A | 0.352 | *PRM1* | protamine 1 |
| rs949429 | 16 | 11283530 | flanking_5UTR |  |  | [T/C] | Protein deposition (kcal/d) | 5.87E-06 | 0.033 | A | 0.340 | *PRM1* | protamine 1 |
| rs949429 | 16 | 11283530 | flanking_5UTR |  |  | [T/C] | Protein (kg/y) | 5.87E-06 | 0.033 | A | 0.340 | *PRM1* | protamine 1 |
| rs1560104 | 16 | 12615709 | flanking_3UTR |  |  | [A/G] | Height z-score change (SD/y) | 4.33E-06 | 0.030 | G | 0.470 | *SNX29* | sorting nexin 29 |
| rs9937036 | 16 | 16638229 | flanking_3UTR |  |  | [T/G] | ALT (U/L) | 7.50E-07 | 0.035 | C | 0.015 | *LOC441750* | chromosome 12 open reading frame 49 pseudogene |
| rs9922516 | 16 | 17932351 | flanking_3UTR |  |  | [T/C] | ALT (U/L) | 8.52E-06 | 0.022 | G | 0.006 |  |  |
| rs4527026 | 16 | 19867821 | flanking_5UTR |  |  | [A/G] | TNF-α (pg/mL) | 4.59E-06 | 0.028 | A | 0.013 | *GPRC5B* | G protein-coupled receptor, family C, group 5, member B |
| rs7195386 | 16 | 24485959 | intron |  |  | [A/G] | Vigorous activity (min/d) | 8.67E-06 | 0.030 | A | 0.477 | *RBBP6* | retinoblastoma binding protein 6 |
| rs9933429 | 16 | 26078441 | flanking_3UTR |  |  | [A/G] | Fat mass (%) | 6.86E-06 | 0.026 | A | 0.305 | *HS3ST4* | heparan sulfate (glucosamine) 3-O-sulfotransferase 4 |
| rs16976587 | 16 | 27122485 | intron |  |  | [T/C] | Folate (nmol/L) | 8.64E-06 | 0.021 | A | 0.006 | *JMJD5* | jumonji domain containing 5 |
| rs205391 | 16 | 27960464 | flanking_3UTR |  |  | [A/G] | Height change (cm/y) | 6.60E-06 | 0.029 | A | 0.096 | *GSG1L* | GSG1-like |
| rs62057608 | 16 | 34539694 | flanking_3UTR |  |  | [T/C] | Diet fat (g/d) | 3.52E-06 | 0.031 | A | 0.028 | *TP53TG3B* | TP53 target 3B |
| rs16961543 | 16 | 58497689 | flanking_5UTR |  |  | [T/G] | Urinary free dopamine: creatinine | 6.09E-06 | 0.032 | C | 0.237 | *LOC644649* | apolipoprotein O pseudogene |
| rs7204371 | 16 | 66251298 | intron |  |  | [T/C] | MCP-1 (pg/mL) | 3.99E-06 | 0.035 | A | 0.048 | *PARD6A* | par-6 partitioning defective 6 homolog alpha (C. elegans) |
| rs11861805 | 16 | 69062860 | intron |  |  | [A/T] | IGFBP-3 (ng/mL) | 8.06E-06 | 0.026 | T | 0.049 | *FUK* | fucokinase |
| rs11861805 | 16 | 69062860 | intron |  |  | [A/T] | MCP-1 (pg/mL) | 5.59E-06 | 0.039 | T | 0.049 | *FUK* | fucokinase |
| rs2042415 | 16 | 73536119 | intron |  |  | [T/C] | Energy storage (kcal/d) | 3.34E-06 | 0.037 | A | 0.146 | *WDR59* | WD repeat domain 59 |
| rs2042415 | 16 | 73536119 | intron |  |  | [T/C] | Fat mass change (kg/y) | 4.96E-06 | 0.038 | A | 0.146 | *WDR59* | WD repeat domain 59 |
| rs2042415 | 16 | 73536119 | intron |  |  | [T/C] | Fat mass deposition (kcal/d) | 7.75E-06 | 0.036 | A | 0.146 | *WDR59* | WD repeat domain 59 |
| rs2042415 | 16 | 73536119 | intron |  |  | [T/C] | Weight change (kg/y) | 7.75E-06 | 0.032 | A | 0.146 | *WDR59* | WD repeat domain 59 |
| rs4888671 | 16 | 76341081 | flanking_3UTR |  |  | [A/G] | Folate (nmol/L) | 7.02E-06 | 0.031 | A | 0.152 | *NUDT7* | nudix (nucleoside diphosphate linked moiety X)-type motif 7 |
| rs1121985 | 16 | 77920580 | flanking_5UTR |  |  | [T/G] | Height z-score change (SD/y) | 6.85E-06 | 0.030 | C | 0.450 |  |  |
| rs7191820 | 16 | 77921315 | flanking_5UTR |  |  | [A/G] | Height z-score change (SD/y) | 6.23E-06 | 0.029 | A | 0.486 |  |  |
| rs11863065 | 16 | 80884306 | flanking_5UTR |  |  | [T/C] | BMI (kg/m^2^) | 9.25E-06 | 0.023 | A | 0.030 | *MPHOSPH6* | M-phase phosphoprotein 6 |
| rs11863065 | 16 | 80884306 | flanking_5UTR |  |  | [T/C] | BMI z-score (SD) | 2.50E-06 | 0.026 | A | 0.030 | *MPHOSPH6* | M-phase phosphoprotein 6 |
| rs11863065 | 16 | 80884306 | flanking_5UTR |  |  | [T/C] | Hip circumference (cm) | 1.21E-07 | 0.037 | A | 0.030 | *MPHOSPH6* | M-phase phosphoprotein 6 |
| rs11863065 | 16 | 80884306 | flanking_5UTR |  |  | [T/C] | Sleep energy expenditure (kcal/d) | 5.38E-06 | 0.032 | A | 0.030 | *MPHOSPH6* | M-phase phosphoprotein 6 |
| rs11863065 | 16 | 80884306 | flanking_5UTR |  |  | [T/C] | Total energy expenditure (kcal/d) | 8.55E-07 | 0.035 | A | 0.030 | *MPHOSPH6* | M-phase phosphoprotein 6 |
| rs11863065 | 16 | 80884306 | flanking_5UTR |  |  | [T/C] | Weight (kg) | 4.41E-07 | 0.031 | A | 0.030 | *MPHOSPH6* | M-phase phosphoprotein 6 |
| rs11863065 | 16 | 80884306 | flanking_5UTR |  |  | [T/C] | Weight z-score (SD) | 1.28E-06 | 0.028 | A | 0.030 | *MPHOSPH6* | M-phase phosphoprotein 6 |
| rs987052 | 16 | 84879960 | flanking_3UTR |  |  | [A/G] | NEFA (mmol/L) | 7.16E-07 | 0.031 | G | 0.438 |  |  |
| rs4083242 | 16 | 84926027 | flanking_3UTR |  |  | [A/G] | Sedentary&light activity (min/d) | 1.39E-07 | 0.034 | A | 0.336 | *LOC732275* | uncharacterized LOC732275 |
| rs11643741 | 16 | 84926100 | flanking_3UTR |  |  | [A/C] | Sedentary&light activity (min/d) | 4.47E-07 | 0.032 | C | 0.336 | *LOC401864* | chloride intracellular channel 1 pseudogene |
| rs3828516 | 16 | 84926807 | flanking_3UTR |  |  | [T/G] | Sedentary&light activity (min/d) | 1.89E-07 | 0.033 | A | 0.335 | *LOC401864* | chloride intracellular channel 1 pseudogene |
| rs1867485 | 16 | 85238532 | flanking_3UTR |  |  | [T/C] | BMR RQ | 2.85E-06 | 0.040 | G | 0.395 | *FOXL1* | forkhead box L1 |
| rs333119 | 17 | 4340247 | flanking_3UTR |  |  | [A/G] | MCP-1 (pg/mL) | 6.03E-06 | 0.024 | A | 0.007 | *SPNS3* | spinster homolog 3 (Drosophila) |
| rs11658587 | 17 | 4842706 | intron |  |  | [T/C] | Sleep duration (min/d) | 5.89E-06 | 0.025 | A | 0.024 | *INCA1* | inhibitor of CDK, cyclin A1 interacting protein 1 |
| rs8076220 | 17 | 11041390 | flanking_5UTR |  |  | [A/G] | Urinary creatinine (mmol/d) | 2.40E-06 | 0.041 | G | 0.074 | *FLJ45455 -- SHISA6* | shisa homolog 6 (Xenopus laevis) |
| rs1877644 | 17 | 11283333 | intron |  |  | [T/C] | QUICKl | 1.81E-06 | 0.033 | A | 0.269 | *SHISA6* | shisa homolog 6 (Xenopus laevis) |
| rs8066099 | 17 | 14830650 | flanking_5UTR |  |  | [A/G] | IL-6 (pg/mL) | 1.75E-06 | 0.030 | A | 0.017 | *RPL23AP76* | ribosomal protein L23a pseudogene 76 |
| rs61744862 | 17 | 17008907 | intron |  |  | [A/G] | IGFBP-3 (ng/mL) | 7.24E-08 | 0.037 | A | 0.041 | *MPRIP* | myosin phosphatase Rho interacting protein |
| rs8077577 | 17 | 18005455 | coding | SYNON | D3162D(NP_057323.3) | [T/C] | Testosterone (ng/mL) | 4.10E-06 | 0.029 | A | 0.110 | *MYO15A* | myosin XVA |
| rs2015336 | 17 | 18014335 | intron |  |  | [A/G] | Testosterone (ng/mL) | 4.10E-06 | 0.029 | G | 0.110 | *MYO15A* | myosin XVA |
| rs11078411 | 17 | 18028819 | coding | SYNON | H179H(NP_060228.3) | [T/C] | Testosterone (ng/mL) | 8.63E-06 | 0.027 | A | 0.121 | *ALKBH5* | alkB, alkylation repair homolog 5 (E. coli) |
| rs11078412 | 17 | 18041343 | intron |  |  | [A/G] | Testosterone (ng/mL) | 7.17E-06 | 0.028 | G | 0.121 | *ALKBH5* | alkB, alkylation repair homolog 5 (E. coli) |
| rs11655588 | 17 | 18048176 | intron |  |  | [A/G] | Testosterone (ng/mL) | 7.17E-06 | 0.028 | G | 0.121 | *ALKBH5* | alkB, alkylation repair homolog 5 (E. coli) |
| rs1563371 | 17 | 18052518 | coding | NONSYN | H407Y(NP_060228.3) | [T/C] | Testosterone (ng/mL) | 7.17E-06 | 0.028 | A | 0.121 | *ALKBH5* | alkB, alkylation repair homolog 5 (E. coli) |
| rs13284 | 17 | 18052855 | 3UTR |  |  | [T/C] | Testosterone (ng/mL) | 6.31E-06 | 0.028 | A | 0.122 | *ALKBH5* | alkB, alkylation repair homolog 5 (E. coli) |
| rs3088233 | 17 | 18053400 | 3UTR |  |  | [T/C] | Testosterone (ng/mL) | 7.17E-06 | 0.028 | A | 0.121 | *ALKBH5* | alkB, alkylation repair homolog 5 (E. coli) |
| rs2013441 | 17 | 19963691 | flanking_3UTR |  |  | [T/C] | Urinary nitrogen (g/d) | 8.81E-06 | 0.032 | A | 0.340 | *SPECC1* | sperm antigen with calponin homology & coiled-coil domains 1 |
| rs4924808 | 17 | 19967140 | flanking_3UTR |  |  | [T/C] | Urinary nitrogen (g/d) | 9.78E-06 | 0.033 | G | 0.346 | *SPECC1* | sperm antigen with calponin homology & coiled-coil domains 1 |
| rs2703806 | 17 | 20048831 | coding | NONSYN | M293L(NP_001028725.1) | [T/A] | Urinary nitrogen (g/d) | 8.81E-06 | 0.032 | T | 0.340 | *SPECC1* | sperm antigen with calponin homology & coiled-coil domains 1 |
| rs2526475 | 17 | 20076808 | intron |  |  | [T/C] | Urinary nitrogen (g/d) | 8.81E-06 | 0.032 | A | 0.340 | *SPECC1* | sperm antigen with calponin homology & coiled-coil domains 1 |
| rs1992562 | 17 | 20159385 | flanking_3UTR |  |  | [A/C] | Urinary nitrogen (g/d) | 8.81E-06 | 0.032 | A | 0.340 | *SPECC1* | sperm antigen with calponin homology & coiled-coil domains 1 |
| rs17780304 | 17 | 27559969 | intron |  |  | [T/C] | Total glutathione (µmol/L) | 5.73E-06 | 0.032 | A | 0.006 | *RHOT1* | ras homolog gene family, member T1 |
| rs4795735 | 17 | 28395981 | intron |  |  | [T/C] | MCP-1 (pg/mL) | 3.54E-06 | 0.023 | A | 0.114 | *ACCN1* | amiloride-sensitive cation channel 1, neuronal |
| rs11654976 | 17 | 28399467 | intron |  |  | [A/G] | MCP-1 (pg/mL) | 6.65E-06 | 0.021 | A | 0.118 | *ACCN1* | amiloride-sensitive cation channel 1, neuronal |
| rs17808461 | 17 | 28417815 | intron |  |  | [T/C] | MCP-1 (pg/mL) | 3.47E-06 | 0.023 | A | 0.117 | *ACCN1* | amiloride-sensitive cation channel 1, neuronal |
| rs2306658 | 17 | 32380425 | 5UTR |  |  | [C/G] | Fat mass (%) | 1.58E-06 | 0.026 | G | 0.249 | *AATF* | apoptosis antagonizing transcription factor |
| rs2306658 | 17 | 32380425 | 5UTR |  |  | [C/G] | Hip circumference (cm) | 9.19E-06 | 0.022 | G | 0.249 | *AATF* | apoptosis antagonizing transcription factor |
| rs9906595 | 17 | 34354906 | coding | NONSYN | Q209R(NP_001008777.1) | [T/C] | Light activity (%awake time) | 4.17E-06 | 0.030 | A | 0.041 | *FBXO47* | F-box protein 47 |
| rs5023 | 17 | 39686255 | coding | NONSYN | S690G(NP_000333.1) | [A/G] | MCP-1 (pg/mL) | 1.04E-06 | 0.042 | G | 0.046 | *SLC4A1* | solute carrier family 4, anion exchanger, member 1 |
| rs6504909 | 17 | 49903124 | flanking_5UTR |  |  | [A/G] | Urinary free dopamine (nmol/d) | 2.60E-06 | 0.045 | G | 0.166 | *TOM1L1* | target of myb1 (chicken)-like 1 |
| rs7226279 | 17 | 50868820 | flanking_5UTR |  |  | [T/G] | Urinary free dopamine: creatinine | 6.88E-06 | 0.031 | A | 0.425 | *MMD* | monocyte to macrophage differentiation-associated |
| rs11653011 | 17 | 50874843 | flanking_5UTR |  |  | [A/G] | Urinary free dopamine: creatinine | 3.23E-06 | 0.038 | G | 0.473 | *MMD* | monocyte to macrophage differentiation-associated |
| rs1292043 | 17 | 55288951 | flanking_3UTR |  |  | [A/G] | Urinary free epinephrine (nmol/d) | 3.25E-06 | 0.033 | G | 0.128 | *TUBD1* | tubulin, delta 1 |
| rs1295923 | 17 | 55315218 | flanking_5UTR |  |  | [T/C] | Urinary free epinephrine (nmol/d) | 3.62E-06 | 0.030 | G | 0.113 | *NDUFB8P2* | NADH dehydrogenase (ubiquinone) 1 beta subcomplex, 8, pseudogene 2 |
| rs2531899 | 17 | 55361975 | intron |  |  | [T/C] | Urinary free epinephrine (nmol/d) | 4.78E-06 | 0.030 | A | 0.131 | *RPS6KB1* | ribosomal protein S6 kinase, 70kDa, polypeptide 1 |
| rs1051424 | 17 | 55379106 | 3UTR |  |  | [A/G] | Urinary free epinephrine (nmol/d) | 3.09E-06 | 0.031 | G | 0.127 | *RPS6KB1* | ribosomal protein S6 kinase, 70kDa, polypeptide 1 |
| rs12600680 | 17 | 55392156 | intron |  |  | [T/C] | Urinary free epinephrine (nmol/d) | 4.80E-06 | 0.030 | G | 0.131 | *RNFT1* | ring finger protein, transmembrane 1 |
| rs17513268 | 17 | 56677825 | intron |  |  | [T/C] | Eotaxin (pg/mL) | 6.63E-06 | 0.025 | G | 0.084 | *BCAS3* | breast carcinoma amplified sequence 3 |
| rs16975985 | 17 | 66145660 | flanking_3UTR |  |  | [A/G] | LDL (mg/dL) | 4.57E-06 | 0.020 | G | 0.079 | *KCNJ2* | potassium inwardly-rectifying channel, subfamily J, member 2 |
| rs1990193 | 17 | 66339684 | flanking_3UTR |  |  | [A/C] | Vitamin B12 (pmol/L) | 9.40E-06 | 0.028 | C | 0.385 | *KCNJ2* | potassium inwardly-rectifying channel, subfamily J, member 2 |
| rs9900972 | 17 | 74380209 | intron |  |  | [A/G] | Head circumference (cm) | 8.10E-06 | 0.021 | A | 0.139 | *TIMP2* | TIMP metallopeptidase inhibitor 2 |
| rs7220048 | 17 | 76733168 | flanking_5UTR |  |  | [A/G] | RQmax | 9.01E-06 | 0.042 | G | 0.496 | *AATK* | apoptosis-associated tyrosine kinase |
| rs621636 | 18 | 312522 | intron |  |  | [A/G] | Total homocysteine (µmol/L) | 3.43E-06 | 0.031 | A | 0.131 | *COLEC12* | collectin sub-family member 12 |
| rs644435 | 18 | 345944 | intron |  |  | [A/G] | Bike energy expenditure (kcal/min) | 3.82E-06 | 0.033 | G | 0.326 | *COLEC12* | collectin sub-family member 12 |
| rs666599 | 18 | 346374 | intron |  |  | [A/G] | Bike energy expenditure (kcal/min) | 5.71E-06 | 0.032 | G | 0.328 | *COLEC12* | collectin sub-family member 12 |
| rs1893154 | 18 | 895125 | flanking_5UTR |  |  | [A/G] | HDL (mg/dL) | 3.39E-06 | 0.028 | A | 0.401 | *ADCYAP1* | adenylate cyclase activating polypeptide 1 (pituitary) |
| rs76205593 | 18 | 3869739 | coding |  |  | [G/C] | IGFBP-3 (ng/mL) | 6.77E-06 | 0.027 | C | 0.070 | *DLGAP1* | discs, large (Drosophila) homolog-associated protein 1 |
| rs11662748 | 18 | 5752618 | intron |  |  | [A/G] | Sleep duration (min/d) | 1.44E-06 | 0.025 | A | 0.114 | *LOC645355* | uncharacterized LOC645355 |
| rs11662297 | 18 | 6658050 | flanking_5UTR |  |  | [T/C] | Leptin (ng/mL) | 3.45E-06 | 0.032 | G | 0.369 | *ARHGAP28* | Rho GTPase activating protein 28 |
| rs10502386 | 18 | 8956144 | flanking_5UTR |  |  | [T/G] | Bone mineral content (kg) | 1.38E-06 | 0.028 | A | 0.373 | *RPS4XP19* | ribosomal protein S4X pseudogene 19 |
| rs10502386 | 18 | 8956144 | flanking_5UTR |  |  | [T/G] | Height (cm) | 5.73E-06 | 0.017 | A | 0.373 | *RPS4XP19* | ribosomal protein S4X pseudogene 19 |
| rs10502386 | 18 | 8956144 | flanking_5UTR |  |  | [T/G] | Sitting height (cm) | 6.74E-06 | 0.013 | A | 0.373 | *RPS4XP19* | ribosomal protein S4X pseudogene 19 |
| rs10502386 | 18 | 8956144 | flanking_5UTR |  |  | [T/G] | Sleep energy expenditure (kcal/d) | 5.38E-06 | 0.029 | A | 0.373 | *RPS4XP19* | ribosomal protein S4X pseudogene 19 |
| rs7235440 | 18 | 20360774 | intron |  |  | [T/C] | IL-6 (pg/mL) | 6.42E-06 | 0.029 | G | 0.460 | *LOC390843* | eukaryotic translation initiation factor 4A3 pseudogene |
| rs7231041 | 18 | 21647102 | flanking_3UTR |  |  | [A/G] | Moderate activity (%awake time) | 4.54E-06 | 0.032 | G | 0.174 | *SS18* | synovial sarcoma translocation, chromosome 18 |
| rs7231041 | 18 | 21647102 | flanking_3UTR |  |  | [A/G] | Moderate activity (min/d) | 2.18E-06 | 0.035 | G | 0.174 | *SS18* | synovial sarcoma translocation, chromosome 18 |
| rs7231041 | 18 | 21647102 | flanking_3UTR |  |  | [A/G] | Moderate&vigorous activity (min/d) | 3.11E-06 | 0.034 | G | 0.174 | *SS18* | synovial sarcoma translocation, chromosome 18 |
| rs1420956 | 18 | 23421943 | intron |  |  | [A/G] | Height z-score change (SD/y) | 4.60E-06 | 0.032 | A | 0.332 | *RBM22P1* | RNA binding motif protein 22 pseudogene 1 |
| rs11564386 | 18 | 23954789 | intron |  |  | [T/C] | Total T3 (ng/dL) | 7.48E-06 | 0.028 | G | 0.017 | *CDH2* | cadherin 2, type 1, N-cadherin (neuronal) |
| rs2155765 | 18 | 23959137 | intron |  |  | [T/C] | Total T3 (ng/dL) | 7.48E-06 | 0.028 | G | 0.017 | *CDH2* | cadherin 2, type 1, N-cadherin (neuronal) |
| rs6508673 | 18 | 26210441 | flanking_3UTR |  |  | [T/C] | Urinary nitrogen (g/d) | 2.79E-06 | 0.039 | A | 0.013 | *DSC3* | desmocollin 3 |
| rs572244 | 18 | 32617532 | flanking_3UTR |  |  | [T/C] | Adiponectin (ng/mL) | 6.14E-06 | 0.025 | A | 0.183 | *FHOD3* | formin homology 2 domain containing 3 |
| rs7226408 | 18 | 32625859 | flanking_3UTR |  |  | [A/G] | Adiponectin (ng/mL) | 4.78E-06 | 0.026 | A | 0.181 | *TPGS2* | tubulin polyglutamylase complex subunit 2 |
| rs7226835 | 18 | 35419303 | flanking_5UTR |  |  | [T/G] | Snack intake (kcal) | 7.75E-06 | 0.043 | A | 0.041 | *RPL7AP66* | ribosomal protein L7a pseudogene 66 |
| rs11659955 | 18 | 35446579 | flanking_5UTR |  |  | [A/G] | Snack intake (kcal) | 7.75E-06 | 0.043 | G | 0.041 | *RPL7AP66* | ribosomal protein L7a pseudogene 66 |
| rs9304270 | 18 | 38191507 | flanking_3UTR |  |  | [T/C] | C-peptide (ng/mL) | 1.70E-06 | 0.030 | A | 0.122 | *PIK3C3* | phosphoinositide-3-kinase, class 3 |
| rs1380836 | 18 | 39734525 | flanking_3UTR |  |  | [T/C] | sICAM-1 (pg/mL) | 1.58E-06 | 0.026 | G | 0.292 | *KRT8P5* | keratin 8 pseudogene 5 |
| rs17798991 | 18 | 45375314 | flanking_3UTR |  |  | [A/C] | Light activity (%awake time) | 6.77E-06 | 0.030 | A | 0.387 | *LIPG* | lipase, endothelial |
| rs1351435 | 18 | 47658962 | flanking_3UTR |  |  | [T/G] | Birth weight (kg) | 4.63E-06 | 0.042 | A | 0.005 | *LOC100287225* | uncharacterized LOC100287225 |
| rs4940203 | 18 | 48446394 | intron |  |  | [T/C] | RQmax | 4.50E-07 | 0.051 | A | 0.294 | *DCC* | DCC deleted in colorectal carcinoma |
| rs2032366 | 18 | 57417351 | flanking_3UTR |  |  | [T/C] | Energy balance (kcal/d) | 5.60E-06 | 0.032 | G | 0.488 | *CDH20* | cadherin 20, type 2 |
| rs12326088 | 18 | 71636557 | flanking_3UTR |  |  | [A/C] | QUICKl | 6.19E-06 | 0.035 | C | 0.467 |  |  |
| rs9956878 | 18 | 71665699 | flanking_3UTR |  |  | [A/C] | LDL (mg/dL) | 1.90E-06 | 0.023 | A | 0.427 |  |  |
| rs9956878 | 18 | 71665699 | flanking_3UTR |  |  | [A/C] | Total cholesterol (mg/dL) | 7.87E-06 | 0.021 | A | 0.427 |  |  |
| rs9959259 | 18 | 71678951 | flanking_3UTR |  |  | [A/G] | Total glutathione (µmol/L) | 8.56E-06 | 0.024 | A | 0.207 |  |  |
| rs8084125 | 18 | 73081746 | flanking_5UTR |  |  | [T/C] | Sitting height (cm) | 6.55E-06 | 0.028 | G | 0.133 | *GALR1* | galanin receptor 1 |
| rs9951602 | 18 | 74758767 | flanking_5UTR |  |  | [A/G] | IGF-1 bound (ng/mL) | 8.52E-06 | 0.025 | A | 0.189 |  |  |
| rs12978500 | 19 | 357934 | 3UTR |  |  | [A/C] | Estradiol (pg/mL) | 1.30E-06 | 0.031 | C | 0.274 | *C2CD4C* | C2 calcium-dependent domain containing 4C |
| rs7253430 | 19 | 1754582 | intron |  |  | [T/G] | TSH (µIU/mL) | 6.65E-06 | 0.021 | A | 0.132 | *ATP8B3* | ATPase, aminophospholipid transporter, class I, type 8B, member 3 |
| rs12977510 | 19 | 3564472 | coding | NONSYN | P490T(XP_938106.1) | [A/C] | IGFBP-3 (ng/mL) | 5.84E-06 | 0.027 | A | 0.046 | *C19orf29* | chromosome 19 open reading frame 2 |
| rs12104221 | 19 | 3748100 | intron |  |  | [A/G] | Total energy expenditure adj for weight (kcal/d) | 2.65E-08 | 0.054 | A | 0.381 | *MATK* | megakaryocyte-associated tyrosine kinase |
| rs2569507 | 19 | 10659904 | intron |  |  | [T/C] | Triglycerides (mg/dL) | 7.01E-06 | 0.020 | G | 0.406 | *ILF3* | interleukin enhancer binding factor 3, 90kDa |
| rs10408126 | 19 | 13106968 | flanking_5UTR |  |  | [A/G] | IGFBP-3 (ng/mL) | 4.10E-06 | 0.027 | A | 0.034 | *NACC1* | nucleus accumbens associated 1, BEN and BTB (POZ) domain containing |
| rs12327666 | 19 | 14000921 | intron |  |  | [A/G] | Diet fat (%energy) | 9.28E-06 | 0.025 | A | 0.031 | *RLN3* | relaxin 3 |
| rs10416963 | 19 | 17623890 | intron |  |  | [A/G] | RQmax | 8.44E-06 | 0.044 | G | 0.487 | *UNC13A* | unc-13 homolog A (C. elegans) |
| rs12610185 | 19 | 19582722 | intron |  |  | [A/G] | Triglycerides (mg/dL) | 3.55E-06 | 0.026 | A | 0.053 | *PBX4* | pre-B-cell leukemia homeobox 4 |
| rs10414075 | 19 | 24030291 | flanking_5UTR |  |  | [T/C] | Amylin (pM) | 9.67E-06 | 0.028 | G | 0.288 | *ZNF254* | zinc finger protein 254 |
| rs10425935 | 19 | 34599947 | intron |  |  | [A/G] | Glucose (mg/dL) | 3.06E-06 | 0.022 | G | 0.099 | *LOC284395* | uncharacterized LOC284395 |
| rs892073 | 19 | 34604134 | intron |  |  | [A/C] | CRP (ng/mL) | 8.10E-06 | 0.030 | A | 0.044 | *LOC284395* | uncharacterized LOC284395 |
| rs33436 | 19 | 35635517 | intron |  |  | [A/G] | Height z-score change (SD/y) | 4.97E-06 | 0.028 | A | 0.442 | *ZNF536* | zinc finger protein 536 |
| rs73039434 | 19 | 38216759 | intron |  |  | [T/G] | Urinary free epinephrine (nmol/d) | 3.28E-06 | 0.035 | C | 0.123 | *RHPN2* | rhophilin, Rho GTPase binding protein 2 |
| rs166988 | 19 | 39025754 | flanking_3UTR |  |  | [T/C] | Energy balance (kcal/d) | 1.35E-06 | 0.038 | G | 0.386 | *KCTD15* | potassium channel tetramerisation domain containing 15 |
| rs11673344 | 19 | 42376806 | intron |  |  | [T/C] | Diet carbohydrate (%energy) | 4.93E-06 | 0.033 | G | 0.411 | *ZNF585B* | zinc finger protein 585B |
| rs30453 | 19 | 44537384 | intron |  |  | [T/G] | Sedentary&light activity (min/d) | 6.92E-06 | 0.016 | C | 0.117 | *SAMD4B* | sterile alpha motif domain containing 4B |
| rs562638 | 19 | 44541629 | intron |  |  | [A/G] | Sedentary&light activity (min/d) | 5.49E-06 | 0.016 | G | 0.117 | *SAMD4B* | sterile alpha motif domain containing 4B |
| rs685125 | 19 | 44541942 | intron |  |  | [A/G] | Sedentary&light activity (min/d) | 5.54E-06 | 0.016 | G | 0.117 | *SAMD4B* | sterile alpha motif domain containing 4B |
| rs36270 | 19 | 44566785 | 3UTR |  |  | [A/G] | Sedentary&light activity (min/d) | 7.26E-06 | 0.015 | G | 0.120 | *SAMD4B* | sterile alpha motif domain containing 4B |
| rs8103033 | 19 | 44861893 | 5UTR |  |  | [A/G] | Urinary nitrogen (g/d) | 8.29E-06 | 0.035 | G | 0.458 | *LGALS17A* | Charcot-Leyden crystal protein pseudogene |
| rs3745618 | 19 | 52267556 | coding | SYNON | L565L(XP_028253.5) | [T/C] | Free T3 (pg/mL) | 1.66E-06 | 0.044 | A | 0.060 | *ZC3H4* | zinc finger CCCH-type containing 4 |
| rs516316 | 19 | 53897957 | flanking_5UTR |  |  | [C/G] | Vitamin B12 (pmol/L) | 2.78E-06 | 0.028 | C | 0.274 | *FUT2* | fucosyltransferase 2 (secretor status included) |
| rs516246 | 19 | 53897984 | intron |  |  | [A/G] | Vitamin B12 (pmol/L) | 2.20E-06 | 0.028 | A | 0.272 | *FUT2* | fucosyltransferase 2 (secretor status included) |
| rs492602 | 19 | 53898229 | coding | SYNON | A68A(NP_000502.4) | [T/C] | Vitamin B12 (pmol/L) | 2.86E-06 | 0.028 | G | 0.273 | *FUT2* | fucosyltransferase 2 (secretor status included) |
| rs681343 | 19 | 53898274 | coding | SYNON | Y83Y(NP_000502.4) | [T/C] | Vitamin B12 (pmol/L) | 2.78E-06 | 0.028 | A | 0.273 | *FUT2* | fucosyltransferase 2 (secretor status included) |
| rs601338 | 19 | 53898486 | coding | NONSYN | W154*(NP_000502.4) | [T/C] | Vitamin B12 (pmol/L) | 2.88E-06 | 0.027 | A | 0.272 | *FUT2* | fucosyltransferase 2 (secretor status included) |
| rs569970 | 19 | 53899530 | 3UTR |  |  | [A/G] | Vitamin B12 (pmol/L) | 7.67E-06 | 0.026 | G | 0.295 | *FUT2* | fucosyltransferase 2 (secretor status included) |
| rs504963 | 19 | 53900677 | 3UTR |  |  | [T/C] | Vitamin B12 (pmol/L) | 4.31E-06 | 0.028 | A | 0.297 | *FUT2* | fucosyltransferase 2 (secretor status included) |
| rs503279 | 19 | 53900822 | 3UTR |  |  | [T/C] | Vitamin B12 (pmol/L) | 3.47E-06 | 0.028 | G | 0.296 | *FUT2* | fucosyltransferase 2 (secretor status included) |
| rs2230245 | 19 | 55601577 | coding | SYNON | T495T(NP_002682.1) | [T/C] | Urinary nitrogen (g/d) | 5.94E-06 | 0.037 | A | 0.057 | *POLD1* | polymerase (DNA directed), delta 1, catalytic subunit 125kDa |
| rs1701930 | 19 | 56111358 | flanking_5UTR |  |  | [A/G] | Urinary free epinephrine (nmol/d) | 1.61E-06 | 0.036 | A | 0.241 | *KLK4* | kallikrein-related peptidase 4 |
| rs254262 | 19 | 59304440 | intron |  |  | [A/G] | Energy storage (kcal/d) | 7.89E-06 | 0.032 | A | 0.468 | *TFPT* | TCF3 (E2A) fusion partner (in childhood Leukemia) |
| rs254262 | 19 | 59304440 | intron |  |  | [A/G] | Weight change (kg/y) | 3.18E-06 | 0.032 | A | 0.468 | *TFPT* | TCF3 (E2A) fusion partner (in childhood Leukemia) |
| rs254262 | 19 | 59304440 | intron |  |  | [A/G] | Weight z-score change (SD/y) | 7.90E-06 | 0.030 | A | 0.468 | *TFPT* | TCF3 (E2A) fusion partner (in childhood Leukemia) |
| rs40167 | 19 | 59374787 | intron |  |  | [T/C] | Height change (cm/y) | 1.75E-07 | 0.040 | A | 0.407 | *MBOAT7* | membrane bound O-acyltransferase domain containing 7 |
| rs40357 | 19 | 59385339 | 5UTR |  |  | [T/C] | Height change (cm/y) | 4.49E-08 | 0.045 | G | 0.391 | *TSEN34* | tRNA splicing endonuclease 34 homolog (S. cerevisiae) |
| rs183716 | 19 | 59385366 | 5UTR |  |  | [C/G] | Height change (cm/y) | 5.97E-08 | 0.044 | G | 0.390 | *TSEN34* | tRNA splicing endonuclease 34 homolog (S. cerevisiae) |
| rs7595 | 19 | 59388891 | coding | SYNON | P265P(NP_076980.2) | [A/G] | Height change (cm/y) | 2.87E-08 | 0.046 | G | 0.389 | *TSEN34* | tRNA splicing endonuclease 34 homolog (S. cerevisiae) |
| rs16986856 | 19 | 61189772 | intron |  |  | [T/G] | Moderate activity (%awake time) | 8.32E-06 | 0.027 | A | 0.454 | *NLRP8* | NLR family, pyrin domain containing 8 |
| rs34863160 | 19 | 61781647 | coding |  |  | [T/C] | Birth weight (kg) | 9.40E-06 | 0.020 | G | 0.002 | *ZNF470* | zinc finger protein 470 |
| rs8106858 | 19 | 63160828 | flanking_3UTR |  |  | [A/G] | Birth weight (kg) | 9.40E-06 | 0.020 | A | 0.005 | *C19orf18* | chromosome 19 open reading frame 18 |
| rs58632700 | 19 | 63683813 | coding |  |  | [T/C] | Free T3 (pg/mL) | 1.28E-07 | 0.054 | A | 0.077 | *ZNF446* | zinc finger protein 446 |
| rs6032887 | 20 | 112083 | flanking_3UTR |  |  | [T/C] | Sleep duration (min/d) | 8.61E-06 | 0.028 | G | 0.068 | *DEFB128* | defensin, beta 128 |
| rs13036722 | 20 | 116020 | flanking_3UTR |  |  | [A/G] | Sleep duration (min/d) | 7.07E-07 | 0.035 | G | 0.067 | *DEFB128* | defensin, beta 128 |
| rs6108038 | 20 | 767662 | intron |  |  | [T/C] | Waist:height | 6.93E-06 | 0.025 | A | 0.040 | *FAM110A* | family with sequence similarity 110, member A |
| rs6050469 | 20 | 2523369 | intron |  |  | [A/G] | Height change (cm/y) | 1.65E-06 | 0.033 | A | 0.011 | *TMC2* | transmembrane channel-like 2 |
| rs2273074 | 20 | 6699089 | coding | NONSYN | A106T(NP_001191.1) | [T/C] | IGFBP-3 (ng/mL) | 1.17E-06 | 0.031 | A | 0.043 | *BMP2* | bone morphogenetic protein 2 |
| rs6056891 | 20 | 9726729 | intron |  |  | [A/G] | Height z-score (SD) | 8.00E-06 | 0.030 | G | 0.268 | *PAK7* | p21 protein (Cdc42/Rac)-activated kinase 7 |
| rs11907106 | 20 | 12030642 | flanking_3UTR |  |  | [A/G] | Sleep duration (min/d) | 3.98E-06 | 0.012 | A | 0.003 | *BTBD3* | BTB (POZ) domain containing 3 |
| rs199307 | 20 | 15125580 | 5UTR |  |  | [A/G] | IGF-1 free (ng/mL) | 7.51E-06 | 0.033 | G | 0.241 | *MACROD2* | MACRO domain containing 2 |
| rs17715103 | 20 | 17317589 | intron |  |  | [A/G] | Total antioxidants (mM) | 2.67E-06 | 0.028 | A | 0.054 | *PCSK2* | proprotein convertase subtilisin/kexin type 2 |
| rs6044834 | 20 | 17384473 | intron |  |  | [A/C] | Total antioxidants (mM) | 7.60E-08 | 0.036 | C | 0.069 | *PCSK2* | proprotein convertase subtilisin/kexin type 2 |
| rs4362633 | 20 | 17393135 | intron |  |  | [A/C] | Total antioxidants (mM) | 2.40E-07 | 0.034 | C | 0.056 | *PCSK2* | proprotein convertase subtilisin/kexin type 2 |
| rs6046346 | 20 | 19750188 | flanking_5UTR |  |  | [T/C] | Free T4 (ng/dL) | 5.39E-06 | 0.035 | A | 0.009 | *RIN2* | Ras and Rab interactor 2 |
| rs6075982 | 20 | 22835257 | flanking_5UTR |  |  | [A/G] | Diet protein (g/d) | 2.89E-06 | 0.036 | A | 0.303 | *SSTR4* | somatostatin receptor 4 |
| rs6082932 | 20 | 22862887 | flanking_5UTR |  |  | [A/C] | Diet protein (g/d) | 5.51E-06 | 0.034 | A | 0.305 | *SSTR4* | somatostatin receptor 4 |
| rs6083004 | 20 | 23028369 | flanking_5UTR |  |  | [A/C] | Leptin (ng/mL) | 1.57E-06 | 0.030 | A | 0.398 | *CD93* | CD93 molecule |
| rs1320561 | 20 | 23045880 | flanking_3UTR |  |  | [A/G] | Leptin (ng/mL) | 1.11E-06 | 0.031 | G | 0.395 | *LOC200261* | uncharacterized LOC200261 |
| rs4815191 | 20 | 23216637 | flanking_5UTR |  |  | [A/G] | Free T3 (pg/mL) | 5.92E-06 | 0.032 | A | 0.202 | *NXT1* | NTF2-like export factor 1 |
| rs8115222 | 20 | 34328654 | flanking_3UTR |  |  | [A/G] | Sedentary&light activity (min/d) | 4.06E-06 | 0.036 | G | 0.015 | *C20orf4* | chromosome 20 open reading frame 4 |
| rs8120917 | 20 | 38077972 | flanking_3UTR |  |  | [T/C] | Testosterone (ng/mL) | 6.43E-06 | 0.027 | A | 0.004 | *MAFB* | v-maf musculoaponeurotic fibrosarcoma oncogene homolog B (avian) |
| rs6017408 | 20 | 42796943 | flanking_3UTR |  |  | [T/C] | IGFBP-3 (ng/mL) | 6.59E-06 | 0.026 | G | 0.406 | *WISP2* | WNT1 inducible signaling pathway protein 2 |
| rs13038016 | 20 | 42803911 | flanking_5UTR |  |  | [T/C] | IGFBP-3 (ng/mL) | 1.26E-06 | 0.030 | G | 0.364 | *KCNK15* | potassium channel, subfamily K, member 15 |
| rs11696845 | 20 | 42804734 | flanking_5UTR |  |  | [T/C] | IGFBP-3 (ng/mL) | 5.26E-07 | 0.032 | A | 0.355 | *KCNK15* | potassium channel, subfamily K, member 15 |
| rs6073535 | 20 | 42807273 | flanking_5UTR |  |  | [A/G] | IGFBP-3 (ng/mL) | 9.75E-07 | 0.031 | G | 0.369 | *KCNK15* | potassium channel, subfamily K, member 15 |
| rs1980577 | 20 | 42815526 | 3UTR |  |  | [T/C] | IGFBP-3 (ng/mL) | 1.88E-06 | 0.028 | A | 0.468 | *RIMS4* | regulating synaptic membrane exocytosis 4 |
| rs6063399 | 20 | 47453225 | intron |  |  | [T/C] | Fat oxidation (%NPEE) | 7.59E-06 | 0.033 | A | 0.450 | *KCNB1* | potassium voltage-gated channel, Shab-related subfamily, member 1 |
| rs2041278 | 20 | 51702402 | flanking_5UTR |  |  | [T/G] | IGFBP-1 (ng/mL) | 5.70E-06 | 0.031 | A | 0.472 | *ZNF217* | zinc finger protein 217 |
| rs2585417 | 20 | 52277206 | flanking_3UTR |  |  | [A/G] | Vigorous activity (min/d) | 4.72E-06 | 0.028 | A | 0.337 | *PFDN4* | prefoldin subunit 4 |
| rs366496 | 20 | 53690857 | flanking_3UTR |  |  | [A/G] | Testosterone (ng/mL) | 9.69E-06 | 0.026 | G | 0.295 | *CBLN4* | cerebellin 4 precursor |
| rs6024938 | 20 | 54542711 | intron |  |  | [T/C] | Urinary free norepinephrine (nmol/d) | 5.72E-06 | 0.038 | A | 0.427 | *C20orf107* | chromosome 20 open reading frame 107 |
| rs4810080 | 20 | 55492133 | flanking_3UTR |  |  | [A/G] | Sedentary&light activity (min/d) | 4.35E-07 | 0.042 | A | 0.451 | *HMG1L1* | high mobility group box 1 pseudogene 1 |
| rs6099650 | 20 | 55492405 | flanking_3UTR |  |  | [T/C] | Sedentary&light activity (min/d) | 2.40E-07 | 0.040 | A | 0.374 | *HMG1L1* | high mobility group box 1 pseudogene 1 |
| rs6099653 | 20 | 55496209 | flanking_3UTR |  |  | [C/G] | Sedentary&light activity (min/d) | 1.23E-07 | 0.047 | C | 0.463 | *HMG1L1* | high mobility group box 1 pseudogene 1 |
| rs3810541 | 20 | 55496725 | flanking_3UTR |  |  | [T/C] | Sedentary&light activity (min/d) | 1.23E-07 | 0.047 | G | 0.463 | *HMG1L1* | high mobility group box 1 pseudogene 1 |
| rs6025590 | 20 | 55503911 | flanking_3UTR |  |  | [T/C] | Sedentary&light activity (min/d) | 3.61E-08 | 0.039 | A | 0.308 | *CTCFL* | CCCTC-binding factor (zinc finger protein)-like |
| rs11086611 | 20 | 55504702 | flanking_3UTR |  |  | [A/G] | Sedentary&light activity (min/d) | 1.68E-07 | 0.045 | G | 0.485 | *CTCFL* | CCCTC-binding factor (zinc finger protein)-like |
| rs2182970 | 20 | 55505059 | flanking_3UTR |  |  | [T/G] | Sedentary&light activity (min/d) | 2.46E-06 | 0.029 | A | 0.346 | *CTCFL* | CCCTC-binding factor (zinc finger protein)-like |
| rs8119092 | 20 | 55926576 | flanking_5UTR |  |  | [T/C] | Diet carbohydrate (%energy) | 8.16E-06 | 0.026 | G | 0.169 | *TMEPAI* | prostate transmembrane protein, androgen induced 1 |
| rs6099847 | 20 | 55935385 | flanking_5UTR |  |  | [T/C] | Diet carbohydrate (%energy) | 6.85E-06 | 0.026 | G | 0.169 | *TMEPAI* | prostate transmembrane protein, androgen induced 1 |
| rs6099854 | 20 | 55945611 | flanking_5UTR |  |  | [A/G] | Diet carbohydrate (%energy) | 8.00E-06 | 0.021 | A | 0.094 | *C20orf85* | chromosome 20 open reading frame 85 |
| rs6100260 | 20 | 56886276 | intron |  |  | [A/G] | Protein deposition (kcal/d) | 9.80E-06 | 0.030 | A | 0.150 | *GNAS* | GNAS complex locus |
| rs6100260 | 20 | 56886276 | intron |  |  | [A/G] | Protein (kg/y) | 9.80E-06 | 0.030 | A | 0.150 | *GNAS* | GNAS complex locus |
| rs6061910 | 20 | 59941577 | coding | SYNON | Q793Q(NP_001785.2) | [A/G] | IGFBP-3 (ng/mL) | 1.17E-07 | 0.037 | A | 0.063 | *CDH4* | CDH4 cadherin 4, type 1, R-cadherin (retinal) |
| rs6061910 | 20 | 59941577 | coding | SYNON | Q793Q(NP_001785.2) | [A/G] | MCP-1 (pg/mL) | 3.88E-06 | 0.044 | A | 0.063 | *CDH4* | CDH4 cadherin 4, type 1, R-cadherin (retinal) |
| rs4925325 | 20 | 59947619 | flanking_3UTR |  |  | [A/G] | LDL (mg/dL) | 6.64E-06 | 0.028 | A | 0.381 | *CDH4* | CDH4 cadherin 4, type 1, R-cadherin (retinal) |
| rs17854409 | 20 | 60961939 | coding |  |  | [T/C] | Cortisol (ng/mL) | 9.26E-06 | 0.022 | G | 0.055 | *TCFL5* | transcription factor-like 5 (basic helix-loop-helix) |
| rs2315656 | 20 | 61888781 | intron |  |  | [T/C] | CRP (ng/mL) | 3.74E-06 | 0.031 | G | 0.395 | *ZBTB46* | zinc finger and BTB domain containing 46 |
| rs2823606 | 21 | 16376543 | intron |  |  | [T/C] | Sleep RQ | 1.31E-06 | 0.035 | A | 0.171 | *C21orf34* | long intergenic non-protein coding RNA 478 (LINC00478) |
| rs2823607 | 21 | 16378484 | intron |  |  | [T/G] | Sleep RQ | 6.63E-06 | 0.031 | A | 0.142 | *C21orf34* | long intergenic non-protein coding RNA 478 (LINC00478) |
| rs9975788 | 21 | 16386686 | intron |  |  | [A/G] | Sleep RQ | 7.51E-08 | 0.043 | A | 0.176 | *C21orf34* | long intergenic non-protein coding RNA 478 (LINC00478) |
| rs9983980 | 21 | 16394237 | intron |  |  | [A/G] | Sleep RQ | 8.30E-07 | 0.036 | A | 0.170 | *C21orf34* | long intergenic non-protein coding RNA 478 (LINC00478) |
| rs11088565 | 21 | 16396102 | intron |  |  | [A/G] | Sleep RQ | 9.51E-08 | 0.043 | A | 0.179 | *C21orf34* | long intergenic non-protein coding RNA 478 (LINC00478) |
| rs9978366 | 21 | 16401149 | intron |  |  | [T/G] | Sleep RQ | 4.66E-06 | 0.032 | C | 0.166 | *C21orf34* | long intergenic non-protein coding RNA 478 (LINC00478) |
| rs2823612 | 21 | 16401424 | intron |  |  | [T/C] | Sleep RQ | 7.25E-07 | 0.037 | G | 0.177 | *C21orf34* | long intergenic non-protein coding RNA 478 (LINC00478) |
| rs2823614 | 21 | 16402763 | intron |  |  | [T/G] | Sleep RQ | 1.37E-06 | 0.035 | A | 0.161 | *C21orf34* | long intergenic non-protein coding RNA 478 (LINC00478) |
| rs11909251 | 21 | 16404258 | intron |  |  | [T/G] | Sleep RQ | 1.27E-07 | 0.042 | A | 0.171 | *C21orf34* | long intergenic non-protein coding RNA 478 (LINC00478) |
| rs2823615 | 21 | 16405004 | intron |  |  | [T/A] | Sleep RQ | 5.28E-08 | 0.044 | A | 0.178 | *C21orf34* | long intergenic non-protein coding RNA 478 (LINC00478) |
| rs2823616 | 21 | 16405929 | intron |  |  | [A/G] | Sleep RQ | 1.45E-06 | 0.035 | A | 0.161 | *C21orf34* | long intergenic non-protein coding RNA 478 (LINC00478) |
| rs4818252 | 21 | 16409990 | intron |  |  | [T/C] | Sleep RQ | 9.64E-06 | 0.030 | G | 0.149 | *C21orf34* | long intergenic non-protein coding RNA 478 (LINC00478) |
| rs1735884 | 21 | 20742279 | flanking_5UTR |  |  | [A/G] | Fat free mass change (kg/y) | 6.63E-06 | 0.035 | G | 0.214 |  |  |
| rs1735884 | 21 | 20742279 | flanking_5UTR |  |  | [A/G] | Protein deposition (kcal/d) | 4.00E-06 | 0.036 | G | 0.214 |  |  |
| rs1735884 | 21 | 20742279 | flanking_5UTR |  |  | [A/G] | Protein (kg/y) | 4.00E-06 | 0.036 | G | 0.214 |  |  |
| rs2268241 | 21 | 33702920 | intron |  |  | [A/G] | QUICKl | 4.99E-06 | 0.029 | A | 0.201 | *IFNGR2* | interferon gamma receptor 2 |
| rs12483148 | 21 | 35935491 | flanking_3UTR |  |  | [A/G] | Diastolic blood pressure (mmHg) | 4.64E-06 | 0.029 | G | 0.038 | *SETD4* | SET domain containing 4 |
| rs3804024 | 21 | 40915577 | intron |  |  | [T/C] | Arm span (cm) | 9.10E-07 | 0.029 | G | 0.035 | *DSCAM* | Down syndrome cell adhesion molecule |
| rs3804024 | 21 | 40915577 | intron |  |  | [T/C] | Height z-score (SD) | 3.00E-06 | 0.028 | G | 0.035 | *DSCAM* | Down syndrome cell adhesion molecule |
| rs220299 | 21 | 42375831 | intron |  |  | [T/C] | HRmax (bpm) | 4.23E-07 | 0.051 | G | 0.373 | *UMODL1* | uromodulin-like 1 |
| rs2851391 | 21 | 43360473 | intron |  |  | [T/C] | Total homocysteine (µmol/L) | 4.15E-06 | 0.022 | G | 0.365 | *CBS* | cystathionine-beta-synthase |
| rs701428 | 22 | 18608542 | flanking_3UTR |  |  | [T/C] | MCP-1 (pg/mL) | 2.35E-06 | 0.036 | G | 0.472 | *RTN4R* | reticulon 4 receptor |
| rs739310 | 22 | 25381299 | intron |  |  | [T/C] | Diet protein (%energy) | 6.47E-06 | 0.013 | G | 0.227 | *MIAT* | myocardial infarction associated transcript (non-protein coding) |
| rs2071748 | 22 | 34107618 | intron |  |  | [T/C] | HRmax (bpm) | 2.99E-06 | 0.046 | A | 0.273 | *HMOX1* | heme oxygenase (decycling) 1 |
| rs2285112 | 22 | 34119263 | intron |  |  | [A/G] | HRmax (bpm) | 4.36E-06 | 0.040 | G | 0.285 | *HMOX1* | heme oxygenase (decycling) 1 |
| rs739031 | 22 | 35538095 | intron |  |  | [T/G] | Bone mineral content (kg) | 2.18E-06 | 0.034 | C | 0.205 | *PVALB* | parvalbumin |
| rs762920 | 22 | 35542482 | intron |  |  | [A/C] | Bone mineral content (kg) | 3.45E-06 | 0.030 | A | 0.190 | *PVALB* | parvalbumin |
| rs1048310 | 22 | 38243228 | 3UTR |  |  | [A/C] | Urinary nitrogen (g/d) | 9.61E-06 | 0.028 | C | 0.279 | *SMCR7L* | Smith-Magenis syndrome chromosome region, candidate 7-like |
| rs5767992 | 22 | 46566917 | flanking_5UTR |  |  | [T/C] | Insulin (µU/mL) | 7.64E-06 | 0.033 | A | 0.067 | *RP11-191L9.1* | uncharacterized LOC400932 |
| rs13054085 | 22 | 47008848 | flanking_3UTR |  |  | [T/C] | Total homocysteine (µmol/L) | 1.48E-06 | 0.032 | A | 0.236 |  |  |
| rs2272836 | 22 | 48924677 | coding | SYNON | R795R(NP_061868.1) | [A/C] | Urinary nitrogen (g/d) | 6.78E-06 | 0.029 | A | 0.377 | *MOV10L1* | Mov10l1, Moloney leukemia virus 10-like 1, homolog (mouse) |
| rs7511006 | 22 | 49018386 | intron |  |  | [T/C] | Urinary nitrogen (g/d) | 1.19E-06 | 0.042 | G | 0.244 | *TUBGCP6* | tubulin, gamma complex associated protein 6 |
| rs5771109 | 22 | 49020698 | intron |  |  | [A/G] | Urinary nitrogen (g/d) | 1.28E-06 | 0.042 | G | 0.268 | *TUBGCP6* | tubulin, gamma complex associated protein 6 |
| rs5771270 | 22 | 49024992 | coding |  |  | [A/G] | Urinary nitrogen (g/d) | 3.90E-06 | 0.037 | A | 0.245 | *TUBGCP6* | tubulin, gamma complex associated protein 6 |
| rs131788 | 22 | 49323620 | flanking_5UTR |  |  | [T/C] | Fat mass (kg) | 8.54E-06 | 0.035 | A | 0.189 | *ODF3B* | outer dense fiber of sperm tails 3B |
| Abbreviations: SNP, single nucleotide polymorphism; chr, chromosome; MGA, measured genotype analysis; syn, synonomous; UTR, untranslated region;IGFBP-3, insulin-like growth factor binding protein-3; BMI, body mass index; ALT, alanine aminotransferase; AST, aspartate aminotransferase;TGF-β1, transforming growth factor-beta 1;HOMA-IR, homeostatic model assessment-insulin resistance; LDL, low-density lipoprotein; sICAM-1, soluble intercellular adhesion molecule-1; QUICK1, quantitative insulin sensitivity check index; T3, triiodothyronine;T4, free thyroxine; NEFA, nonesterified fatty acids; MCP-1, monocyte chemotactic protein-1; IL-6, interluekin-6; RQ, respiratory quotient; TNF-α, tumor necrosis factor-alpha; NPEE, nonprotein energy expenditure; BMR, basal metabolic rate; TG, triglycerides; HDL, high-density lipoprotein cholesterol; RANTES, regulated upon activation, normal T-cell expressed and secreted; CRP, C-reactive protein; TSH, thyroid stimulating hormone; EER, estimated energy requirement; VO2max, maximum oxygen consumption. | | | | | | | | | | | | | |
